# Species-Specific Susceptibility of Planktonic and Biofilm Forming Candida Strains to Cyclodextrin-Encapsulated Essential Oils

**DOI:** 10.3390/pharmaceutics18040508

**Published:** 2026-04-20

**Authors:** Sourav Das, Farid Baradarbarjastehbaf, Aliz Sára Szokolics, Génesis Katherine Dela Campos, Zoltán Gazdag, Aleksandar Széchenyi, Attila Miseta, Gábor L. Kovács, Tamás Kőszegi

**Affiliations:** 1 Molecular Medicine Research Group, Szentágothai Research Center, University of Pécs, Ifjúság u. 20, 7624 Pécs, Hungary; kovacs.l.gabor@pte.hu (G.L.K.); koszegi.tamas@pte.hu (T.K.); 2 Institute of Pharmaceutical Technology and Biopharmacy, Faculty of Pharmacy, University of Pécs, Rókus u. 2., 7624 Pécs, Hungary; baradarbarjastehbaf.farid@pte.hu (F.B.); szokolics.aliz@edu.pte.hu (A.S.S.); szechenyi.aleksandar@gytk.pte.hu (A.S.); 3 Institute of Biology, Faculty of Sciences, University of Pécs, Ifjúság u. 6, 7624 Pécs, Hungary; dela.campos.genesis@pte.hu; 4 Department of Molecular Biology and Microbiology, Institute of Biology, University of Pécs, Ifjúság u. 6, 7624 Pécs, Hungary; gazdag@gamma.ttk.pte.hu; 5 Department of Chemistry, University of Osijek, Ulica cara Hadrijana 8/A, 31000 Osijek, Croatia; 6 Department of Laboratory Medicine, Medical School, University of Pécs, Ifjúság út 13, 7624 Pécs, Hungary; attila.miseta@aok.pte.hu

**Keywords:** Candida biofilm, essential oils, randomly methylated beta-cyclodextrin, oxidative stress, treatment efficacy ranking, sublethal concentration, antifungal susceptibility

## Abstract

**Background/Objectives:** Essential oils (EOs) have multi-target antifungal activity, but their translation is limited by volatility and poor aqueous dispersibility. Randomly methylated β-cyclodextrin (RAMEB) inclusion may enhance effective exposure and thereby alter susceptibility, stress responses, and biofilm outcomes in a species-dependent manner. This study quantified species-specific planktonic and biofilm susceptibility to four EOs and their RAMEB complexes across clinically relevant Candida species. **Methods:** Lavender (L), lemon balm (B), peppermint (P), and thyme (T) oils and their RAMEB complexes (RL, RB, RP, and RT) were tested against *C. albicans* and non-albicans Candida. Susceptibility thresholds were used to derive phase plasticity metrics. Functional inhibition was assessed via planktonic metabolism/viability and established biofilm metabolism/viability/biomass. Mechanistic signatures were captured by ROS/RNS measurements and a qPCR analysis of antioxidant genes (*CAT1*, *GPX1*, and *SOD1*) was performed. Mixed-effects models and multivariate/unsupervised and interpretable classification approaches (k-means, PCA, and CRT) were used to integrate endpoints and stratify response phenotypes. **Results:** Susceptibility thresholds were strongly species-structured (lowest MIC_90_/EC_10_ for *C. albicans*; higher thresholds and broader sublethal windows in non-albicans species). RAMEB complexation produced formulation-dependent shifts in efficacy, with RT emerging as the most consistent broad-spectrum inhibitory condition across compartments. Biofilm biomass was comparatively insensitive even when viability was suppressed, indicating a decoupling of structural biomass from biocidal activity. Mechanistic signatures were broadly conserved across species and linked to antioxidant-program engagement, with *CAT1*-related rules contributing to responder/tolerant classification. **Conclusions:** Integrating MIC/EC plasticity with functional and mechanistic markers supports the rational selection of EO formulations; RAMEB complexation, particularly RT, prioritizes candidates for further pharmaceutical optimization while highlighting species-specific vulnerabilities.

## 1. Introduction

Infections caused by Candida spp. remain a major clinical burden across mucosal disease and invasive candidiasis, while therapeutic management is increasingly complicated by interspecies variability in antifungal susceptibility, stress tolerance, and biofilm formation [1,2,3,4]. Although *Candida albicans* (Ca) remains the most frequently isolated species, non-albicans Candida (NAC) such as *Candida dubliniensis* (Cd), *Candida krusei* (Ck), and *Candida tropicalis* (Ct) are prominent in many settings and can exhibit reduced susceptibility or distinct tolerance-associated phenotypes [4,5,6]. These biological differences have direct implications for antifungal development and therapeutic selection because they shape concentration–effect relationships, increase the likelihood of persistence under marginal exposure, and facilitate transitions into biofilm-associated states that are intrinsically less responsive to treatment [7,8,9].

Essential oils (EOs) and EO-derived constituents are intensively investigated as antifungal candidates due to their multi-target activity, including the perturbation of fungal membrane organization, disruption of metabolic processes, interference with signaling, and induction of oxidative/nitrosative stress responses [10,11]. Among commonly studied botanical sources, thyme oil is frequently associated with strong antifungal activity linked to phenolic monoterpenes (e.g., thymol/carvacrol) and membrane-active mechanisms, while peppermint, lavender, and lemon balm oils have also been reported to inhibit Candida growth and biofilm-related phenotypes, albeit with more variable potency and composition-dependent effects [12,13,14,15]. However, the translation of EO bioactivity into pharmaceutically standardized products is limited by formulation challenges such as low aqueous solubility, volatility, chemical instability, and batch-to-batch variability in chemical profiles, which together can produce inconsistent effective exposure at the microorganism–medium interface and complicate comparisons across species, strains, and treatment conditions [10,16]. Consequently, pharmaceutics-enabling technologies that improve solubilization and stabilize EO constituents are essential to evaluate antifungal potential in a reproducible manner and to enable the rational selection of candidates for further development [17,18].

Cyclodextrin inclusion complexation is a widely used strategy to increase apparent solubility, improve dispersion, and stabilize hydrophobic compounds in aqueous systems [17,18,19]. Randomly methylated β-cyclodextrin (RAMEB) has a high complexation capacity for lipophilic molecules and is frequently used to enhance dissolution and delivery in water-based environments [17]. In the context of EO-based antifungal development, RAMEB complexation could increase the effective exposure of volatile and poorly soluble EO constituents and thereby enhance antifungal activity [16,20,21,22]. At the same time, changes in exposure kinetics and stress intensity may also alter cellular response programs. Sublethal stress can activate antioxidant defenses and other protective pathways that may contribute to tolerance-like behavior and persistence under treatment [8,9]. Therefore, it is not sufficient to compare uncomplexed EOs and RAMEB-EO (REO) formulations using a single inhibition endpoint; efficacy should be evaluated together with mechanistic signatures indicating whether exposures yield durable inhibitory phenotypes or preferentially engage stress-adaptation programs [5,9].

A further translational consideration is that antifungal responses are frequently non-linear across concentration ranges, and inhibitory effects can differ markedly from sub-inhibitory responses that still reshape physiology and survival [8,9]. This can be captured by jointly considering inhibitory thresholds (e.g., minimum inhibitory concentration-based metrics) and lower-effect thresholds (e.g., EC_10_). The separation between these thresholds provides a quantitative descriptor of dose–response “phase plasticity”, reflecting how readily a population shifts between strongly inhibited and partially surviving states across the concentration–effect curve. Various species or treatments with larger minimum inhibitory concentration–effective concentration (MIC-EC) separations may be more prone to persistence under marginal exposure—an issue that is particularly relevant for complex mixtures such as EOs and for formulated systems where dispersion and release kinetics may vary over time [8,20].

In this study, four essential oils, lavender (L), lemon balm (B), peppermint (P), and thyme (T), and their corresponding RAMEB-complexed formulations (RL, RB, RP, and RT) against four clinically relevant Candida species (Ca, Cd, Ck, and Ct) have been investigated. Susceptibility thresholds like minimum inhibitory concentration (MIC_90_) and effective concentration (EC_10_) were integrated, and derived phase plasticity metrics were combined with survival and functional inhibition endpoints under planktonic conditions (metabolic activity and viability) and during premature biofilm formation (biomass, metabolism, and viability), together with mechanistic readouts of oxidative/nitrosative stress (ROS/RNS) and antioxidant gene programs (*CAT1*, *GPX1*, and *SOD1*) [23,24]. To connect these layers and identify coherent response patterns, multivariate and unsupervised analyses were applied (e.g., clustering and principal component analysis) together with interpretable classification approaches (e.g., decision trees) to define treatment signatures, stratify phenotypes consistent with susceptibility versus tolerance-like behavior, and detect patterns consistent with adaptive stress engagement [25,26].

Despite increasing interest in cyclodextrin-based delivery systems for essential oils, most previous antifungal studies have focused primarily on improving the physicochemical stability, solubility, or overall antimicrobial potency of the encapsulated compounds. Comparatively little attention has been given to how cyclodextrin complexation reshapes the biological response landscape across different Candida species or how formulation-driven changes influence cellular stress pathways and phenotypic outcomes. In the present study, we therefore combine formulation evaluation with a species-resolved pharmacological and mechanistic framework. Using four clinically relevant Candida species (*C. albicans*, *C. tropicalis*, *C. krusei*, and *C. dubliniensis*), we integrate susceptibility thresholds (MIC_90_ and EC_10_), phase plasticity analysis, planktonic and biofilm functional endpoints, oxidative–nitrosative stress profiling (ROS/RNS), and antioxidant gene responses (*CAT1*, *GPX1*, and *SOD1*). Multivariate modeling and machine-learning-based inference are further applied to define treatment exposure signatures and identify predictors of inhibitory phenotypes. By linking formulation strategy with redox biology and species-dependent pharmacodynamics, this work provides a mechanistic framework for understanding how cyclodextrin-encapsulated essential oils modulate antifungal efficacy across the Candida genus.

This work is structured around three testable hypotheses relevant to pharmaceutics-guided antifungal development. First, RAMEB complexation modifies EO exposure and can enhance antifungal efficacy for specific oils, but the magnitude and direction of this effect will be species-dependent and not all RAMEB–EO formulations will outperform their uncomplexed counterparts [16,20]. Second, Candida species differ in susceptibility thresholds and phase plasticity, and these baseline differences contribute to species-enriched tolerance-like response patterns under specific EO or RAMEB–EO exposures [4,5,6,8,24]. Third, combined stress markers (ROS/RNS) and antioxidant gene responses (*CAT1*, *GPX1*, and *SOD1*) define mechanistic exposure signatures that can predict inhibitory phenotype classes, distinguishing strong inhibition from weak inhibition/tolerance-like behavior (a condition in which cells exhibit reduced susceptibility at sub-inhibitory concentrations without a shift in MIC, reflecting adaptive or stress-response-mediated survival) and from response patterns consistent with stress adaptation [8,24,27,28]. The essential oils investigated in this study were selected based on their documented antifungal activity and their chemically distinct dominant constituents, which represent different classes of bioactive terpenoids. Lavender (*Lavandula angustifolia*) oil is primarily composed of linalool and linalyl acetate, compounds known to disrupt fungal membrane integrity and interfere with ergosterol-dependent membrane stability. Lemon balm (*Melissa officinalis*) oil contains high levels of citral isomers (geranial and neral) together with phenylpropanoid components such as estragole, which have been associated with antifungal and oxidative stress-inducing activity [27]. Peppermint (*Mentha piperita*) oil is dominated by menthol and menthone, monoterpenes capable of altering membrane permeability and mitochondrial function in fungal cells [8,24]. Thyme (*Thymus vulgaris*) oil, in contrast, contains phenolic monoterpenes such as thymol and carvacrol, which exhibit strong antifungal activity through membrane disruption, proton gradient collapse, and the induction of oxidative stress. The selection of these oils therefore allowed the investigation of chemically diverse antifungal mechanisms while evaluating how RAMEB-mediated encapsulation modulates their biological activity across different Candida species.

Accordingly, our objectives were to quantify species-level susceptibility and phase plasticity, determine treatment and treatment × species effects on planktonic and premature biofilm endpoints, stratify treatments into mechanistic signature classes using multivariate analyses, integrate endpoints into treatment rankings relevant to formulation selection, and identify EO candidates that warrant further pharmaceutical optimization versus those that require caution due to limited efficacy or signatures consistent with stress-adaptation programs [16,17,29,30].

## 2. Materials and Methods

### 2.1. Materials

All reagents and consumables used in this study were of analytical or spectroscopic grade unless otherwise specified. Sterile 96-well microtiter plates were employed for antifungal susceptibility testing, metabolic assays, and live/dead staining (SPL Life Sciences, Pocheon-si, Republic of Korea), while biofilm-related experiments were carried out in specialized flat-bottom plates (Sarstedt AG & Co. KG, Nümbrecht, Germany). Acetic acid, adenine, agar-agar, crystal violet (CV), dextrose, peptone, potassium phosphate monobasic, and yeast extract were all obtained from Reanal Labor (Budapest, Hungary).

Amphotericin B (AM), acetonitrile, dimethyl sulfoxide (DMSO), fluconazole (FL), formic acid, menadione (MN), potassium chloride, propidium iodide (PI), resazurin sodium salt, RPMI 1640 medium, sodium chloride, sodium phosphate dibasic, SYBR Green I nucleic acid gel stain and 3-Morpholinopropane sulfonic acid were all obtained from Merck Kft. (Budapest, Hungary). Intracellular ribonucleic acid (RNA) was purified using the Macherey–Nagel NucleoSpin RNA kit (AKTIVIT Kft., Budapest, Hungary). Nuclease-free molecular-grade water and a High-Capacity cDNA reverse transcription kit (Applied Biosystems; ref. 4368814) were obtained from ThermoFisher Scientific (Budapest, Hungary). Quantitative PCR was performed using BioSyGreen Mix Hi-ROX (2×) (PCR Biosystems, London, UK). All the oligonucleotides were purchased from IDT (Coralville, IA, USA). Ibidi µ-Slide 18 Well polymer microscopy slides (ref. 81811, Zenon Bio Ltd., Szeged, Hungary) were used for the cultivation of the fungal biofilms intended for qualitative analysis.

All the essential oils (EO) namely lavender (L), lemon balm (B), peppermint (P), and thyme (T) essential oils were obtained from the commercial supplier Panarom kft. (Budapest, Hungary). The oils were subsequently encapsulated in randomly methylated β-cyclodextrin (RAMEB) by CycloLab Cyclodextrin Research & Development Laboratory (Budapest, Hungary) to produce the RAMEB–essential oil inclusion complexes used in the biological experiments. All the experiments were performed using a single standardized batch of essential oils and corresponding RAMEB inclusion complexes to ensure consistency across experimental conditions. Ultrapure water with conductivity < 1.0 µS was utilized throughout all experimental workflows. Detailed information on the suppliers of the consumables can be found in the Supplementary Section (Table S1).

### 2.2. Instruments Used in the Experiments

All experimental procedures were performed using standard laboratory instrumentation. Centrifugation was carried out using a Hettich Rotina 420R benchtop centrifuge (Auro-Science Consulting Kft., Budapest, Hungary). The automated washing and aspiration steps during the microplate-based biofilm assays were performed using an ELx50 microplate washer (Biotech Hungary Kft., Szigetszentmiklós, Hungary). The instrument was configured for gentle bottom washing and controlled aspiration to minimize the disruption of adherent fungal biofilms. Incubations were conducted using a Sanyo microbiological incubator shaker and a Thermo Scientific Heraeus B12 microbiological incubator (Auro-Science Consulting Kft., Budapest, Hungary). Optical measurements were acquired using a PerkinElmer EnSpire multimode plate reader, a Thermo Fisher Scientific NanoDrop 2000 spectrophotometer, and a Thermo MultiSkan MCC Type 355 plate reader (Auro-Science Consulting Ltd., Budapest, Hungary). Molecular-biology-based assays were performed using an Applied Biosystems Veriti 9902 96-well thermal cycler, and Applied Biosystems StepOnePlus Real-Time PCR system (Auro-Science Consulting Ltd., Budapest, Hungary). The microscopic figures were achieved with an Olympus CKX53 cell culture microscope equipped with a Tucsen TrueChrome Metrics 1080P HDMI microscope camera (Auro-Science Consulting Ltd., Budapest, Hungary). For the chromatographic separation and mass spectroscopic detection, the Thermo Dionex Ultimate 3000 UHPLC system (Dionex, Sunnyvale, CA, USA), Thermo Q Exactive Focus quadrupole-Orbitrap high-resolution mass spectrometer, Thermo Accucore RP-MS column, and Accucore guard column (Thermo Fisher Scientific, Waltham, MA, USA) were used throughout the study.

### 2.3. Chemical Characterization of Essential Oils

The chemical characterization of the essential oils was supported by supplier-provided GC-MS data for the selected formulations (R, T, and P). For the lavender essential oil (L), compositional profiling was performed using HPLC-MS based on the previously reported methods in Refs. [31,32]. Detailed analytical conditions are provided in the Supplementary Materials (Supplementary Method SM1).

### 2.4. Candida Albicans and Non-Albicans Candida Strains Selected for Experimental Evaluation

The Candida strains used in this study were obtained from the Szeged Microbiology Collection (SZMC, Szeged, Hungary) and were maintained at the Department of General and Environmental Microbiology, Institute of Biology, University of Pécs. The panel included *Candida albicans* (Ca) SZMC 1372, 1423, 1426 (Ca1372, Ca1423 and Ca1424); *Candida dubliniensis* (Cd) SZMC 1470, 1471 (Cd1470 and Cd1471); *Candida krusei* SZMC 779, 1447 (Ck779 and Ck1447); and *Candida tropicalis* (Ct) SZMC 1368, 1432 (Ct1368 and Ct1432). These isolates were employed for determining the minimum inhibitory concentration (MIC_90_), the minimum effective concentration (EC_10_), and oxidative stress, and for assessing the inhibition of biofilm eradication by the test compounds.

All the strains were cultured on standard yeast-extract-peptone-dextrose (YPD) agar composed of 1% (*w*/*v*) peptone, 0.5% (*w*/*v*) yeast extract, 2% (*w*/*v*) dextrose, and 1.5% (*w*/*v*) agar in distilled water. Detailed information on the suppliers of the consumables can be found in Supplementary Section (Table S1).

### 2.5. Antimicrobial Susceptibility Testing

The minimum inhibitory concentration (MIC_90_) determination was carried out using a modified broth microdilution method based on a previously established protocol (based on CLSI M27-A2) [33,34]. The EO-RAMEB complexes and the corresponding EOs were prepared in a log2 dilution format to yield a final concentration range of 0.03–62.5 µg/mL. For each assay, 100 µL of overnight cultured standardized fungal inoculum (~2–5 × 10^3^ cells/mL) prepared in modified RPMI 1640 medium was dispensed into sterile 96-well microtiter plates. An equal volume of each test sample (TS)/antifungal reference controls (RC) in a squared dilution format was then added.

The AM and FL served as RC and were tested across 0.0001–3.125 µg/mL and 0.058–30 µg/mL final concentrations respectively. Wells containing inoculated medium without test compounds served as growth controls (GCs), whereas uninoculated medium functioned as the sterility control (SC). The DMSO-only with a final solvent concentration of 1% served as the dilution control (DC) throughout the experiments.

The plates were incubated for 48 h at 35 °C, after which fungal growth was assessed spectrophotometrically (Thermo MultiSkan MCC Type 355 plate reader) by measuring optical density at 595 nm. The optical density values were normalized to the untreated growth control (set to 100%). The minimum inhibitory concentration (MIC_90_) values defined as the concentrations resulting in ≥90% growth inhibition were calculated by fitting the data to a non-linear dose response model. Each experiment consisted of three technical replicates and was repeated across six independent biological replicates. The AM and FL were included as standard antifungal reference controls to benchmark the activity of EO and RAMEB-EO formulations and to provide clinically relevant comparison points throughout the experiments.

### 2.6. Quantification of Minimum Effective Concentration (EC_10_) Using a Survival-Response Assay

The EC_10_ was determined using an adapted survival-based microdilution method derived from previously established protocols [35,36]. Mid-log-phase Candida cultures (approximately 2–5 × 10^5^ cells/mL), grown overnight under shaking conditions, were harvested, washed with sterile PBS (pH 7.43), and pelleted by centrifugation at 1000× *g* for 5 min. The cells were then resuspended in YPD medium and standardized to approximately 2.5 × 10^7^ cells/mL.

The standardized cultures were exposed to a wide concentration range of EO-RAMEB and EO component–RAMEB complexes (final concentration per well: 0.03–62.5 µg/mL). The AM and FL were included as antifungal reference controls (RC) and tested at final concentrations of 0.0001–3.125 µg/mL and 0.058–30 µg/mL, respectively. Exposure to TS and RC was limited to a maximum duration of 60 min, with sampling performed at 0, 0.25, 0.5, and 1 h.

Post-treatment, the samples underwent two-step serial dilutions to reach a cumulative dilution factor of 1950. Subsequently, 50 µL aliquots were spread onto YPD agar and incubated at 30 °C for 24 h, after which colony-forming units (CFU/mL) were quantified. No dedicated quenching step was applied; however, dilution reduces residual treatment concentrations prior to colony recovery. The CFU counts were normalized to untreated cultures, which were defined as 100% survival. A non-linear dose–response model was applied to calculate the EC_10_, defined as the concentration resulting in 90% survival after 1 h of exposure.

Uninoculated media and aliquoted YPD agar plates served as sterility controls (SCs) and growth controls (GCs), respectively. All the experiments were conducted with three technical replicates and repeated in six independent biological experiments. Kinetic time-course data were summarized as area under the curve (AUC) values to enable multivariable comparisons with endpoint measurements. The resulting AUC-derived metrics were subsequently Z-transformed (within species/strain, as applicable) to harmonize scaling across endpoints for integrated analyses.

### 2.7. Acute Exposure-Based RNS and ROS Generation

Reactive nitrogen species (RNS) and reactive oxygen species (ROS) production were assessed using previously published protocols [35,36,37] with minor modifications. Briefly, mid-log-phase Candida cultures (approximately 2–5 × 10^7^ cells/mL) were exposed to TS at their respective EC_10_ concentrations (see Supplementary Table S4) or to 0.5 mM menadione (MN) as an oxidative stress positive control (OC) for 1 h at 30 °C in modified RPMI 1640 medium. Following centrifugation and resuspension in PBS, the cells were incubated with 10 µM dihydrorhodamine 123 (DHR 123) for RNS detection or 15 µM dihydroethidium (DHE) for ROS detection for an additional 1 h at 30 °C in the dark.

Fluorescence was measured using a PerkinElmer EnSpire multimode microplate reader (Auro-Science Consulting Ltd., Budapest, Hungary) at excitation/emission wavelengths of 495/530 nm for rhodamine generation and 520/600 nm for 2-hydroxyethidium generation. Oxidative stress levels were expressed as the percentage increase relative to untreated controls (UCs). The AM and FC at their respective EC_10_ concentrations were included as antifungal reference controls (RC) throughout the experiments. All assays were performed in triplicate and repeated across six independent experiments.

### 2.8. Molecular Expression of the Oxidative Genes

The Candida planktonic cells were harvested and treated according to the protocols described in Section 2.6 and Section 2.7. To prepare for the quantitative polymerase chain reaction (qPCR) analysis of gene expression, total RNA was extracted and purified from approximately 50 mg (wet weight) of the collected planktonic samples. Before the RNA extraction, the samples were pelleted by centrifugation at 500× *g* for 5 min in sterile microcentrifuge tubes. The RNA extraction and purification process was rigorously followed according to the manufacturer’s guidelines for the NucleoSpin RNA purification kit (AKTIVIT Kft., Budapest, Hungary). The quality of the isolated RNA was subsequently verified by measuring the absorbance ratio at 260/280 nm using a Thermo Fisher Scientific NanoDrop 2000 spectrophotometer.

The quantitative polymerase chain reaction (qPCR) was performed using oligonucleotide primers specifically designed for the Candida species genes *CAT1*, *GPX1*, and *SOD1* (serving as target genes), alongside *RDN18* (employed as the reference gene). The primer design was carried out using NCBI Nucleotide BLAST+ (version 2.17.0), employing *Candida albicans* reference sequences as models to identify homologous regions in *Candida tropicalis*, *Candida dubliniensis*, and *Candida krusei*. Searches were performed using MEGABLAST or BLASTn, depending on sequence availability. The retrieved sequences were aligned in MEGA v7 to identify conserved regions, and the primers were designed using Primer-BLAST and Primer3Plus (version 3.3.0) [38,39,40]. Primer quality was evaluated in silico, and de-generate primers were manually designed when sequence data were limited using species-specific gene sequences retrieved from the NCBI databases for each Candida species. Primer details, including accession numbers, sequences, amplicon sizes, and annealing temperatures, together with final primer sequences, are provided in Supplementary Table S2.

For quantitative gene expression analysis, total RNA was reverse transcribed into complementary DNA (cDNA) utilizing the High-Capacity cDNA reverse transcription kit. A minimum of 2 µg of purified total RNA served as the template for each reaction. The 2× reverse transcription master mix, comprising RT buffer, dNTP mix, MultiScribe reverse transcriptase, and nuclease-free molecular-grade water, was prepared on ice following the manufacturer’s specified proportions. Each 20 µL reverse transcription reaction was meticulously assembled by combining 10 µL of the pre-prepared 2X master mix with 10 µL of the RNA sample, followed by thorough but gentle mixing. Reactions were maintained on ice until loaded into the thermal cycler. The cDNA synthesis was conducted in an Applied Biosystems Verti 9902 96-well thermal cycler with the following cycling parameters: an initial incubation at 25 °C for 10 min, followed by reverse transcription at 37 °C for 2 h, and a final enzyme inactivation step at 85 °C for 5 min. The newly synthesized cDNA was then stored at temperatures between −15 °C and −25 °C to ensure its integrity for further downstream applications [41,42,43,44].

The quantitative PCR (qPCR) was executed in 10 µL reactions on a StepOne PCR machine utilizing BioSyGreen Mix Hi-Rox (2×). Each reaction mixture contained 5 µL of BioSyGreen Mix Hi-Rox (2×), 0.8 µL of combined forward and reverse primers (400 nM final concentration for each), 0.5 µL of cDNA template, and 3.7 µL of nuclease-free water. Reaction assembly involved first combining the master mix components (BioSyGreen Mix, primers, and water), followed by the addition of the cDNA template that was synthesized from the treated and untreated planktonic, ensuring thorough homogenization before thermal cycling. Relative gene expression was quantified using the comparative Ct (ΔΔCt) method. Expression levels were reported as fold changes and normalized to *RDN18*, which served as the endogenous housekeeping control. All the experiments were performed in triplicates and repeated in six independent experiments [37,38].

### 2.9. Impact of Test Agents on Planktonic Cells and Established Biofilms

The prolonged antifungal (killing) activity of the TS, RC, and OC against planktonic Candida and non-albicans Candida species was evaluated under time-dependent treatment conditions. Mid-logarithmic-phase planktonic Candida cultures were standardized to approximately 2–5 × 10^7^ cells/mL and exposed to the TS, RC, and OC at their respective EC_10_ concentrations (see Supplementary Table S4). Treated suspensions were incubated at 30 °C for up to 16 h, with samples collected at 0, 2, 4, 8, and 16 h to monitor time-dependent changes in viability and cellular metabolic activity, as described previously (Section 2.6 and Section 2.7).

The effects of the treatments on established biofilms were assessed using a modified version of previously published protocols [35,36]. Briefly, 200 mid-log-phase Candida cultures (approximately 2–3 × 10^5^ cells/mL) prepared in modified RPMI 1640 medium were dispensed into sterile 96-well plates and incubated for 24 h at 30 °C to allow biofilm formation. Following biofilm establishment, non-adherent cells were removed by gentle washing with sterile PBS (pH 7.43). Under these conditions, the resulting biofilms represent mature, established biofilm structures, enabling the evaluation of treatment efficacy against developed biofilm communities. The wells were then replenished with 200 µL of fresh modified RPMI medium containing the TS, RC, and OC (see Supplementary Table S4) at their respective EC_10_ concentrations (µg/mL) and incubated for an additional 24 h at 30 °C.

Throughout the assay, modified RPMI medium served as the backgroundcontrol (BC); untreated inoculated wells served as the untreated biofilm control (UBC); AM, FL-, and MN-treated wells were included as the RC and OC at their respective EC_10_ concentrations; and 1% DMSO was used as the dilution control (DC). For the planktonic, kinetic time-course data were reduced to area under the curve (AUC) values to facilitate multivariable comparisons with single-timepoint endpoint measurements. The resulting AUC-derived metrics were then Z-transformed within species/strain, as appropriate, to harmonize scaling across the endpoints for integrated analyses. All the experiments were performed in triplicates across six independent experiments.

#### 2.9.1. Metabolic Activity and Viability of Planktonic Candida and Non-Albicans Candida

Following treatment and sampling as described in Section 2.6 and Section 2.7 (planktonic cultures) and Section 2.9 (biofilms), the samples were processed for metabolic activity and viability analyses. The planktonic cells were centrifuged at 1000× *g* for 5 min, washed twice with PBS (pH 7.43), and centrifuged again, whereas the biofilm biomass was washed twice with PBS directly in the wells after treatment and sampling (Section 2.9).

Metabolic activity was assessed using a previously published resazurin-based assay [35,36]. Briefly, 200 µL of resazurin was added to each sample to obtain a final concentration of 12.5 µM, followed by incubation for 40 min at 30 °C in the dark. Fluorescence was measured at excitation/emission wavelengths of 560/590 nm using a fluorescent multimode microplate reader. Metabolic activity was expressed as a percentage by normalizing fluorescence values to the untreated control (UC) at the corresponding sampling points (Section 2.6, Section 2.7 and Section 2.9), with the UC defined as 100% metabolic activity. The PBS alone and PBS containing resazurin served as background(BC) and background noise controls (NCs), respectively.

In parallel, the time-dependent cell viability of both planktonic and biofilm-associated Candida populations was evaluated using an optimized SYBR Green I/propidium iodide (PI) dual-staining assay [36,45]. The samples obtained according to Section 2.6, Section 2.7 and Section 2.9 were centrifuged (planktonic cells) or washed in situ (biofilms), resuspended in PBS, and stained with a freshly prepared dye mixture containing SYBR Green I (20 µL of a 10,000× stock diluted 1:100 in PBS) and PI (4 µL of a 20 mM DMSO stock diluted 1:500 in PBS). One hundred microliters of staining solution were added per well, followed by incubation for 15 min at room temperature in the dark with gentle agitation. Fluorescence was recorded at 490/525 nm for SYBR Green I and 530/620 nm for PI. Green-to-red fluorescence ratios were used to estimate the proportion of non-viable cells and were normalized to the untreated control (UC); PBS served as the background control(BC) while the SYBR Green I/PI mix was considered the noise control (NC). All experiments were performed in triplicates in six independent experiments.

#### 2.9.2. Quantification of Total Biofilm-Associated Fungal Biomass

Biofilm biomass was quantified using a crystal violet (CV) staining assay adapted from previously published methods [36]. Following the treatment of established biofilms (Section 2.9), the supernatant was removed and the wells were gently rinsed twice with PBS (pH 7.43) using a BioTek ELx50 microplate washer (Biotech Hungary Kft., Szigetszentmiklós, Hungary) configured for gentle aspiration and dispensing to preserve adherent biofilm integrity (see Appendix A for detailed washing program setup). The adherent biofilms were then fixed with 2% (*v*/*v*) formalin prepared in PBS and stained with 0.13% (*w*/*v*) CV for 20 min at room temperature. Excess stain was discarded, and wells were washed twice with PBS using the same automated washing conditions to remove unbound dye.

To solubilize the retained CV, 1% (*w*/*v*) sodium dodecyl sulfate (SDS) prepared in ethanol was added to each well and incubated overnight. Absorbance was measured at 600 nm using a Multiskan EX microplate reader. Biofilm biomass was expressed as a percentage by normalizing absorbance values to the untreated biofilm control (UBC), which was defined as 100% biofilm biomass. CV-only microtiter wells served as background noise controls (NCs). All assays were performed in triplicate and repeated across six independent experiments.

In addition to quantitative microplate measurements, a qualitative assessment of the biofilm architecture was performed using crystal violet stained samples prepared under comparable conditions. The biofilms were established on microscope chamber slides and treated as described above (Section 2.9). Following staining with 0.13% (*w*/*v*) CV, the samples were gently washed twice with PBS to remove excess dye and resuspended in 100 µL PBS without dye solubilization. The biofilm structures were then examined by bright-field microscopy at 20× magnification. These images provide a representative visualization of treatment-dependent changes in biofilm coverage and structural organization and are presented alongside quantitative biomass measurements.

### 2.10. Statistical Analyses

All analyses and data visualization were performed in IBM SPSS Statistics (version 29.0.2.0 (20), IBM LCC, Budapest, Hungary) and OriginPro 2018 (Version 95E, OriginLab Corp., Northampton, MA, USA), respectively. Unless otherwise stated, quantitative outcomes were analyzed on standardized values to enable cross-endpoints integration and to control baseline species-dependent scaling differences. The experiments were conducted using independent biological replicates, with the total number of observations indicated for each analysis (N = 162 across species for susceptibility endpoints; *n* = 6 with three technical replicates in each experiment). Data distribution was assessed prior to statistical testing.

The endpoints used for mechanistic profiling (ROS, RNS, *CAT1*, *GPX1*, and *SOD1*) and for integrated efficacy modeling (planktonic and biofilm functional readouts) were standardized by Z-transformation within each species prior to analysis. This transformation expressed each observation as a deviation from the species-specific means in units of the species-specific standard deviation. Accordingly, the standardized values represent relative within-species shifts rather than absolute between-species differences, allowing the integration of heterogeneous endpoints while controlling for baseline scaling differences across Candida species. The MIC_90_ and EC_10_ were retained on their original concentration scale and analyzed separately as primary susceptibility thresholds. These parameters reflect biologically interpretable concentration–response endpoints and were therefore not standardized. Statistical inference was conducted using two-sided tests with a predefined significance threshold of α = 0.01. When multiple pairwise comparisons were performed, *p*-values were adjusted using Bonferroni correction to control the family-wise error rate (FWER).

For the MIC_90_ and EC_10_ comparisons, nonparametric Kruskal–Wallis tests were applied due to the non-normal distribution of concentration–response data, followed by multiple-comparison-adjusted pairwise comparisons. Between-species differences in MIC_90_ and EC_10_ were tested using Kruskal–Wallis tests due to the non-normal distributions and heteroscedasticity typical of dilution-based susceptibility measures. To quantify phase plasticity, two derived metrics were computed from the raw thresholds: phase shift (EC_10_-MIC_90_) and phase distance (EC_10_/MIC_90_; log-transformed where appropriate). These derived plasticity metrics were summarized by species and compared across species using Kruskal–Wallis tests with post hoc pairwise comparisons when indicated.

For the multi-factorial analyses, linear mixed-effects models (REML estimation) were used, with treatment and species as fixed effects and experimental repeat as a random factor. Post hoc comparisons were performed using estimated marginal means (EMMeans). The models used Satterthwaite approximations for denominator degrees of freedom. The fixed effects included treatment, species, and their interaction to quantify species dependence of treatment efficacy. Estimated marginal means (EMMeans) with 95% confidence intervals were extracted for treatment and species effects; treatment comparisons were conducted using Bonferroni-adjusted pairwise tests (a two-sided significance threshold of *p* < 0.001 was applied throughout). For the sections focused on ranking experimental candidates, analyses were repeated on the experimental panel only (excluding reference controls with distinct concentration regimes), whereas mechanistic profiling and mode-of-action analyses included controls to anchor mechanistic space [46,47].

To generate an integrated treatment ranking across endpoints, inhibition-coded versions of key efficacy outcomes were created where needed (sign direction aligned such that larger negative shifts on the Z scale corresponded to stronger inhibitory effects). The endpoint-level inhibition measures were then combined into a composite efficacy score. The composite score was analyzed using linear mixed-effects models with treatment, species, and their interaction. The treatment ranking was based on the EMMeans of the composite score with Bonferroni-adjusted comparisons; additional paired contrasts were used to compare each essential oil (EO) with its corresponding RAMEB-complexed formulation (EO vs. RAMEB-EO) to quantify formulation-dependent shifts.

To classify exposure phenotypes, unsupervised clustering was performed on a species-wise averaged, standardized multivariate response profile that characterizes how a specific Candida species biologically responds to a specific EO or RAMEB–EO treatment (aggregated exposure signatures). Raw replicate-level data were first aggregated to strain × treatment signatures (means) to avoid overweighting repeated measures in clustering and classification. Inhibition-coded endpoint signatures were then subjected to k-means clustering (k = 3), yielding response classes interpreted as (i) susceptible/strong inhibition, (ii) hormetic/adaptive (mixed or stimulatory patterns), and (iii) tolerant/resistant (weak inhibition). Cluster validity was assessed by the inspection of cluster centers and by separation in the clustering output. Species enrichment of response classes was evaluated using Pearson χ^2^ tests on contingency tables (response class by species). For mechanistic stress-response stratification, k-means clustering was also applied to treatment × species mean signatures across ROS/RNS and antioxidant genes to define mechanistic exposure classes; cluster membership distributions were tested across treatments and species using χ^2^ tests.

To identify the mechanistic predictors of inhibitory phenotypes, response classes were collapsed into a binary outcome (responder versus tolerant/resistant) when needed to improve model stability. The binary logistic regression models were fitted with mechanistic markers (RNS, ROS, *CAT1*, *GPX1*, and *SOD1*) as predictors, with optional adjustment for species and treatment where specified. The model fit was evaluated using standard goodness-of-fit outputs, and effects were reported as odds ratios with 95% confidence intervals. In addition, decision-tree classification (classification and regression tree; CRT) was used as an interpretable ML-style approach to derive rule-based predictors of the responder group from mechanistic variables. Ten-fold cross-validation was applied to estimate the generalization error, and terminal node membership was subsequently cross-tabulated against treatment to identify treatments enriched for responder or tolerant/resistant-associated mechanistic rules.

Finally, to visualize the global mode-of-action structure, a principal component analysis (PCA) was performed on treatment × species mechanistic signatures (means) across ROS, RNS, *CAT1*, *GPX1*, and *SOD1*. Sampling adequacy was evaluated using the Kaiser–Meyer–Olkin measure and Bartlett’s test of sphericity. Components were extracted using principal components with Varimax rotation, and two-component solutions were retained for visualization based on eigenvalues and explained variance. Rotated loadings were used to interpret mechanistic axes, and rotated component scores (PC1/PC2) were used to generate a treatment landscape plot; treatment centroids across species were computed as mean PC scores to summarize formulation-level positioning in mechanistic space. For visualization clarity, plots were generated both with and without the oxidative-stress control anchor where appropriate [47,48,49,50,51].

## 3. Results

### 3.1. Chemical Characterization of Lemon Balm Essential Oil (L)

The HPLC/HRMS analysis of the lemon balm essential oil enabled the preliminary characterization of citral-related constituents. The sample and citral reference standard exhibited closely matching retention times for the two isomeric peaks at approximately 9.87 and 10.10 min, supporting their assignment as neral (β-citral) and geranial (α-citral). Detection was achieved under UHPLC-Orbitrap HRMS conditions using positive HESI ionization and a water/acetonitrile–formic acid gradient, allowing accurate mass measurement and MS/MS-based confirmation of these features. Accordingly, the present data should be interpreted as a reliable preliminary compositional assessment confirming the presence and dominance of citral isomers, with further method optimization required for comprehensive profiling. Detailed chromatographic and spectral data are provided in the Supplementary Materials (Figures S14–S17 and Table S12).

### 3.2. Species-Level Susceptibility, Phase Plasticity, and Survival Effects

**Figure 1:**
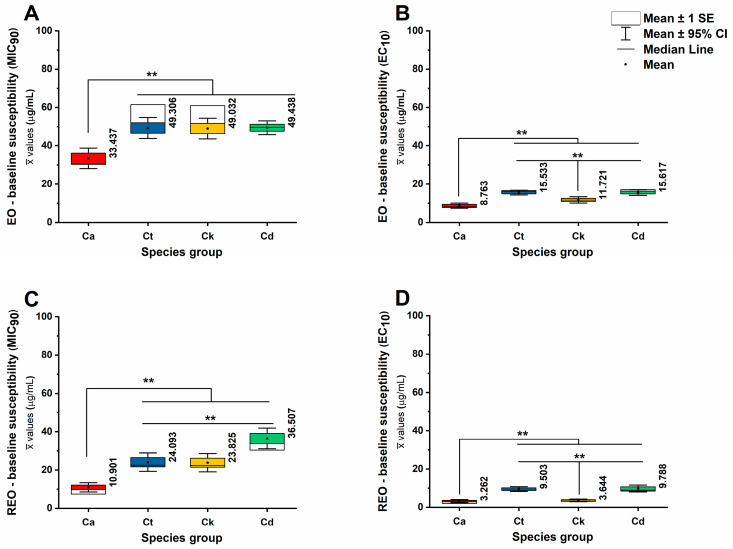
Species-level susceptibility thresholds (MIC_90_ and EC_10_) across Candida species for native EO and RAMEB-EO formulations. (**A**) MIC_90_ and (**B**) EC_10_ values for the native EO condition; (**C**) MIC_90_ and (**D**) EC_10_ values for the corresponding RAMEB-EO (REO) condition, shown across the four species groups: Ca (red: *C. albicans*), Ct (blue: *C. tropicalis*), Ck (yellow: *C. krusei*), and Cd (green: *C. dubliniensis*). Boxplots summarize the distribution within each species (median line with interquartile range), with mean-based overlays shown as indicated in the legend (mean marker; mean ± 1 SE; mean ± 95% CI). Horizontal brackets denote statistically significant between-species differences for the given endpoint/formulation (multiple-comparison-adjusted pairwise contrasts); significance levels are indicated as ** (*p* < 0.001) on the plot. Error bar overlays represent mean ± SE and mean ± 95% CI as indicated.

To define baseline interspecies susceptibility architecture, we quantified the growth-inhibitory (MIC_90_) and sub-inhibitory (EC_10_) concentration thresholds for native essential oils and their corresponding RAMEB inclusion complexes across *C. albicans*, *C. tropicalis*, *C. krusei*, and *C. dubliniensis*. The MIC_90_ values reflect near-complete inhibition following the prolonged exposure of early-phase cells, whereas EC_10_ values capture early functional suppression under short-term exposure in mid-log-phase populations. These parameters therefore interrogate distinct physiological states and exposure kinetics. Comparative evaluation across species enables discrimination between formulation-dependent potency shifts and intrinsic taxon-specific resistance characteristics. The distributional structure of MIC_90_ and EC_10_ values is summarized in Figure 1 and Supplementary Tables S3 and S4 (MIC_90_ and EC_10_ raw data, respectively).

To characterize species-dependent susceptibility patterns and quantify the separation between sublethal and inhibitory concentration ranges, raw MIC_90_ and EC_10_ values were compared across the four Candida species using nonparametric methods, as expected for concentration–response endpoints with non-normal distributions. Analyses were performed on the experimental essential oil/RAMEB treatment set (controls excluded), with N representing experimental repeat measurements contributing to each species distribution (total N = 162 repeats; *C. albicans n* = 54, *C. tropicalis n* = 36, *C. krusei n* = 36, *C. dubliniensis n* = 36).

Species differed significantly in MIC_90_, indicating that the growth-inhibitory threshold is strongly species-structured (Kruskal–Wallis H(3) = 193.329, *p* < 0.001). Mean MIC_90_ values (weighted average) were lowest for *C. albicans* (22.186 ± 1.005 µg/mL) and increased markedly for non-albicans species, including *C. tropicalis* (36.557 ± 1.273 µg/mL), *C. krusei* (36.371 ± 1.257 µg/mL), and most prominently *C. dubliniensis* (42.789 ± 1.003 µg/mL) for both the EO and REO groups. Consistent with these medians, mean rank ordering placed *C. dubliniensis* highest and *C. albicans* lowest, supporting a shift toward reduced inhibitory susceptibility in the non-albicans species (*p* < 0.001).

The MIC_90_ values demonstrated clear species-specific structuring and varied significantly across the eight experimental treatments (L, B, P, T, RL, RB, RP, and RT) (see Table S2 for strain vs. treatment-wise comparisons). The treatment potency of RT (*C. albicans*: 2.785 ± 0.179 µg/mL), (*C. krusei*: 9.168 ± 0.88 µg/mL), (*C. tropicalis*: 9.902 ± 1.02 µg/mL), (*C. dubliniensis*: 16.44 ± 0.343 µg/mL) and RB (*C. albicans*: 5.812 ± 0.376 µg/mL), (*C. tropicalis*: 18.719 ± 1.811 µg/mL), (*C. krusei*: 19.25 ± 1.977 µg/mL), and (*C. dubliniensis*: 27.357 ± 0.571 µg/mL) emerged as the most potent inhibitory conditions across all taxa. *C. albicans* exhibited the highest susceptibility in these groups (*p* < 0.001). Conversely, the highest inhibitory thresholds were observed in treatments L, P, and RP. Notably, *C. dubliniensis* showed a distinct resistance profile under treatment RP (65.559 ± 0.454 µg/mL), while *C. tropicalis* and *C. krusei* displayed their maximum MIC_90_ values under treatment L (64.16 ± 0.483 µg/mL, and 62.656 ± 0.32 µg/mL, respectively). Across nearly all tested conditions, *C. albicans* remained the most sensitive species (*p* < 0.001). Non-albicans species (NACs) required consistently higher concentrations for inhibition, with *C. tropicalis* and *C. krusei* showing nearly identical resistance patterns in several treatments, such as P (62.447 ± 0.325 µg/mL and 62.076 ± 0.623 µg/mL) and RL (22.395 ± 1.356 µg/mL, and 23.134 ± 1.33 µg/mL), respectively. Treatments T, B, and RL resulted in intermediate MIC_90_ values, further emphasizing a gradient of susceptibility that is both treatment-dependent and species-specific. The reference antifungals demonstrated species-dependent susceptibility patterns consistent with established profiles. The AM showed uniformly low MIC_90_ values across all species, whereas FL exhibited reduced efficacy against *C. krusei*, consistent with its intrinsic resistance. These controls served as functional anchors to validate assay performance and contextualize the activity of EO and RAMEB-EO formulations.

A similarly strong species effect was observed for EC_10_ (Kruskal–Wallis H(3) = 103.63, *p* < 0.001), demonstrating that sensitivity within the sublethal concentration range also varies by species. The EC_10_ was lowest for *C. albicans* (overall distribution of 6.026 ± 0.254 µg/mL) and increased in *C. krusei* (7.679 ± 0.35 µg/mL) but was substantially higher in *C. tropicalis* (12.524 ± 0.307 µg/mL) and *C. dubliniensis* (12.772 ± 0.375 µg/mL). The rank pattern for EC_10_ placed *C. tropicalis* and *C. dubliniensis* at the highest mean ranks, suggesting that these species require higher concentrations to elicit early measurable inhibition, consistent with diminished low-dose sensitivity or enhanced tolerance-like behavior under sub-inhibitory exposure (*p* < 0.001).

The analysis of EC_10_ values revealed a distinct species-specific structuring of sublethal thresholds across the eight treatment conditions (Table S4 for strain vs. treatment-wise comparisons). *C. albicans* consistently emerged as the most sensitive species, particularly under treatments RT (0.731 ± 0.507 µg/mL) and RB (1.652 ± 0.103 µg/mL), where the onset of inhibitory effects occurred at the lowest concentrations. In contrast, non-albicans species required significantly higher concentrations to reach ~10% effective threshold for RT (2.867 ± 1.7553 µg/mL) and RB (6.2118 ± 3.308 µg/mL). While RT remained the most potent treatment across all taxa, maintaining mean values under ~10 µg/mL for both *C. tropicalis* and *C. krusei* treatments, L (17.905 ± 0.484 µg/mL, and 16.655 ± 0.409 µg/mL) and P (16.87 ± 0.093 µg/mL, and 17.139 ± 0.324 µg/mL) demonstrated the highest sublethal thresholds. Specifically, *C. tropicalis* and *C. krusei* exhibited peak EC_10_ values under treatment L, while *C. dubliniensis* reached its maximum threshold under treatment RP (19.276 ± 0.264 µg/mL). Overall, these data clearly demonstrate that species-specific differences are most evident at the sublethal level, with non-albicans species displaying a broader range of resistance before the initiation of inhibitory activity at their mid-log phase when compared to their early growth phase. Detailed MIC_90_ and EC_10_ values for reference antifungals are provided in Supplementary Tables S3 and S4 respectively.

**Figure 2:**
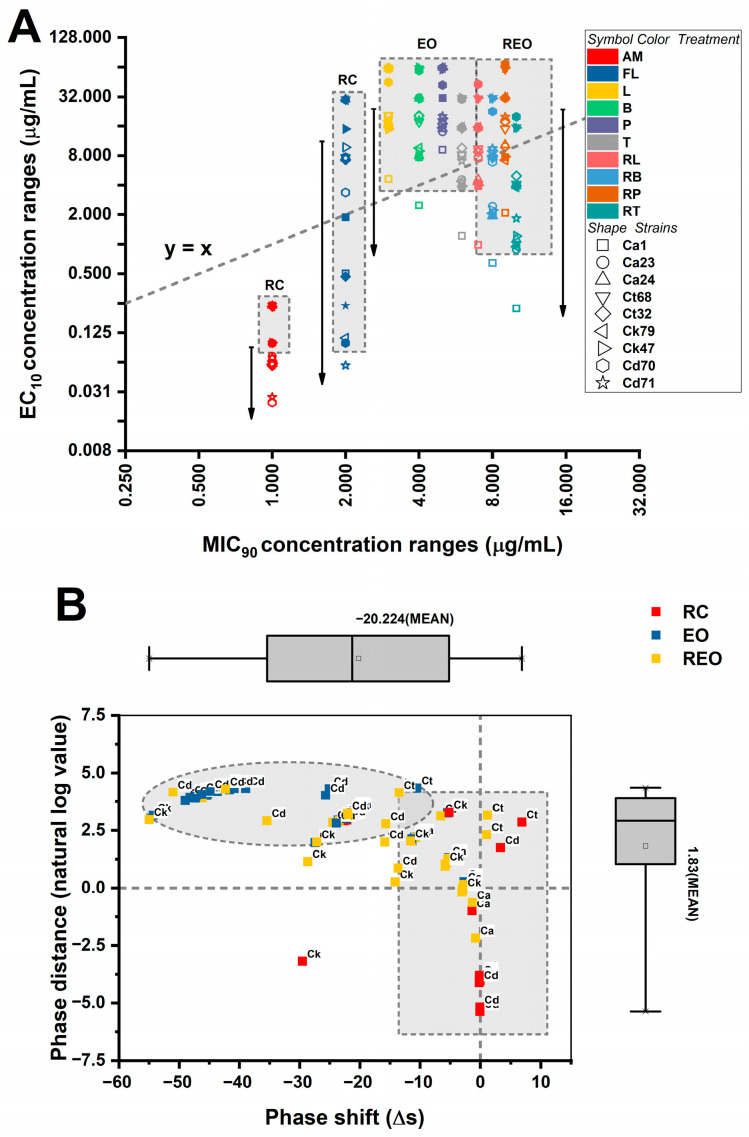
The MIC_90_-EC_10_ phase plasticity landscape highlights species- and formulation-dependent separation between inhibitory and sub-inhibitory regimes. (**A**) Two-dimensional susceptibility map plotting MIC_90_ (*y*-axis) against EC_10_ (*x*-axis) for all strain–treatment observations. Points are colored by treatment (see legend) and grouped by species (Ca, Ct, Ck, and Cd). Gray dashed guides/boxes indicate the within-species spread of MIC_90_-EC_10_ coordinates, emphasizing the differences in susceptibility dispersion (plasticity) among species. (**B**) Distribution and geometry of the phase plasticity window, defined as the separation between EC_10_ and MIC_90_. The boxplot summarizes the overall distribution of MIC_90_-EC_10_ separation (Δs = EC_10_ − MIC_90_; top), and the scatter shows MIC_90_ vs. EC_10_ colored by formulation class (RC: antifungal reference controls vs. native EO vs. REO: RAMEB-EO), illustrating how formulation shifts points within the MIC-EC plane. Marginal boxplots (right/top) summarize axis-wise distributions. A larger EC_10_-MIC_90_ separation indicates a broader sub-inhibitory window (greater phase plasticity), whereas tighter coupling suggests a narrower transition from partial effect to near-complete inhibition.

Because absolute inhibitory thresholds do not fully describe the dynamic transition between sublethal and inhibitory exposure regimes, the geometric relationship between EC_10_ and MIC_90_ values for each strain–treatment combination were furthermore analyzed. The resulting Δs (EC_10_-MIC_90_) and EC_10_/MIC_90_ metrics quantify the separation between early measurable growth perturbation and near-complete growth inhibition, thereby operationalizing the concept of “phase plasticity”. This framework allows the assessment of whether species differ in the breadth of their sub-inhibitory tolerance window and whether RAMEB complexation alters the positioning of treatments within the MIC-EC susceptibility landscape. The multidimensional plasticity architecture is depicted in Figure 2.

To capture how the sublethal response region relates to the inhibitory threshold, two plasticity metrics were derived: phase shift (Δs = EC_10_ − MIC_90_ (absolute separation)) and phase distance = EC_10_/MIC_90_ (relative separation). Both metrics differed significantly across species (phase shift: Kruskal–Wallis H(3) = 189.555, *p* < 0.001; phase distance: H(3) = 232.148, *p* < 0.001), indicating that EC_10_ is positioned differently relative to MIC_90_ depending on the species. For phase shift, means were negative across all species, consistent with EC_10_ occurring at concentrations below the MIC_90_ threshold. However, the magnitude of this separation differed: *C. albicans* showed the smallest absolute separation (−16.159 ± 0.757), whereas *C. tropicalis* (−24.033 ± 1.061) and especially *C. krusei* (−28.691 ± 0.982) and *C. dubliniensis* (−30.066 ± 0.693) exhibited more negative shifts. Interpreted biologically, a more negative phase shift indicates a larger gap between the concentration where early inhibition begins and the concentration required for near-complete growth inhibition, suggesting a species-dependent breadth of the sublethal “adaptive window”. For phase distance, species differences were also evident. *C. krusei* exhibited the lowest EC_10_/MIC_90_ ratio (0.209 ± 0.004), consistent with the largest proportional separation between sublethal and inhibitory thresholds. In contrast, ratios were higher for *C. albicans* (0.281 ± 0.001), *C. dubliniensis* (0.285 ± 0.004), and *C. tropicalis* (0.473 ± 0.018). Taken together, these plasticity analyses indicate that non-albicans Candida species, particularly *C. krusei* and *C. dubliniensis*, exhibit a broader separation between EC_10_ and MIC_90_, supporting the interpretation that adaptive/tolerance-linked concentration ranges may differ by species (*p* < 0.001).

**Figure 3:**
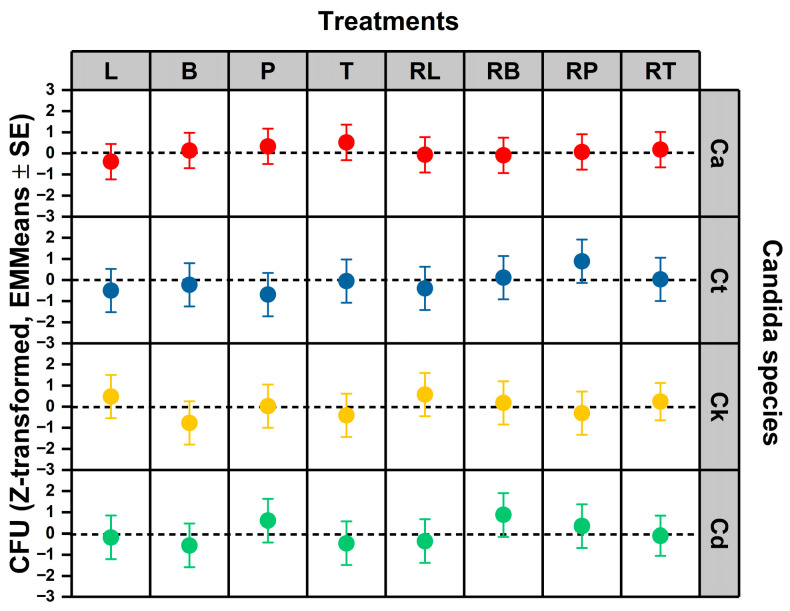
Species-stratified survival (CFU) responses to essential oils and RAMEB–EO complexes. Estimated marginal means (EMMeans ± SE) of CFU (species-wise Z-transformed) from linear mixed models are shown for each treatment (L, B, P, T and their RAMEB-complexed counterparts RL, RB, RP, and RT). Rows correspond to Candida species (red: Ca; blue: Ct; yellow: Ck; and green: Cd). The horizontal dashed line indicates the within-species reference level (Z = 0). Negative EMMeans reflect reduced CFUs (greater survival suppression) relative to the species baseline, whereas positive values indicate comparatively higher CFU values. This visualization emphasizes species dependence and formulation-dependent shifts (EO vs. RAMEB–EO) in survival outcomes across the treatment panel (non-significant data). Error bars represent ± standard error (SE) of the estimated marginal means.

To determine whether species- and formulation-dependent differences in concentration thresholds translate into functional survival outcomes under working exposure conditions, colony-forming unit (CFU) dynamics were analyzed using species-wise standardized linear mixed models. Z-transformation within species isolates treatment-driven deviations from baseline survival variability and permits the direct comparison of EO and RAMEB–EO effects across taxa. This analysis tests whether the susceptibility and plasticity differences observed at the concentration–response level manifest as measurable shifts in viable population burden. Species-stratified estimated marginal means of standardized CFU responses are presented in Figure 3.

Survival was quantified by calculating the area under the curve (AUC) from CFU counts across all time points. To account for inherent growth variations between strains, these AUC values were Z-transformed. This standardization ensures that values represent within-strain deviations from the baseline, focusing the analysis on relative survival rather than absolute differences in growth capacity. In linear mixed models accounting for experimental repeats, there was no evidence that standardized CFU counts differed by treatment, species, or their interaction within the experimental EO/RAMEB set (treatment: F(7, 40.538) = 0.744, *p* = 0.637; species class: F(3, 41.579) = 0.223, *p* = 0.880; treatment × species class: F(21, 41.918) = 0.891, *p* = 0.602). The EMMeans therefore indicated only directional trends (e.g., more negative values suggesting stronger CFU suppression), but these differences were not statistically robust under the model. Although susceptibility thresholds (MIC_90_ and EC_10_) and phase plasticity exhibited clear species-dependent structuring, the standardized CFU responses did not differ significantly across species or treatments. This suggests that, under normalized conditions, survival outcomes are comparatively conserved despite upstream differences in concentration–response behavior. Susceptibility and plasticity analyses demonstrated clear species-dependent variations at the concentration–response level. However, under the specific working concentrations utilized, survival measured via CFU remained consistent (non-significant) across all tested Candida strains (Figure 3). As no explicit exposure-termination step was employed prior to CFU enumeration, a minor contribution of treatment carryover during plating cannot be excluded. However, serial dilution substantially reduces residual concentrations, and therefore the observed effects are interpreted as reflecting acute exposure responses.

### 3.3. Treatment-Induced Oxidative and Nitrosative Stress Responses to Stratify Exposure Signatures

To determine whether the essential oil and RAMEB-EO formulations elicit distinct oxidative and nitrosative stress programs across Candida species, standardized mechanistic markers encompassing intracellular reactive oxygen species (ROS), reactive nitrogen species (RNS), and key antioxidant response genes (*CAT1*, *GPX1*, and *SOD1*) were analyzed. Because these variables were Z-transformed within species, the resulting profiles reflect treatment-induced deviations from each species’ baseline stress architecture rather than absolute interspecies differences. This approach was used to enable the direct comparison of mechanistic exposure signatures across taxa while controlling for inherent species-level variability in redox homeostasis.

**Figure 4:**
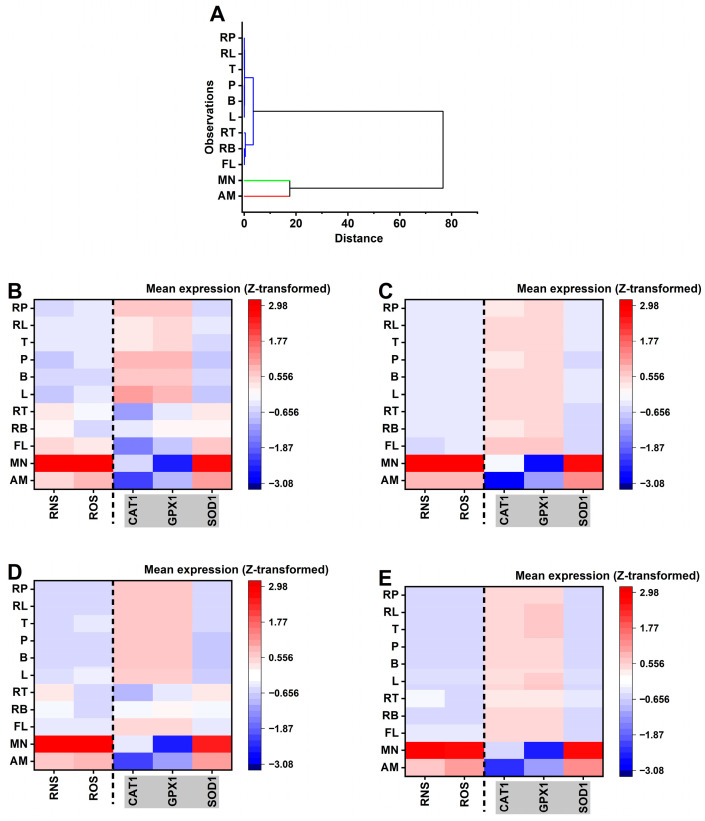
Treatment-induced oxidative/nitrosative stress signatures across Candida species. (**A**) Global hierarchical clustering (Euclidean distance) of treatment signatures based on the standardized mechanistic marker panel RNS, ROS, *CAT1*, *GPX1*, and *SOD1* (species-wise Z-transformed). (**B**–**E**) Species-stratified heatmaps showing mean Z-scores for each marker by treatment in (**B**) *C. albicans* (Ca), (**C**) *C. tropicalis* (Ct), (**D**) *C. krusei* (Ck), and (**E**) *C. dubliniensis* (Cd). The rows denote treatments (AM and FL: antifungal reference controls; MN: oxidative-stress control; L/B/P/T: essential oils; and RL/RB/RP/RT: corresponding RAMEB inclusion complexes). The columns indicate mechanistic readouts; the dashed vertical line separates stress markers (RNS/ROS) from antioxidant gene responses (*CAT1*/*GPX1*/*SOD1*). The color scale represents mean Z-transformed deviation within species (red = higher-than-species mean; blue = lower-than-species mean), enabling the direct comparison of mechanistic exposure signatures across treatments while controlling baseline species differences (see Supplementary Heatmap Figure S1 for treatment-associated changes in antioxidant gene programs). The shaded grey region identifies the specific genes analyzed in this study.

Hierarchical clustering was first applied to the aggregated treatment signatures to define global mechanistic structure. Species-stratified heatmaps were then constructed to visualize how each treatment perturbs the balance between stress generation (ROS/RNS) and antioxidant compensation (*CAT1*/*GPX1*/*SOD1*). Together, these analyses interrogate whether formulation effects primarily alter oxidative burden, antioxidant engagement, or the coordination between these systems. The resulting mechanistic landscape and oxidative–nitrosative response cluster classification is presented in Figure 4 and Table 1 respectively.

A visual inspection of the species-specific heatmaps (Figure 4: panel B–E) for *C. albicans*, *C. tropicalis*, *C. krusei*, and *C. dubliniensis* demonstrates that these oxidative–nitrosative exposure signatures are broadly conserved across the tested taxa. Despite the significant differences observed in the absolute concentration thresholds (EC_10_ values), the relative mechanistic deviations—most notably the high-stress profile of MN and the suppressed response of AM—remain consistent across all species. This conservation suggests that while species differ in their individual sensitivity thresholds, the underlying cellular “mode of action” for these treatments is largely species-independent. Hierarchical clustering (Figure 4A) confirms a clear binary split between high-stress anchors and the experimental treatment set. The experimental treatments, B (96.84 ± 6.535, 100.499 ± 0.744), L (95.042 ± 1.458, 100.399 ± 1.094), P (95.146 ± 1.287, 100.042 ± 0.432), T (101.031 ± 100.28 ± 1.737), RL (100.974 ± 15.738, 100.194 ± 1.235), RB (110.951 ± 20.18, 99.72 ± 1.2), and RP (97.997 ± 7.6, 100.907 ± 2.1), for increased RNS/ROS generation compared to the UC formed a tightly knit hierarchical group characterized by low ROS/RNS generation and a coordinated *CAT1*/*GPX1*-driven adaptive response. The proximity of RT and RB to the reference antifungal FL in the dendrogram further suggests that these specific combinations represent a transition state toward higher inhibitory activity while maintaining the general mechanistic signature of the experimental group (see Supplementary Tables S5 and S8 for raw percentage reactive oxygen–nitrogen species generation compared to the untreated control and pairwise dendrogram distance matrix for hierarchical clustering, respectively).

To define treatment-specific oxidative–nitrosative stress exposure signatures and the associated antioxidant response programs, standardized mechanistic endpoints (ROS, RNS, *CAT1*, *GPX1*, and *SOD1*) across *C. albicans*, *C. tropicalis*, *C. krusei*, and *C. dubliniensis* we analyzed by k-clustering (Table 1). Because all mechanistic variables were Z-transformed within species (split by species standardization), this analysis emphasizes relative mechanistic deviations (mode-of-action patterns) rather than absolute potency or concentration effects. Accordingly, the experimental treatments were interpreted together with reference anchors: AM and FL as antifungal controls and MN as an oxidative stress positive control. Mechanistic profiles were summarized at the treatment × species level (mean Z-values), yielding N = 44 exposure signatures corresponding to 11 treatments measured in each of four species (11 signatures per species). The treatments included A, F, L, B, P, T, RL, RB, RP, RT, and MN. The k-means clustering (k = 3) was applied to the five-dimensional signature space (ROS, RNS, *CAT1*, *GPX1*, and *SOD1*) and resolved three mechanistic classes (cluster 1 n = 4, cluster 2 n = 7, cluster 3 n = 33). Cluster centers (Z units) indicated that class separation was driven by both the magnitude of ROS/RNS generation and the balance of antioxidant response gene shifts.

In Table 1, cluster 1 exhibited the strongest stress phenotype, with markedly elevated RNS (+2.904) and ROS (+2.839). This was accompanied by a strong *SOD1* upshift (+2.616) and pronounced *GPX1* suppression (−2.6), while *CAT1* remained near baseline (−0.372). Importantly, cluster 1 consisted exclusively of MN (4/4 signatures), indicating that MN produced a conserved high-stress signature across all four species and served as a robust “high oxidative–nitrosative burden” anchor in the mechanistic space.

Cluster 2 showed modest elevations in stress markers (RNS +0.484; ROS +0.443) but a disproportionately reduced antioxidant response, particularly *CAT1* (−1.88) and *GPX1* (−0.832), with only a modest positive shift in *SOD1* (+0.839). In terms of composition, this mechanistic class was dominated by AM (4/7 signatures; one per species), with additional membership from RT (2 signatures) and FL (1 signature). Thus, AM displayed a reproducible cross-species signature consistent with stress present but *CAT1*/*GPX1* response suppressed, whereas FL and RT showed an occasional drift into this phenotype. Cluster 3 formed the dominant mechanistic class and was characterized by below-baseline ROS/RNS (RNS −0.454; ROS −0.449) together with relative increases in *CAT1* (+0.469) and *GPX1* (+0.507) and a decrease in *SOD1* (−0.51). This profile is consistent with an adaptive/low stress signature in which oxidative/nitrosative stress markers are not elevated and antioxidant response patterns preferentially involve *CAT1*/*GPX1* rather than *SOD1*. Cluster 3 included all experimental treatments across all species (B, L, P, T, RL, RB, and RP each contributed 4/4 species signatures) and contained the most signatures for F (3/4) and RT (2/4). The distances between the cluster centers supported clear separation among the mechanistic classes, particularly between the MN-driven high-stress class and the dominant low-stress/adaptive class (cluster 1 vs. 3 distance = 6.498; cluster 1 vs. 2 = 4.490; cluster 2 vs. 3 = 3.287). Despite distinct mechanistic classes, cluster membership was not associated with species under the species-wise standardized framework. Pearson’s chi-square test showed no enrichment (χ^2^(6) = 1.905, *p* = 0.928), indicating that the clustering structure is primarily treatment-signature driven rather than species-driven in this mechanistic space. This suggests that the oxidative–nitrosative exposure signatures are broadly conserved across *C. albicans*, *C. tropicalis*, *C. krusei*, and *C. dubliniensis*, with species effects more likely to emerge in downstream phenotype/efficacy endpoints than in the top-level mechanistic stratification. Within the mechanistic framework (species-wise Z-standardized ROS/RNS and antioxidant gene responses), the most favorable exposure signature was the dominant cluster 3 pattern—low ROS/RNS with *CAT1*/*GPX1* upshift—capturing all experimental treatments (B, L, P, T, RL, RB, and RP) consistently across species and the most signatures for FL and RT. In contrast, A reproducibly mapped to cluster 2, characterized by moderate ROS/RNS elevation coupled to suppressed *CAT1*/*GPX1* response, representing a mechanistically less desirable pattern in this signature space. As expected, MN formed cluster 1 exclusively, producing the strongest oxidative–nitrosative burden and serving as the high-stress positive control anchor. Overall, these results indicate that the experimental treatment set predominantly expresses a mechanistic profile consistent with low oxidative/nitrosative burden and adaptive antioxidant engagement, whereas AM is associated with a stress-plus-suppressed-response signature and MN represents maximal stress induction.

### 3.4. Convergence of Planktonic Efficacy and Mature Biofilm Eradication Across Candida Species

**Figure 5:**
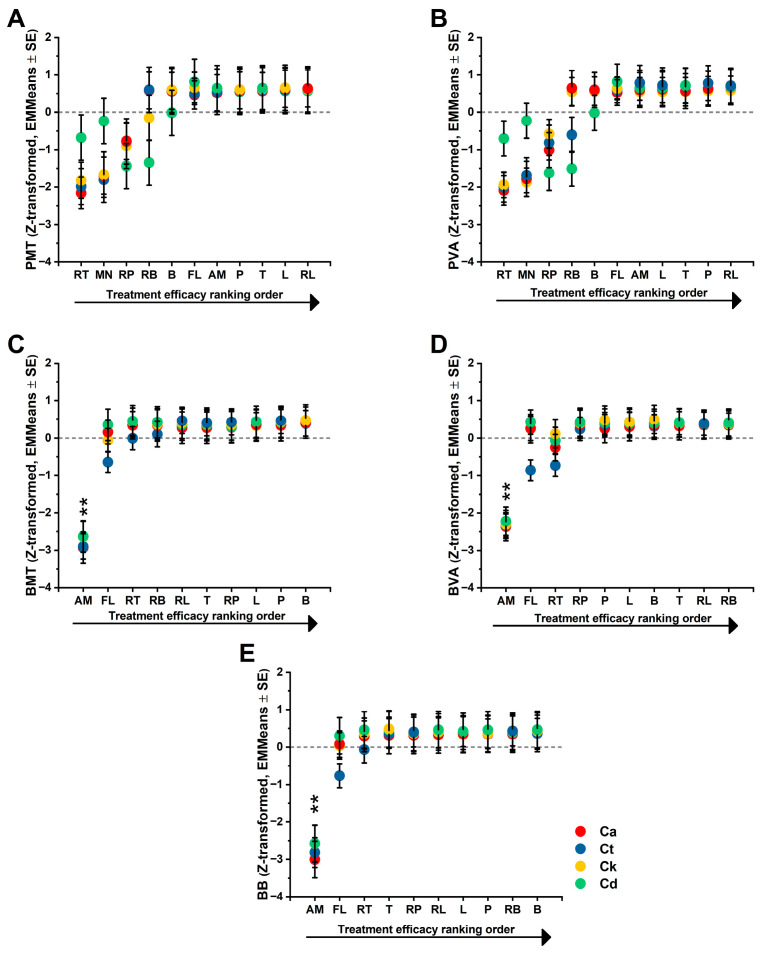
Planktonic metabolism and viability reveal treatment efficacy and species dependence. Panels show estimated marginal means (EMMeans ± SE) from linear mixed models (REML; Satterthwaite degree of freedom) for planktonic functional endpoints across the experimental treatment set (L, B, P, T, RL, RB, RP, and RT), with species indicated by color (red: Ca; blue: Ct; yellow: Ck; and green: Cd). The *y*-axis is the species-wise Z-transformed response (negative values indicate a shift below the species mean; positive values indicate a shift above the species mean). The *x*-axis lists treatments, grouped by parent essential oil (EO) and its corresponding RAMEB inclusion complex (REO). (**A**–**E**) Endpoint-specific EMMeans profiles (one endpoint per panel) illustrate the consistent treatment-driven modulation of planktonic physiology, while differences in the separation and ordering of species-colored points across the treatments indicate species-dependent efficacy patterns (treatment × species effects). The treatment efficiency levels for the experimental parameters (panel (**A**): planktonic metabolic activity/PMT; panel (**B**): planktonic viability/PVA; panel (**C**): biofilm attached cellular metabolic activity/BMT; panel (**D**): biofilm attached cellular viability/BVA; and panel (**E**): biofilm biomass/BB) decline along the horizontal axis, as indicated by the arrow. The significance levels are indicated as ** (*p* < 0.001) on the plot. Treatment efficacy for each experimental parameter was inferred from the direction and magnitude of the standardized response associated with each endpoint.

To determine whether early planktonic inhibitory effects translate into the suppression of structured biofilm-associated phenotypes, we next evaluated treatment performance across complementary functional endpoints spanning planktonic metabolism (PMT), planktonic viability (PVA), biofilm-associated metabolic activity (BMT), biofilm-associated viability (BVA), and biofilm biomass (BB). Representative bright-field microscopic images of crystal violet stained biofilms are provided in the Supplementary Materials (Figures S4–S13), illustrating treatment-dependent differences in biofilm coverage and structural organization consistent with the quantitative biomass measurements. Linear mixed models were applied to species-wise Z-transformed responses to enable the comparison of relative within-species shifts across treatments. This integrative framework allowed the direct assessment of whether treatments that perform strongly under planktonic conditions retain inhibitory hierarchy in the more structured and stress-resilient biofilm context, and whether such convergence is conserved across the experimented Candida species. The resulting estimated marginal means profiles are shown in Figure 5 (see Supplementary Tables S6 and S7 for reduced percentage PMT, PVA, BB, BMT and BVA raw data).

The results summarized in Figure 5 are supported by two primary visual analyses. First, the estimated marginal means plot (EMMeans) illustrates the overall ranking of treatments, highlighting the significant suppression of metabolism and viability by RT and RP relative to the neutral baseline of the remaining panel. Within each panel, treatments are ordered along the horizontal axis according to their estimated marginal means (EMMeans) for the respective endpoint, such that the ordering reflects endpoint-specific efficacy gradients. This ranking is derived independently for each endpoint and should not be interpreted as a global efficacy ranking. Second, the treatment × species interaction plot visualizes the biological basis of the statistical interaction; it specifically highlights the crossover effects where RB and RP achieve maximal efficacy in *C. dubliniensis* compared to their more moderate profiles in *C. albicans* and *C. tropicalis*. These visualizations confirm the sensitivity of the Z-standardized scale in detecting species-specific physiological shifts.

Strong treatment effects were observed for both planktonic endpoints, demonstrating that Candida planktonic physiology was substantially modulated by the experimental treatment panel. For metabolism, a highly significant overall treatment effect was found (PMT: F(7, 41.506) = 42.829, *p* < 0.001), and this effect was even more pronounced for viability (PVA: F(7, 39.861) = 88.926, *p* < 0.001), indicating that viability provided high sensitivity for treatment discrimination. The species’ main effect was not found to be significant for PMT (*p* = 0.3) and was borderline for PVA (*p* = 0.051). Given the species-wise standardization, these results were expected and indicated that the dominant signal was treatment-driven rather than a reflection of baseline between-species offsets. A significant treatment × species interaction was exhibited by both endpoints, demonstrating that treatment efficacy was not uniform across the four species. This was statistically confirmed for both PMT (F(21, 43.84) = 2.765, *p* = 0.00224) and PVA (F(21, 41.521) = 6.42, *p* < 0.001), while simple-effect analyses confirmed that treatment differences remained significant within each species for both endpoints (all *p* < 0.001), supporting a species-stratified interpretation. A concise ranking of treatments on the standardized scale was provided by estimated marginal means (EMMeans), showing consistency between metabolic and viability suppression as efficacy indicators. RT was identified as the most inhibitory treatment, showing the strongest suppression of metabolism (EMMeans: −1.659 ± 0.133) and viability (−1.679 ± 0.096), while RP (metabolic activity: −0.971 ± 0.134, viability: −1.004 ± 0.096) was ranked second and remained significantly more inhibitory than most of the panels. Whereas RB (0.078 ± 0.134) was found to be near neutral for metabolism but showed a modest, significant reduction in viability (−0.229 ± 0.096), positive EMMeans were displayed by L, B, P, T, and RL, reflecting weaker inhibition compared to RT/RP. The dominance of RT and RP was reinforced by Bonferroni-adjusted pairwise comparisons, which identified RT as significantly more inhibitory than all other treatments. The biological basis of the interaction was revealed by an inspection of the EMMeans within each species, where RT was characterized as a broad-spectrum inhibitor with strongly negative EMMeans produced in all four species for both endpoints, although the magnitude of suppression was attenuated in *C. dubliniensis* (*p* < 0.001). A particularly pronounced inhibitory profile was shown by RP in *C. dubliniensis* (PMT: −1.438 ± 0.279; PVA: −1.621 ± 0.201), suggesting specific effectiveness against this species, while a striking species-dependent pattern was shown by RB, which explains a major portion of the interaction by showing pronounced inhibition in *C. dubliniensis* (PVA: −1.509 ± 0.203) despite not being consistently inhibitory in *C. albicans* and *C. tropicalis*.

This physiological modulation extended to the inhibition of premature biofilms, where efficacy was similarly quantified through biofilm biomass (BB), metabolic activity (BMT), and viability (BVA). For biofilm metabolism, no significant main effect was detected for treatment (F(7, 40.226) = 0.572, *p* = 0.774) or species (F(3, 41.071) = 0.893, *p* = 0.453), indicating that no single treatment performed as the “best” across all species. Global EMMeans trends for metabolism showed RT at the lowest level (0.306; 95% CI: 0.183–0.429) and RL at the highest (0.377; 95% CI: 0.254–0.501). Analyzing the reduced metabolic activity (compared to the UC), a shift from the essential oil to the RAMEB-complexed form, T → RT, showed a non-significant metabolic shift (0.049; 95% CI −0.24–0.338). Overall, there was no globally significant difference observed among the treatments, as the collective mean for metabolic inhibition (BMT) for all tested essential oils and their RAMEB-complexed counterparts remained consistently within the 50–60% range relative to the untreated control (UC) (*p* < 0.001).

Biofilm viability was identified as the most decisive and treatment-dependent readout in the premature biofilm model (F(7, 39.997) = 10.258, *p* < 0.001). A non-significant treatment × species interaction was present (F(21, 40.674) = 1.037, *p* = 0.446), and the species effect remained borderline non-significant (F(3, 40.625) = 2.664, *p* = 0.446). RT produced the lowest overall viability (EMMeans: −0.232; 95% CI: −0.369 to −0.096), while B showed the highest (0.392; 95% CI: 0.255 to 0.529). The T → RT transition provided a very large improvement in suppression (shift: 0.62; 95% CI: 0.3 to 0.939, *p* < 0.001), marking the strongest “RAMEB-benefit” signal. In contrast to the physiological markers, premature biofilm biomass appeared robust and was not detectably altered by treatments or formulation status on the Z-standardized scale. No significant effects were detected for the treatment (F(7, 40.334) = 0.356, *p* = 0.922), the species (F(3, 41.474) = 1.054, *p* = 0.379), or the interaction (F(21, 41.564) = 0.516, *p* = 0.947). Overall, EMMeans trends showed RT with the lowest biomass (0.268; 95% CI: 0.114 to 0.423) and RB with the highest (0.398; 95% CI: 0.243 to 0.552), though these differences were not statistically significant. This highlights a critical decoupling in early biofilm inhibition: while biological markers like viability and metabolism are highly responsive to specialized treatments, the physical biofilm biomass remains insensitive at this stage. In conclusion, treatment efficacy is readily detected at multiple levels and is characterized by a clear species dependence. The identification of broad-spectrum (RT) versus species-selective (RB and RP) patterns, where RAMEB complexation is not uniformly beneficial, successfully informs subsequent biofilm maturation and mechanistic integration analyses.

### 3.5. Integrated Treatment Ranking Identifies the Most Effective Regimens Across Endpoints

**Figure 6:**
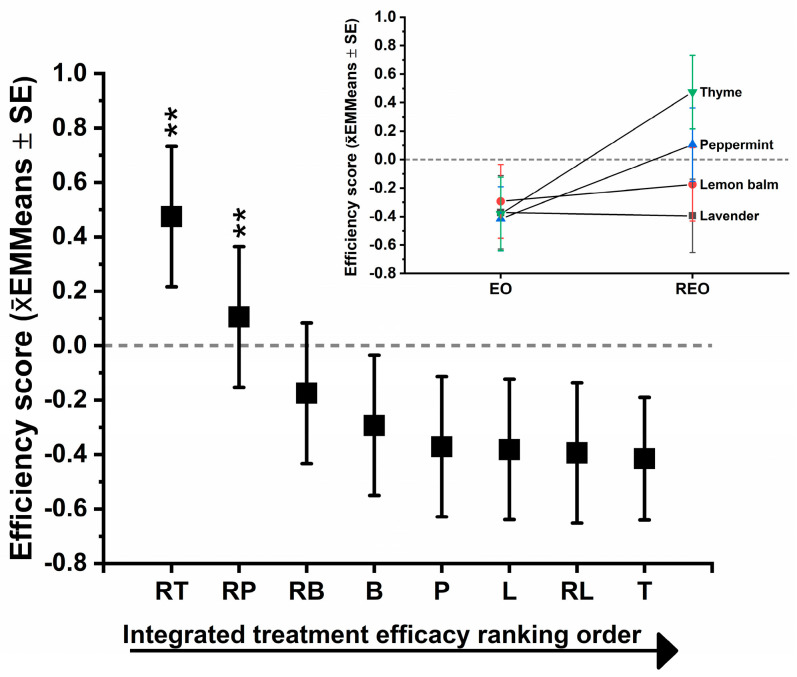
Integrated treatment ranking identifies the most effective regimens across endpoints and highlights EO→RAMEB formulation shifts. The main panel shows treatment efficacy ranking based on the composite efficacy score (estimated marginal means, EMMeans ± SE) derived from linear mixed modeling on the standardized (species-wise Z-transformed) integrated endpoint score. Treatments are ordered left-to-right by increasing overall efficacy (arrow indicates rank direction). The dashed horizontal line denotes the neutral reference (0; no shift from the species-wise mean); more positive values indicate higher composite efficacy on the standardized scale, whereas negative values indicate comparatively weaker efficacy. Asterisks denote treatments that differ significantly from the reference level (Bonferroni-adjusted pairwise comparisons; ** *p* < 0.001). Inset: Slope plot summarizing the direction and magnitude of formulation effects within each EO family, comparing the parent EO to its corresponding RAMEB inclusion complex (R-EO) using the same integrated score. Upward slopes indicate improved efficacy after complexation, while downward slopes indicate reduced efficacy, illustrating that RAMEB reformulation produces family-specific shifts rather than a uniform advantage across all EOs.

To move beyond endpoint-specific comparisons and establish a unified hierarchy of treatment performance, a composite efficacy index integrating planktonic and biofilm readouts into a single standardized score were constructed. This integrated metric captures the net biological impact of each formulation across metabolic activity, viability, and biomass endpoints, thereby reducing endpoint-specific bias and highlighting consistent treatment-level effects. Linear mixed modeling was applied to the species-wise Z-transformed composite score to preserve within-species normalization while enabling cross-treatment comparison. This approach highlights the identification of globally superior regimens and, critically, permits the direct evaluation of whether RAMEB complexation confers a systematic efficacy advantage within each essential oil family. The integrated ranking and formulation-shift analysis are presented in Figure 6.

The mixed model demonstrated a pronounced overall regimen effect on the composite outcome (treatment: F(7, 40.752) = 24.552, *p* < 0.001), confirming that the experimental treatments differ substantially in their global efficacy across endpoints when assessed in an integrated framework. In contrast, the species main effect was negligible (F(3, 42.523) = 0.106, *p* = 0.956), which is consistent with the analytic design; because endpoints were Z-standardized within species, the composite score reflects within-species deviations rather than between-species baseline differences. Importantly, the model detected a significant species dependence of integrated efficacy, evidenced by a treatment × species interaction (F(21, 43.016) = 2.26, *p* = 0.012). This indicates that while some regimens are broadly effective, others show species-selective performance, and the “best regimen” cannot be assumed to be identical for all Candida species under the integrated scoring scheme. Estimated marginal means (EMMeans) for treatment, averaged across species, established a clear hierarchy of integrated efficacy (higher EMMeans = better overall inhibition). RT ranked as the strongest overall regimen (EMMeans = 0.475, 95% CI 0.346–0.603). Its confidence interval was entirely above zero, indicating consistently high performance relative to the grand mean across endpoints and species. Meanwhile RP ranked second (EMMeans = 0.106, 95% CI −0.024 to 0.235), representing a modest integrated benefit; its CI slightly crossed zero, suggesting greater variability across species/endpoints compared with RT. RB, however, followed with a negative mean (EMMeans = −0.175, 95% CI −0.304 to −0.045), indicating that, on average across species, RB did not match the integrated efficacy of RT and was not uniformly suppressive across all included endpoints. The remaining treatments formed a lower-performing group with negative EMMeans: B (−0.293), L (−0.371), T (−0.381), RL (−0.394), and P (−0.415) (lowest). These negative composite scores indicate comparatively weaker global suppression across the endpoint set, consistent with limited broad-spectrum efficacy under the integrated scoring framework. Multiplicity-controlled pairwise evidence supports RT as the top regimen among all treatments. RT was significantly superior to every other regimen, including the second-ranked RP (RT > RP: mean difference = 0.369, *p* = 0.005, Bonferroni-adjusted pairwise comparison). RT also exceeded RB and the lower-performing group by larger margins (e.g., RT > RB: difference = 0.649, *p* < 0.001), confirming that RT’s leading rank is statistically robust under conservative correction. For RP, pairwise comparisons showed that RP was clearly above the low-performing treatments overall, but the difference between RP and RB did not remain significant after Bonferroni adjustment (RP vs. RB: *p* = 0.100). This result supports that RP is generally a strong regimen, but its advantage over RB is not consistently large enough across the dataset to survive the most stringent correction, reflecting species-structured effects. In *C. albicans*, RT was decisively the top regimen, with a markedly elevated integrated score (RT = 0.862, 95% CI 0.671 −1.054), indicating strong and consistent inhibition across the endpoint set. RP was near neutral (RP = 0.028, CI crossing zero), and the weakest integrated performance in this species was observed for T (−0.466). This pattern supports RAMEB-encapsulated thyme oil as a broad and high-confidence regimen for *C. albicans*.

In *C. tropicalis*, RT again ranked highest (RT = 0.529, 95% CI 0.310–0.749). RP was close to neutral (−0.015), suggesting limited overall integrated benefit in this species. Notably, RB showed the lowest integrated mean (−0.405), indicating that RB’s performance is not uniformly favorable and may be comparatively weak against *C. tropicalis* when outcomes are integrated. This species therefore contributes to the interaction by separating RT from RB more strongly.

In the case of *C. krusei*, RT remained at the top regimen (RT = 0.453, 95% CI 0.233–0.672), and RP performed relatively well (0.143), consistent with RP having stronger efficacy in some species contexts than others. The weakest regimen in this species was RL (−0.517). Overall, *C. krusei* preserves the “RT-best” pattern but differs in the ordering and spacing of the remaining treatments, contributing to the interaction signal.

In contrast to the other three species, *C. dubliniensis* displayed a shifted integrated ranking, where RP emerged as the top regimen (RP = 0.266, 95% CI −0.004 to 0.536) and RB ranked second (0.141), while RT was comparatively weaker (RT = 0.054). Although RP’s CI narrowly crossed zero, the ordering indicates that *C. dubliniensis* responds preferentially to RP/RB relative to RT in the integrated framework. The poorest composite performance in this species was P (−0.529).

### 3.6. Response Classes Reveal Susceptible, Tolerant/Resistant, and Hormetic/Adaptive Phenotypes with Species Enrichment

**Figure 7:**
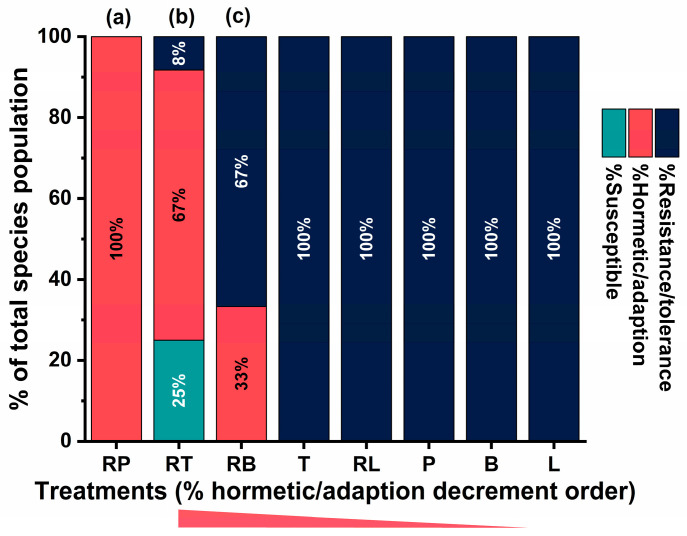
Response-class composition across treatments reveals differential enrichment of susceptible/adaptive versus tolerant/resistant Candida phenotypes. The stacked bar plots show, for each treatment, the percentage of the total species population assigned to the three inhibitory response classes derived from multivariate endpoint patterns: susceptible (strong inhibition), hormetic/adaptive (mixed or stimulation), and resistant/tolerance (weak inhibition) (color key on the right). The treatments are ordered by decreasing hormetic/adaptation fraction (*x*-axis label). The values within the bars indicate the within-treatment percentages for key classes. Across the experimental panel, RP, RT, and RB displayed the greatest heterogeneity in phenotype composition, including measurable hormetic/adaptive fractions (red) and a shift away from uniformly resistant/tolerant profiles, whereas the remaining treatments (T, RL, P, B, and L) were dominated by a single class (predominantly tolerant/resistant), indicating comparatively limited inhibitory engagement under those exposure conditions. The letters above the bars (a–c) denote treatment vs. species groupings: (a) Ca, Ct, Ck, Cd (hormetic/adaption): RP; (b) Ca, Ct, Ck, Cd (hormetic/adaption) and Ca, Ct, Ck (susceptible): RT; and (c) Ck, Cd (hormetic/adaption): RB.

To translate multidimensional efficacy patterns into biologically interpretable outcome states, treatment–species signatures were classified into discrete response phenotypes based on their integrated endpoint profiles. Using the unsupervised clustering of inhibition-coded functional readouts, each signature was assigned to one of three classes representing strong inhibition (susceptible), weak inhibition (tolerant/resistant), or mixed/hormetic-adaptive behavior. This phenotype-level abstraction enables the visualization of how each formulation distributes species across inhibitory states, rather than focusing solely on mean shifts. Importantly, this approach further revealed whether treatments drive uniform suppression across tested Candida species or generate heterogeneous responses suggestive of adaptation or tolerance emergence. The distribution of response classes across treatments is summarized in Figure 7.

To translate multi-endpoint responses into biologically interpretable phenotypes, response classes were derived using the unsupervised clustering of aggregated strain × treatment signatures. This approach integrated planktonic metabolism and viability with biofilm biomass, metabolic activity, and viability into a single multivariate response fingerprint, thereby enabling each strain–treatment pair to be assigned to one of three phenotypes: susceptible, hormetic/adaptive, or tolerant/resistant. Importantly, all endpoints contributing to these signatures had been Z-standardized within species, such that class membership reflected within-species deviations in response magnitude (i.e., the extent to which a given strain shifted relative to typical behavior of its own species), rather than baseline differences between species. Aggregated response fingerprints were constructed for four Candida species, including *C. albicans*, *C. tropicalis*, *C. krusei*, and *C. dubliniensis*. For each strain × treatment combination, the mean inhibition-coded responses were computed across endpoints representing planktonic inhibition (In-PMT, reflecting planktonic metabolism; and In-PVA, reflecting planktonic viability) and biofilm eradication (Er-BB, reflecting biofilm biomass; In-BMT, reflecting biofilm metabolic activity; and In-BVA, reflecting biofilm attached cellular viability). Because inhibition-coded metrics were analyzed (defined as the negative of the original species-wise Z values), larger positive values indicated stronger inhibition for the corresponding endpoint, whereas negative values indicated relative maintenance or enhancement of function under exposure in the standardized response space. The response-class analysis was performed across the experimental regimen set encoded as L, B, P, and T, representing base essential oil exposures in non-encapsulated form, and RL, RB, RP, and RT, representing the corresponding RAMEB-encapsulated formulations of the same treatment types. Consistent with earlier integrated ranking analyses in which RT and RP were placed at the top overall, this phenotype-based framework was used to provide an orthogonal interpretation focused on the qualitative response patterns induced across biological compartments.

Unsupervised learning was then applied to the aggregated inhibition-coded fingerprints using k-means clustering with k = 3, yielding three reproducible response classes. Biological meaning was inferred directly from the final cluster centers, which summarized the multivariate inhibition patterns associated with each phenotype. The first class was interpreted as a susceptible phenotype characterized by global, high-magnitude inhibition and therefore representing “true responders” under the standardized framework. In this class, strong planktonic suppression was observed at the cluster center (In-PMT = 1.57; In-PVA = 2.27), accompanied by a marked reduction in biofilm viability (In-BVA = 3.36). By contrast, the center values for biofilm biomass and biofilm metabolism were more modest and variable (Er-BB = −0.37; In-BMT = −0.58). This pattern supported an interpretation in which viability was compromised across compartments particularly within biofilms even when biomass and metabolic readouts did not show immediate or uniform inhibition, consistent with mechanisms that disrupt viability or core physiology without necessarily precipitating rapid biomass collapse.

The second class was interpreted as a hormetic/adaptive phenotype, capturing mixed or compartment-shifted inhibition patterns in which planktonic suppression was apparent while biofilm inhibition remained limited. At the class center, planktonic inhibition remained clearly positive (In-PMT = 1.40; In-PVA = 1.39), whereas biofilm endpoints clustered near neutral or negative values, most notably for biofilm viability (In-BVA = −0.07). Under this phenotype, strain–treatment pairs were inferred to exhibit strong inhibition in planktonic assays while failing to translate that inhibition into a comparably strong suppression of biofilm traits, a pattern consistent with adaptive compensation, biofilm-associated tolerance emergence, or hormetic-like responses in which stress exposure preferentially triggers protective pathways rather than producing uniform inhibition across compartments.

The third class was interpreted as a tolerant/resistant phenotype, representing weak inhibition across endpoints and consistent with minimal global efficacy under exposure. Negative inhibition-coded values were observed across the included endpoints at the class center (In-PMT = −0.57; In-PVA = −0.57; biofilm measures approximately −0.38 to −0.40), indicating that the strains assigned to this class maintained function across both planktonic and biofilm compartments relative to species-typical behavior in the standardized space. Under this interpretation, treatment regimens mapping to this phenotype were inferred to impose insufficient inhibitory pressure and/or to be countered by strain-level tolerance mechanisms that preserved viability and activity under the tested conditions. Across N = 75 aggregated strain × treatment fingerprints, the tolerant/resistant phenotype dominated the response landscape. Specifically, the susceptible phenotype (Class 1) was rare (*n* = 3; 4.0%), the hormetic/adaptive phenotype (Class 2) was observed at moderate frequency (*n* = 20; 26.7%), and the tolerant/resistant phenotype (Class 3) comprised the majority of signatures (*n* = 52; 69.3%). This distribution indicated that globally susceptible response profiles were uncommon, whereas partial or ineffective inhibition patterns predominated, thereby supporting the broader inference that many strain–treatment combinations failed to produce coordinated suppression across endpoints and compartments in this dataset.

Species representation in the clustered dataset was balanced, with totals of *n* = 25 for *C. albicans*, *n* = 17 for *C. tropicalis*, *n* = 17 for *C. krusei*, and *n* = 16 for *C. dubliniensis*. To improve statistical stability for enrichment testing, the classes were collapsed into a binary phenotype in which “Responder” combined the susceptible and hormetic/adaptive classes, and “tolerant/resistant” corresponded to the tolerant/resistant class alone. Under this collapsed definition, no evidence of association between responder status and species was detected (Pearson χ^2^(3) = 0.270, *p* = 0.966; N = 75), and expected counts were adequate (minimum expected count 4.91). These results indicated that responder versus tolerant/resistant phenotypes were not enriched within any species under the species-wise standardized response framework, suggesting that interspecies baseline differences were effectively controlled and that response heterogeneity was primarily driven by strain-level and regimen-level factors rather than species identity.

In contrast to the null species association, the response classes were strongly structured by regimen type, with a pronounced contrast between non-encapsulated exposures and RAMEB-encapsulated formulations. The susceptible phenotype was observed exclusively under RT exposure, indicating that RT uniquely generated globally strong inhibitory signatures across planktonic and biofilm endpoints in this dataset. This exclusivity provided a phenotype-level corroboration of earlier integrated ranking findings in which RT consistently emerged as the top-performing regimen, and it further suggested that RT was the only regimen capable of reliably driving strain–treatment signatures into the “true responder” state defined by coordinated inhibition. The hormetic/adaptive phenotype was enriched among the encapsulated regimens, being dominated by RP and RT and additionally represented by RB. Under this pattern, strong planktonic inhibition was frequently induced while biofilm inhibition remained incomplete, consistent with a regimen profile that imposed substantial stress yet permitted a compartmental shift toward biofilm-associated tolerance or adaptive compensation in a subset of strains. By contrast, the tolerant/resistant phenotype was heavily populated by the non-encapsulated regimens (L, B, P, and T) and by RL, indicating comparatively weak global inhibition across endpoints for those exposures. In practical terms, these regimens were most often associated with response fingerprints that remained outside the responder phenotypes, thereby providing a mechanistic and phenotypic explanation for their comparatively lower integrated efficacy. Taken together, the response-class framework complemented the integrated ranking results by specifying not only which regimen performed best, but also the phenotypes they most induced. RT was identified as the most reliable driver of the susceptible phenotype, reflecting global inhibition across compartments and aligning with its consistently high overall performance in prior analyses. RP, RT, and RB were frequently associated with hormetic/adaptive profiles, characterized by strong planktonic inhibition coupled with an incomplete suppression of biofilm traits, a pattern consistent with adaptive transitions toward biofilm-associated tolerant states. Meanwhile, the predominance of non-encapsulated regimens within the tolerant/resistant phenotype provided a clear phenotypic rationale for their weaker integrated performance, as these exposures most commonly failed to generate coordinated inhibitory pressure across the multi-endpoint response space.

### 3.7. Mechanistic Inference Stress and Antioxidant Gene Programs Predict Inhibitory Phenotypes

**Figure 8:**
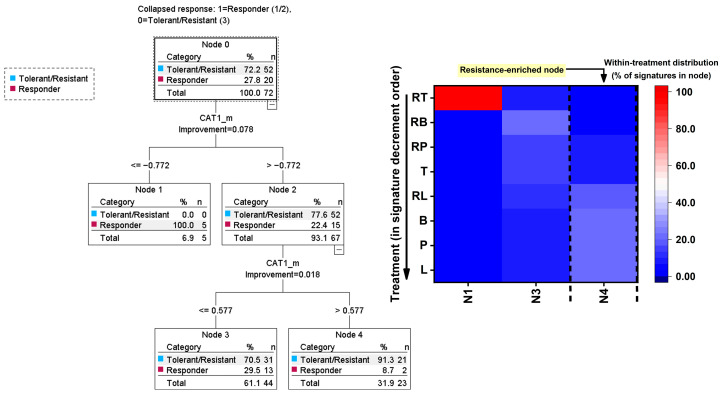
CART/CRT mechanistic decision tree links *CAT1* to responder status and reveals treatment enrichment across terminal nodes (experimental set, controls excluded). Left: Classification and regression tree (CRT; dependent variable Responder2, coded 1 = responder (susceptible + hormetic/adaptive) and 0 = tolerant/resistant) built from aggregated treatment × species mechanistic signatures. The tree identifies *CAT1*_m (mean *CAT1*) as the primary discriminating marker. The first split at *CAT1*_m ≤ −0.772 defines a high-responder node (N1; 100% responders; *n* = 5), whereas *CAT1*_m > −0.772 yields a mixed branch that is further separated by a second *CAT1*_m threshold (0.577) into N3 (responder-enriched; 29.5% responders; *n* = 44) and N4 (tolerant/resistant–enriched; 91.3% tolerant/resistant; *n* = 23). Node boxes report class proportions and sample sizes; improvement values reflect the split contribution to classification at each node. Right: The heatmap shows the within-treatment distribution of signatures across the terminal nodes (percent of each treatment’s signatures assigned to a node; rows sum to 100%). The treatments are ordered by decreasing global signature strength (as labeled). The dashed bracket highlights the resistance-enriched region (N4). RT is concentrated in the resistant-enriched node (N4), whereas RB and RP show broader dispersion across N1–N4, indicating more heterogeneous mechanistic–phenotypic behavior across species. Color scale: percentage (0–100%) of a treatment’s signatures falling into each terminal node (darker = lower; warmer = higher). The grey-highlighted zones indicate higher percentage distributions within the CART/CRT terminals.

To determine whether mechanistic stress signatures could predict functional inhibitory outcomes, the relationship between oxidative–nitrosative markers (ROS/RNS) and antioxidant response genes (*CAT1*, *GPX1*, and *SOD1*) and the derived inhibitory phenotype classes were modeled. Using aggregated treatment × species mechanistic signatures, a classification and regression tree (CRT) approach with Responder (responder vs. tolerant/resistant) as the dependent variable was applied. This analysis allowed the identification of hierarchical decision rules linking specific stress-response programs to phenotypic outcomes, thereby moving beyond descriptive clustering toward predictive mechanistic inference. The resulting tree structure and treatment-wise enrichment across terminal nodes are shown in Figure 8.

To connect the CRT-defined mechanistic programs to regimen identity, terminal node membership was cross-tabulated against treatment, and regimen enrichment was interpreted in the context of the node-wise class composition defined by the Responder outcome. In the full analysis (Supplementary Figure S2) that retained the antifungal and stress controls (AM, FL, and MN), treatment signatures were distributed non-randomly across the terminal nodes of the *GPX1*/*CAT1*-structured tree, indicating that specific regimens preferentially occupied distinct regions of the stress/antioxidant marker space rather than mapping uniformly across mechanistic programs. In this full model, the controls anchored opposite mechanistic extremes, with AM mapping exclusively to the high-confidence responder terminal program (Node 3; 9/9, 100%) and MN mapping exclusively to the tolerant/resistant-enriched terminal program (Node 5; 9/9, 100%), thereby validating that the CRT terminal states captured coherent and biologically separable mechanistic configurations. The second antifungal control, FL, was distributed across responder-enriched terminal programs, with strongest representation in Node 7 (7/12, 58.3%) and additional mapping to Node 2 (3/12, 25.0%) and Node 3 (2/12, 16.7%), and no mapping to the tolerant/resistant node, consistent with an overall responder-aligned mechanistic profile. Within the experimental regimens, responder-enriched programs were not confined to a single formulation, as the *GPX1*-high responder program (Node 2) was preferentially occupied by L (6/9, 66.7%), B (5/9, 55.6%), T (5/9, 55.6%), and RL (5/9, 55.6%), with a substantial contribution from P (4/9, 44.4%), whereas a second responder-enriched program (Node 7) was preferentially occupied by RB (6/9, 66.7%), with additional contributions from RL (3/9, 33.3%) and P (3/9, 33.3%). In contrast, RT exhibited pronounced heterogeneity in mechanistic mapping, with a substantial fraction aligning with the tolerant/resistant program (Node 5; 6/12, 50.0%) and the remainder distributed across responder-enriched and mixed programs, while RP preferentially occupied the mixed/ambiguous terminal program (Node 8; 4/9, 44.4%), consistent with context-dependent mechanistic engagement under the CRT rule set.

Because control-driven anchoring can inflate apparent separability and obscure the finer regimen structure among experimental exposures, a second CRT was evaluated after exclusion of A, F, and MN (Figure 8). In this experimental-only dataset (N = 72), node membership differed significantly by treatment identity (Pearson χ^2^(14) = 51.162, *p* < 0.001), although the contingency table remained sparse and enrichment was therefore interpreted descriptively. In this restricted analysis, terminal node membership segregated strongly by regimen, and a single terminal node was composed exclusively of RT signatures (Node 1; 5/5, 100%), indicating that RT occupied a distinct *CAT1*-defined mechanistic region when the controls were removed. This RT-only node represented 55.6% of all RT observations in the experimental-only subset (5/9), while the remaining RT signatures mapped to the dominant mixed node (Node 3; 4/9, 44.4%) and did not appear in Node 4. The largest terminal node (Node 3; *n* = 44) captured the majority of experimental signatures and contained all RB observations (9/9, 100%), along with high within-treatment representation for B (7/9, 77.8%), T (7/9, 77.8%), and RP (7/9, 77.8%), and moderate representation for P (5/9, 55.6%), RL (5/9, 55.6%), and L (4/9, 44.4%). The remaining terminal node (Node 4; *n* = 23) concentrated L signatures (5/9, 55.6%) and included substantial contributions from P (4/9, 44.4%) and RL (4/9, 44.4%), with smaller contributions from B (2/9, 22.2%), T (2/9, 22.2%), and RP (2/9, 22.2%), while remaining entirely unoccupied by RB (0/9, 0%) and RT (0/9, 0%). Thus, after the exclusion of the controls, regimen stratification persisted but was expressed primarily as differential occupation of *CAT1*-defined terminal programs, with RB consistently mapping to the dominant mixed terminal node, RT partially isolating into a treatment-specific terminal node, and L/P/RL showing relatively greater representation in the alternative terminal node.

### 3.8. Multivariate Mode-of-Action Landscape Defines Treatment Signatures and Mechanistic Axes

**Figure 9:**
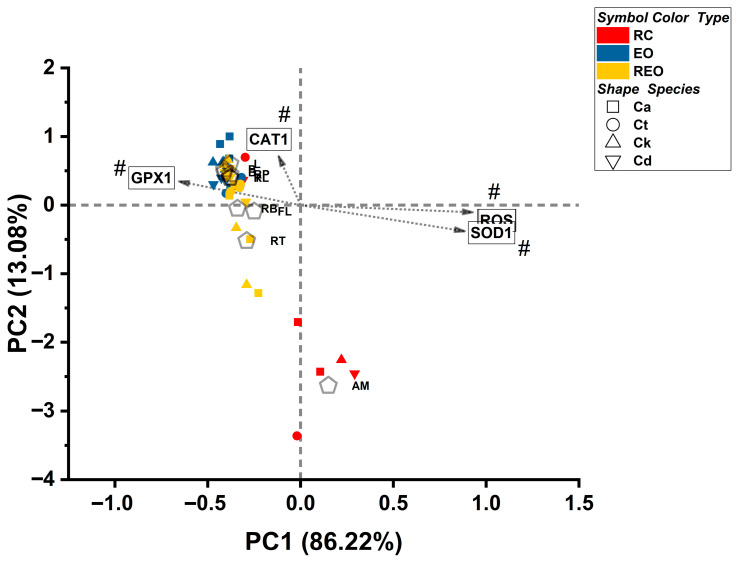
Multivariate mode-of-action landscape separates stress-intensity and antioxidant-program axes across treatments and species. A principal component analysis (PCA) was performed on the aggregated treatment × species mechanistic signatures (RNS, ROS, *CAT1*, *GPX1*, and *SOD1*; Z-transformed within species). The biplot shows PC1 (86.22%) versus PC2 (13.08%) for each treatment–species signature. The points are colored by treatment type (RC = antifungal reference controls, EO = essential oils, and REO = RAMEB-EO complexes) and shaped by Candida species (Ca, Ct, Ck, and Cd). The dashed crosshairs indicate the origin (0, 0). The gray arrows depict rotated loading vectors: PC1 captures a dominant stress/redox intensity axis (positive direction aligned with ROS and *SOD1*, opposed to *GPX1*), whereas PC2 reflects a *CAT1*-centered antioxidant-response axis (positive direction aligned with *CAT1*). The centroid labels (treatment codes) indicate the mean position of each treatment across species. The hash marks (#) denote variables with the strongest contributions to the displayed axes (highest absolute loadings). Note: MN is excluded from this panel to prevent the stress-control anchor from compressing the experimental space; full PCA including MN is provided in the Supplementary Materials (Figure S3).

To integrate oxidative–nitrosative stress markers and antioxidant gene responses into a unified mechanistic framework, a principal component analysis (PCA) on the aggregated treatment × species signatures was performed. Because all variables were standardized within species, this multivariate approach captures relative mechanistic positioning rather than potency differences, enabling the comparison of mode-of-action geometry across treatments. The PCA reduced the five-dimensional stress-response space (RNS, ROS, *CAT1*, *GPX1*, and *SOD1*) into orthogonal axes that summarize dominant patterns of covariation. The resulting biplot (Figure 9) visualizes how treatments and species distribute along a primary stress-intensity axis and a secondary antioxidant-program axis, thereby defining the global mechanistic landscape of the EO and RAMEB-EO formulations.

Component interpretation was guided by variable vectors projected onto PC space. PC1 was aligned with a strong oxidative-stress direction, as ROS projected prominently in the positive PC1 direction and co-varied with the *SOD1* vector, while *GPX1* projected strongly in the opposing (negative PC1) direction. PC2 was dominated by the catalase axis, with *CAT1* projecting primarily along the positive PC2 direction. Thus, the MoA plane was interpreted as contrasting ROS/*SOD1*-associated stress programs against *GPX1*-associated antioxidant configurations along PC1, with *CAT1*-associated variations represented orthogonally along PC2. Because treatment centroids and strain-level points were overlaid in the same space, these axes were interpreted as integrated MoA dimensions reflecting coordinated shifts in mechanistic marker configuration across strain–treatment signatures rather than isolated single-marker effects. Two visualizations were required to preserve interpretability across the full mechanistic range of the dataset. In the full MoA map (Supplementary Figure S3), the inclusion of the oxidative stress control MN generated an extreme displacement along positive PC1 in the direction of ROS (and toward the *SOD1*-aligned quadrant), thereby anchoring the oxidative-stress end of the landscape but simultaneously compressing the remaining treatments near the origin when a single global scale was applied. For clarity of the experimental regimen structure, a zoomed view (the first image) was therefore examined in which MN was excluded from the plotted space, allowing separation among the remaining treatments to be resolved without altering the underlying PCA solution or its mechanistic interpretation.

Treatment positioning in PC space was non-random and mechanistically interpretable in both views. In the full map, MN was separated far to the right on PC1, consistent with a distinct ROS-dominated stress signature that was not approached by any experimental regimen, thereby providing an internal validation that the PCA captured a coherent oxidative-stress program. The antifungal control AM separated strongly along negative PC2, placing it opposite the *CAT1* vector and indicating a mechanistically distinct program characterized by displacement away from the *CAT1*-aligned direction relative to the experimental cluster. The remaining regimens formed a compact treatment-defined constellation near the negative PC1 region, indicating broad alignment toward the *GPX1*-associated direction and away from the MN-defined ROS extreme. Within this constellation, the base (non-encapsulated) experimental regimens (L, B, P, and T) clustered tightly with the corresponding encapsulated formulations (RL, RB, and RP), indicating that most experimental exposures shared a broadly similar multivariate stress/antioxidant configuration in this MoA space, with relatively subtle between-regimen differences compared with the control anchors. Notably, RT showed the most consistent displacement among the experimental regimens by shifting downward relative to the main cluster (negative PC2), placing it farther from the *CAT1*-aligned direction than most other experimental signatures and indicating a distinctive catalase-opposed mechanistic positioning within regimen space. FL mapped within the central experimental constellation and overlapped most strongly with the region occupied by RB and the densely clustered L/B/P/T/RL/RP signatures, consistent with a responder-aligned but not extreme mechanistic configuration relative to the two control anchors. Formal multivariate testing of the PCA scores (listwise N = 96) supported the visual inference that the MoA landscape was treatment-defined. Treatment identity significantly structured the multivariate PC space (Pillai’s Trace = 1.667; F(18,172) = 47.82; *p* < 0.001), with significant treatment effects detected on both axes (PC1: F(9,86) = 46.27, *p* < 0.001, R^2^ = 0.829; PC2: F(9,86) = 49.46, *p* < 0.001, R^2^ = 0.838). In contrast, species did not structure the global PC1-PC2 MoA space (Pillai’s Trace = 0.0147; F(6184) = 0.226; *p* = 0.968), indicating that the dominant multivariate organization reflected regimen-associated signature shifts rather than baseline species separation.

Overall, the MoA landscape was defined primarily by treatment identity, with MN and AM providing mechanistically interpretable anchors at opposite extremes of the space. MN uniquely occupied the ROS/*SOD1*-aligned stress extreme on PC1 (3.08), whereas AM separated most strongly along PC2 (−2.63) in the direction opposing *CAT1*. Most experimental regimens (L: −0.38; B: −0.41; P: −0.41; T: −0.38; RL: −0.38; RB: −0.34; and RP: −0.38) occupied a compact region toward negative PC1 consistent with relative alignment to the *GPX1*-associated direction and away from ROS-driven stress, while RT exhibited the clearest within-experimental deviation by shifting toward negative PC2 (−0.52), indicating a distinctive catalase-opposed positioning within the treatment-defined MoA space. The complete data on the eigenvalues, rotated component matrix of the mechanistic markers and principal component centroid values can be found in Supplementary Tables S9–S11.

## 4. Discussions

The rising incidence of life-threatening fungal infections driven in part by advanced medical care that expands the population of immune-compromised patients has intensified the demand for effective clinical interventions. This challenge is compounded by dose-limiting antifungal toxicity and the rapid emergence of antifungal resistance, underscoring an urgent need for innovative therapeutic agents and strategies. In this study, we evaluated the effects of sub-inhibitory concentrations of four Lamiaceae essential oils, lavender (*Lavandula angustifolia* Mill.), lemon balm (*Melissa officinalis* L.), peppermint (*Mentha piperita* L.), and thyme (*Thymus satureioides*), and their RAMEB counterparts against four Candida species (*C. albicans*, *C. tropicalis*, *C. krusei*, and *C. dubliniensis*). Because cyclodextrin-based inclusion systems can modify the aqueous availability and free (bioactive) fraction of lipophilic small molecules, RAMEB encapsulation is expected to alter not only the total deliverable dose but also the concentration–time profile experienced by cells within planktonic and biofilm states. Comparative multivariate analyses (MANOVAs) combined with unsupervised stratification (k-means clustering) revealed a clear hierarchy of efficacy that appears to be driven by the chemical identity of the dominant constituents and their interaction with fungal stress-response capacity, particularly under sublethal exposure conditions. The inclusion of AM and FL enabled the direct benchmarking of EO-RAMEB efficacy relative to clinically used antifungal agents, while also providing mechanistic reference anchors within the oxidative–nitrosative response framework. The mechanistic interpretations below are therefore framed as literature-consistent hypotheses that explain the phenotypic and multivariate patterns observed here, while recognizing that confirmatory molecular assays (e.g., ROS quantification, enzymatic readouts, and transcriptomics) will be required to establish causality. However, it should be noted that the mechanistic variables were analyzed using species-wise standardization, which emphasizes relative response patterns rather than absolute interspecies differences. Accordingly, the observed conservation reflects similarity in treatment-induced response architecture rather than equivalence in baseline or magnitude of oxidative–nitrosative responses. Importantly, the absence of species-level differences in standardized CFU responses should not be interpreted as a lack of species-specific susceptibility. Rather, because CFU values were normalized within species (Z-transformation), this analysis reflects relative deviations from species-specific baselines and is therefore not designed to capture the absolute interspecies differences observed in MIC_90_ and EC_10_ endpoints. Thus, susceptibility in this study is defined at the level of concentration–response architecture rather than absolute survival output under normalized conditions. Additionally, the phase plasticity metric (Δs and EC_10_/MIC_90_) introduced in this study is intended as a descriptive parameter of concentration–response architecture rather than a validated predictive indicator. It reflects the breadth of the sub-inhibitory response window and is conceptually related to tolerance-associated phenomena such as trailing growth, fractional growth, and supra-MIC growth. However, unlike these measures, phase plasticity integrates both early-response sensitivity and inhibitory thresholds. While useful for comparative analysis across species and treatments, it requires formal validation against established tolerance metrics and clinical relevance and should therefore be interpreted as a quantitative descriptor of response topology rather than a surrogate marker of clinical tolerance.

The antifungal activity observed in this study can be interpreted in the context of established mechanisms associated with essential oil constituents. Terpenoid compounds such as thymol, menthol, citral, and linalool are known to disrupt fungal cell membranes by altering lipid packing, increasing permeability, and interfering with ergosterol-dependent membrane integrity. These effects can lead to ion leakage, mitochondrial dysfunction, and impaired cellular homeostasis. In parallel, essential oils have been reported to induce oxidative and nitrosative stress through increased intracellular ROS/RNS generation, contributing to cellular damage and growth inhibition.

In the present study, the oxidative–nitrosative profiling revealed predominantly moderate stress signatures accompanied by *CAT1* and *GPX1* activation, suggesting that EO exposure induces a controlled redox imbalance rather than acute oxidative collapse. This is consistent with a model in which fungal cells initially engage antioxidant defenses but fail to fully compensate under effective treatment conditions. The enhanced activity observed for specific RAMEB-EO formulations, particularly RT and RB, likely reflects the improved solubilization and bioavailability of lipophilic compounds, facilitating membrane interaction and intracellular accumulation. Collectively, these findings support a multifactorial mechanism involving membrane perturbation, redox imbalance, and impaired stress adaptation rather than a single dominant mode of action.

At the top of this hierarchy, thyme (RT) emerged as the most broadly active formulation, consistent with its phenolic monoterpene-rich profile dominated by thymol and carvacrol [12,31]. These components are widely associated with membrane-active antifungal effects and may promote leakage of intracellular constituents (e.g., ATP, K^+^) alongside disruption of mitochondrial energy homeostasis. In addition, thymol has been reported to induce oxidative stress through reactive oxygen species (ROS) generation and lipid peroxidation, which can burden NADPH-dependent antioxidant systems. In this context, oxidative stress may deplete the NADPH required to regenerate reduced glutathione (GSH), thereby compromising NADPH-dependent antioxidant enzymes such as glutathione reductase and diminishing the capacity to neutralize ROS, ultimately amplifying oxidative damage [32,33,34]. Notably, unlike the other tested oils, RT exhibited a “catalase-opposed” -like signature at the phenotypic level, consistent with the possibility that RT either bypasses or functionally suppresses genus-wide *CAT1*-associated defense capacity. In turn, this may reduce the likelihood that Candida transitions into an adaptive/tolerant state and instead favors a coordinated loss of viability across planktonic and biofilm-associated cells [35]. This interpretation is supported by RT’s exclusive placement within a high-efficacy terminal node in the classification and regression tree (CRT) model and by its distinctive displacement in the PCA space, a pattern that mirrors the multivariate signature of potent clinical antifungals such as amphotericin B [33,36,37]. The composite efficacy score was derived by integrating multiple standardized endpoints under an equal-weighting framework, which, while enabling cross-endpoint comparability, may not fully capture the differential biological contribution or hierarchical importance of individual parameters.

In contrast, peppermint (RP) and lemon balm (RB) behaved as comparatively “selective” inhibitors and, importantly, displayed features compatible with an increased risk of hormesis-like or adaptive responses under sub-inhibitory exposure. Although these oils showed marked activity against planktonic Candida populations, they did not achieve complete sterilization within biofilm architecture, indicating a reproducible disparity between planktonic killing and biofilm eradication (i.e., a planktonic-biofilm decoupling of efficacy). A mechanistically plausible contributor is the sequestration of non-phenolic monoterpenes, such as menthone (peppermint) and citral (lemon balm) within the extracellular polymeric substance (EPS) matrix. The EPS, a heterogeneous scaffold containing β-1,3-glucans, mannans, and extracellular DNA (eDNA), can function as a physicochemical sink; its hydrophobic domains facilitate non-specific interactions with less polar ligands, attenuating mass transfer kinetics and effectively retaining antimicrobial molecules in the upper strata of the matrix. Consequently, the local concentrations reaching deeper biofilm layers may remain below the threshold required for a “total kill,” allowing basal-layer survivors to persist and mount adaptive responses [38,39,40,41].

The observed species-dependent variability in susceptibility can be interpreted in the context of known biological differences among Candida species. Non-albicans Candida species, including *C. tropicalis*, *C. krusei*, and *C. dubliniensis*, are reported to exhibit alterations in cell wall architecture, including increased β-glucan and chitin content, which can reduce permeability to antifungal agents and contribute to intrinsic resistance. In addition, differences in membrane lipid composition and ergosterol organization may influence susceptibility to membrane-active compounds such as essential oil terpenoids [39,40,41].

Efflux-mediated resistance mechanisms may also contribute to the observed patterns, as several non-albicans species exhibit higher activity of ATP-binding cassette (ABC) and major facilitator superfamily (MFS) transporters, which can limit the intracellular accumulation of xenobiotic compounds. Furthermore, biofilm-associated resistance varies across species, with differences in extracellular matrix composition and density influencing diffusion and local drug availability [32,33,34].

These biological factors are consistent with the present findings, where non-albicans species required higher concentrations for both inhibitory (MIC_90_) and sublethal (EC_10_) effects and exhibited broader MIC-EC separation (phase plasticity), indicating an expanded adaptive response window. In contrast, *C. albicans* displayed comparatively higher susceptibility and a narrower transition between sublethal and inhibitory regimes, suggesting reduced tolerance capacity under the tested conditions.

At the molecular level, menthol in RP and geranial in RB may exert sublethal pressure that preferentially engages antioxidant reconfiguration, consistent with a *GPX1*-skewed defensive state. Prior studies report that sub-lethal menthol exposure (e.g., 0.5–1 mM) can induce oxidative stress in Candida by compromising membrane integrity and altering respiratory/oxidative phosphorylation dynamics, thereby activating antioxidant defense mechanisms as the fungus attempts to restore homeostasis [42,43,44,45,46]. Under these conditions, fungal cells may be more likely to activate protective pathways and enter a state of biofilm-associated tolerance. However, a notable species-specific “crossover effect” was observed for *C. dubliniensis*, which—despite being generally less responsive to thyme—proved uniquely vulnerable to RP and RB. Two non-exclusive explanations may account for this pattern. First, the species- or strain-dependent differences in the cell wall architecture and/or EPS composition in *C. dubliniensis* may alter the partitioning, retention, or local bioavailability of cyclic monoterpenes and phenylpropanoids relative to *C. albicans*, thereby shifting effective exposure to the cell surface within the biofilm matrix [52]. Second, differential membrane sterol homeostasis and growth-state biology may contribute mutations in ERG3 (sterol C5,6-desaturase) and ERG11 (lanosterol 14α-demethylase), which are well-recognized mechanisms that perturb ergosterol biosynthesis and can contribute to reduced susceptibility or resistance to azole agents or functionally azole-like compounds [4,5,28]. In parallel, although *C. dubliniensis* can form hyphae, its filamentation kinetics and morphogenetic dynamics are frequently distinct and often slower than those of *C. albicans*. It is therefore plausible that the rapid filamentation program of *C. albicans* may be particularly vulnerable to membrane-active compounds, given that monoterpenes can disrupt membrane organization during polarized growth associated with germ-tube emergence. At sub-inhibitory concentrations, cells are not killed but may experience substantial physiological stress, and *C. albicans* relies on conserved stress-response signaling pathways (including the calcineurin/Crz1 pathway) to tolerate and adapt to these conditions [53].

The limited activity of lavender (RL) across taxa further underscores the requirement to exceed a “molecular threshold” to meaningfully perturb Candida homeostasis and overcome basal defense programs. Both the pure oil (L) and its RAMEB-encapsulated form (RL) consistently mapped to a tolerant/resistant-like phenotype. In the PCA landscape, lavender treatments did not displace the cellular response away from baseline, suggesting that the effective concentrations of linalool and linalyl acetate were insufficient to disrupt homeostatic function or did not produce significant inhibition under our sub-MIC conditions. Lavender formulations dominated by linalool/linalyl acetate may remain functionally sub-inhibitory under our conditions, as linalyl acetate has been reported to show comparatively low fungicidal activity against *C. albicans*, cyclodextrin complexation can buffer the free bioactive fraction, and Candida biofilm matrix (e.g., β-1,3-glucan/eDNA) can further sequester antimicrobials and attenuate effective exposure [54,55,56,57].

Accordingly, in the absence of strongly membrane-disruptive phenolic monoterpenes (e.g., thymol/carvacrol-like constituents) or more pronounced metabolic stressors (e.g., menthol-associated effects), lavender (L and RL) remained effectively sub-inhibitory under our assay conditions, with responses clustering near baseline and indicating that Candida can buffer or neutralize these exposures without a major shift in the measured stress/viability phenotypes.

Across treatments, our data also reveals a critical decoupling between biofilm physical structure and biological viability. Physical biofilm biomass remained robust and relatively insensitive even when cellular viability was markedly suppressed, indicating that while RAMEB-encapsulated oils, particularly thyme, can penetrate the EPS sufficiently to exert biocidal effects on encased yeast, they do not dismantle the underlying β-glucan and chitin-enriched scaffolding that maintains biofilm integrity [27,52,58]. Consequently, the transition toward an effective “responder” phenotype appears to depend strongly on the RAMEB carrier’s capacity to maximize and to sustain the bioavailable (free) fraction of these lipophilic compounds within the biofilm environment. This formulation dependence is likely exacerbated by the intrinsic volatility and hydrophobicity of essential oil constituents and by standard microplate assay geometry; under non-encapsulated conditions, rapid headspace loss, adsorption to plastic surfaces, and phase partitioning can reduce effective aqueous concentration over time and promote sub-inhibitory exposure gradients within the matrix [59,60,61]. Accordingly, even high-potency oils such as thyme may fail to maintain inhibitory exposure throughout the biofilm, increasing the likelihood of survivor persistence and tolerance-like phenotypes. Together, these considerations emphasize that successful Candida eradication requires both an appropriate chemical mode of action and an optimized delivery system [62,63,64,65].

Finally, the broad-spectrum profile of the RT formulation (relative to RP, RB, and RL) is consistent with the cyclodextrin-mediated modulation of free-fraction exposure rather than a simple increase in total dose. The inclusion complexes of phenolic monoterpenes (e.g., thymol and carvacrol) with β-cyclodextrin derivatives exhibit measurable formation (stability) constants that shift the free (bioavailable) fraction; stronger complexation can dampen peak free concentrations while enabling a reservoir effect that sustains delivery as free molecules are depleted at the cell interface [66]. In applied systems, the β-cyclodextrin encapsulation of thyme essential oil has been shown to support gradual volatile release over time, consistent with a controlled-delivery mechanism [67]. Moreover, cyclodextrins (including randomly methylated derivatives) can exert carrier-dependent biological effects on Candida biofilm formation and morphology, which should be considered when interpreting species-level responses to RAMEB formulations [68]. A key pharmacodynamic implication is that controlled release is beneficial only if the resulting concentration–time profile remains at or above effective inhibitory levels; if complexation and release kinetics generate prolonged sub-inhibitory “tails,” Candida may experience persistent non-lethal stress that promotes phenotypic adaptation (including altered metabolic/colony phenotypes under sub-MIC essential oil exposure) [69] and engages conserved stress circuitry (including Ca^2+^/calcineurin-linked responses to membrane-active monoterpenes such as carvacrol) [53]. Because antifungal tolerance (supra-MIC growth of susceptible populations) is distinct from resistance and can contribute to persistent infection [28], and because experimental evolution demonstrates that different drug concentrations can select distinct adaptive trajectories (including tolerance- versus resistance-associated outcomes) [9,28,70], an unfavorable slow-release regime could plausibly increase the probability of hormesis-like or tolerance phenotypes and under repeated exposures selection toward reduced susceptibility [8,9,70]. The essential oils used in this study were evaluated using a single standardized batch, ensuring internal consistency across all experimental conditions. However, GC-MS-based batch-specific compositional profiling was not performed, which limits the attribution of antifungal activity to individual chemical constituents and precludes the assessment of inter-batch variability. Importantly, the selected essential oils are well characterized in the literature with established dominant bioactive constituents (e.g., thymol/carvacrol in thyme, menthol in peppermint, citral/estragole in lemon balm, and linalool in lavender), which were considered in the interpretation of formulation-dependent effects [71,72,73,74,75,76]. Accordingly, the present findings should be interpreted at the level of integrated formulation response rather than compound-specific mechanisms. Future studies integrating compositional analysis and quantitative loading characterization will be required to establish direct structure–activity relationships.

Despite the strengths of the multi-parameter and species-resolved analytical framework, several limitations should be considered when interpreting the present findings. The biofilms were evaluated at a 24 h maturation stage using functional readouts (metabolic activity, viability, and biomass) without detailed structural characterization. While this approach enables standardized comparative analysis across treatments and species, it does not fully capture the architectural complexity and heterogeneity of mature biofilms. In addition, the use of EC_10_ concentrations reflects a sublethal exposure paradigm and may therefore underestimate the maximal antibiofilm efficacy achievable at higher exposure levels, particularly in the context of matrix disruption or deep biofilm penetration. The composite efficacy score, constructed using an equal-weight integration of standardized endpoints, further enables cross-endpoint comparison but may not fully account for the differential biological relevance of individual parameters. Furthermore, the observed decoupling between reductions in cellular viability and the persistence of biofilm biomass suggests that the treatments effectively impaired biofilm-associated cells without fully disrupting the structural matrix. One possible explanation is the limited penetration or interaction of lipophilic monoterpenes within the extracellular polymeric substance (EPS) network, which may act as a diffusion barrier or sequestration environment [39]. However, this mechanism was not directly assessed in the present study and therefore should be considered a plausible interpretation rather than a demonstrated effect.

Despite the comprehensive in vitro characterization presented in this study, several limitations should be acknowledged. First, the findings are based on controlled in vitro systems and do not account for host-related factors that may influence antifungal efficacy in vivo, including immune interactions, tissue distribution, and metabolic stability.

From a translational perspective, the absence of cytotoxicity and selectivity profiling represents an important limitation, as it precludes direct assessment of therapeutic windows for the evaluated formulations. Furthermore, while RAMEB-based delivery enhanced the functional performance of selected essential oils, the detailed physicochemical characterization of inclusion complex dynamics and compound-specific release behavior remains to be further explored.

Future studies should therefore integrate concentration–response expansion, advanced biofilm models, cytotoxicity profiling in mammalian systems, and mechanistic validation at the molecular level to support the development of optimized, species-stratified antifungal strategies.

Overall, the present findings are consistent with previous reports demonstrating the antifungal activity of essential oils against Candida species, while also highlighting the substantial variability in species susceptibility and treatment efficacy observed across studies. Such differences are likely driven by variations in essential oil composition, formulation strategies, and experimental conditions. In this context, the enhanced performance of selected RAMEB-EO formulations supports earlier evidence that cyclodextrin-based delivery can improve the functional availability of hydrophobic constituents. Importantly, the multi-parameter framework applied here, integrating inhibitory thresholds with sublethal response behavior and mechanistic profiling, provides a more comprehensive characterization of antifungal activity compared to conventional single-endpoint approaches. This may account for discrepancies with earlier studies and underscores the value of systems-level analysis in resolving species-dependent response patterns and refining antifungal evaluation strategies.

## 5. Conclusions

This study demonstrates that converting essential oils into RAMEB inclusion complexes does not yield a uniform antifungal upgrade across Candida species; rather, encapsulation produces oil-family-specific and species-dependent shifts in susceptibility, functional inhibition, and response phenotypes. Although oxidative–nitrosative stress responses appeared broadly conserved, the ability of each regimen to attain and maintain effective inhibitory exposure thereby suppressing planktonic growth and early biofilm viability varied substantially among formulations. Within this landscape, RT was the most consistently inhibitory regimen across endpoints, supporting its prioritization as the lead broad-spectrum candidate. The RAMEB complexation did not uniformly enhance antifungal efficacy across all essential oil formulations, but instead exerted treatment-specific and species-dependent effects. Notably, RAMEB-thyme (RT) consistently demonstrated superior performance across multiple endpoints, whereas other formulations (RL, RB, and RP) exhibited variable outcomes, highlighting that cyclodextrin-based delivery acts as a selective optimization strategy rather than a universally enhancing system. In contrast, RP showed the strongest overall generalist performance but exhibited species-contingent behavior that warrants strain- and species-aware interpretation, particularly in contexts where sub-inhibitory exposure could favor persistence or tolerance-like adaptation. Collectively, these findings argue for a development pathway in which RAMEB encapsulation is optimized and benchmarked empirically for each essential oil family and target species, advanced preferentially when it produces durable multi-endpoint inhibition, and applied cautiously when phenotypic signatures indicate weak pressure, compartmentalized biofilm effects, or adaptive/tolerance-like trajectories. Overall, the present findings are consistent with previous reports demonstrating the antifungal activity of essential oils against Candida species, while also highlighting the substantial variability in species susceptibility and treatment efficacy observed across studies. Such differences are likely driven by variations in essential oil composition, formulation strategies, and experimental conditions. In this context, the enhanced performance of selected RAMEB-EO formulations supports earlier evidence that cyclodextrin-based delivery can improve the functional availability of hydrophobic constituents. Importantly, the multi-parameter framework applied here, integrating inhibitory thresholds with sublethal response behavior and mechanistic profiling, provides a more comprehensive characterization of antifungal activity compared to conventional single-endpoint approaches. This may account for discrepancies with earlier studies and underscores the value of systems-level analysis in resolving species-dependent response patterns and refining antifungal evaluation strategies.

## Figures and Tables

**Table 1:** Final cluster centers of oxidative–nitrosative responses calculated using k-clustering method.

Parameter	Cluster		
	1 *	2 **	3 ***
RNS	2.904	−0.54	0.60
ROS	2.839	−0.43	0.92
*CAT1*	−0.372	0.66	−2.42
*GPX1*	−2.6	0.63	−1.11
*SOD1*	2.616	0.839	1.15

* Cluster 1: high oxidative–nitrosative stress with *SOD1* dominance and *GPX1* suppression (*n* = 4); ** cluster 2: moderate stress with suppressed *CAT1*/*GPX1* response (*n* = 7); *** cluster 3: low measured stress with *CAT1*/*GPX1* upshift (adaptive/low-stress class; *n* = 33).

## Data Availability

The original contributions presented in this study are included in the article/Supplementary Material. Further inquiries can be directed to the corresponding author.

## Supplementary Materials

The following supporting information can be downloaded at https://www.mdpi.com/article/10.3390/pharmaceutics18040508/s1, Figure S1: Antioxidant-gene response landscape across treatments (*CAT1*, *GPX1*, *SOD1*); Figure S2: CRT identifies *GPX1*-*CAT1* thresholds that separate responder vs. tolerant/resistant phenotypes and shows treatment enrichment across terminal nodes (controls included); Figure S3: Global multivariate mode-of-action landscape separates stress-intensity and antioxidant-program axes across treatments and species; Figure S4: Representative crystal violet stained biofilm biomass images of *Candida albicans* (Ca1372) under treatment conditions; Figure S5: Representative crystal violet stained biofilm biomass images of *Candida albicans* (Ca1423) under treatment conditions; Figure S6: Representative crystal violet stained biofilm biomass images of *Candida albicans* (Ca1424) under treatment conditions; Figure S7: Representative crystal violet stained biofilm biomass images of *Candida tropicalis* (Ct1368) under treatment conditions; Figure S8: Representative crystal violet stained biofilm biomass images of *Candida tropicalis* (Ct1432) under treatment conditions; Figure S9: Representative crystal violet stained biofilm biomass images of *Candida krusei* (Ck779) under treatment conditions; Figure S10: Representative crystal violet stained biofilm biomass images of *Candida krusei* (Ck1447) under treatment conditions; Figure S11: Representative crystal violet stained biofilm biomass images of *Candida dubliniensis* (Cd1470) under treatment conditions; Figure S12: Representative crystal violet stained biofilm biomass images of *Candida dubliniensis* (Cd1471) under treatment conditions; Figure S13: Representative crystal violet stained biofilm biomass images of background control (BC), untreated Candida species biofilm controls (UBCs); Figure S14: Lemon balm RIC chromatogram: In the upper panel, citral [M+H]+; in the lower panel, citral [M+H−H_2_O]+; Figure S15: Mass spectrum of the lemon balm oil sample. Upper panel: peak spectrum at t_R_ 9.86; lower panel: peak spectrum at t_R_ 10.09; Figure S16: EIC chromatogram of the citral standard. In the upper panel, citral [M+H]+; in the lower panel, citral [M+H−H_2_O]+; Figure S17: Mass spectrum of the citral standard. Upper panel: peak spectrum at t_R_ 9.87; lower panel: peak spectrum at t_R_ 10.10; Table S1: Consumables used throughout the studies; Table S2: Oligonucleotide primers and amplicon sizes for quantitative real-time PCR (qPCR) analysis of Candida species genes; Table S3: Minimum inhibitory concentration (MIC_90_) raw data; Table S4: Minimum effective concentration (EC_10_) raw data; Table S5: Reactive nitrogen species (RNS) and reactive oxygen species (ROS) generation in different Candida strains; Table S6: Reduced percentage in planktonic metabolism (PMT) and viability (PVA) compared to untreated control (UC); Table S7: Reduced percentage in total biofilm biomass (BB), biofilm attached cellular metabolic activity (BMT) and viability (BVA) compared to untreated biofilm control (UBC); Table S8: Pairwise distance matrix from hierarchical clustering of treatment-induced mechanistic signatures (ROS/RNS and antioxidant gene responses); Table S9: Principal component analysis of mechanistic markers: eigenvalues and variance explained (unrotated and Varimax-rotated solutions); Table S10: Rotated component matrix (Varimax) from PCA: loading vectors of ROS/RNS and antioxidant genes; Table S11: Treatment centroids on the PCA mechanistic landscape (PC1-PC2) derived from within-species Z-standardized ROS/RNS and antioxidant gene signatures (*CAT1*, *GPX1*, *SOD1*); Table S12: HPLC-HRMS-based annotation of major and minor constituents detected in lemon balm essential oil; Table S13: HPLC-HRMS gradient program.

## Abbreviations

The following abbreviations were used in this manuscript:

%GC	guanine-cytosine percentage content
AM	amphotericin B
AUC	area under curve
B	lemon balm essential oil
BB	biofilm biomass
BC	background control
BMT	biofilm attached cellular metabolic activity
BVA	biofilm attached cellular viability
Ca	*Candida albicans*
Cap1	adenylate cyclase-associated proteins
*CAT1*	catalase A
Cd	*Candida dubliniensis*
CFU	colony-forming unit
CI	confidence interval
Ck	*Candida krusei*
CRT	classification and regression tree
Ct	*Candida tropicalis*
CTA4	cation-transporting P-type ATPase
CV	crystal violet
DC	dilution control
EC_10_	minimum effective concentration (10%)
EMMeans	estimated marginal means
EO	essential oil
FL	fluconazole
FWER	family-wise error rate
GC	growth control
*GPX1*	glutathione peroxidase 1
Hog1	high osmolarity glycerol
In-PMT	inverse planktonic metabolic activity
KMO	Kaiser–Meyer–Olkin test
L	lavender essential oil
MA	Massachusetts
MAPK	mitogen-activated protein kinase
MIC_90_	minimum inhibitory concentration (90%)
ML	machine learning
MN	menadione
MoA	mechanism of action
NAC	non-albicans Candida
NC	background noise control
OC	oxidative stress positive control
P	peppermint essential oil
PC	rotated principal component scores
PCA	principal component analysis
PMT	planktonic metabolic activity
PVA	planktonic viability
RAMEB	randomly methylated beta-cyclodextrin
RB	RAMEB-encapsulated lemon balm essential oil
RC	antibiotic reference controls
REML	restricted maximum likelihood
REO	RAMEB-encapsulated essential oil
RL	RAMEB-encapsulated lavender essential oil
RNS	reactive nitrogen species
ROS	reactive oxygen species
RP	RAMEB-encapsulated peppermint essential oil
RT	RAMEB-encapsulated thyme essential oil
SC	sterility control
*SOD1*	superoxide dismutase 1
T	thyme essential oil
TS	test samples/treatments
UBC	untreated biofilm control
UC	untreated control

## Appendix A

### Microplate Washing Conditions for Biofilm Assays

The automated washing of biofilm-containing microplates was performed using a BioTek ELx50 microplate washer. Washing parameters were optimized to ensure the effective removal of non-adherent cells and excess staining solution while preserving the integrity of adherent biofilms. Phosphate-buffered saline (PBS, pH 7.43) was used as the washing buffer for all steps. Washing cycles were performed using a gentle bottom-dispense mode to minimize shear stress on the biofilm surface. Each wash cycle consisted of controlled buffer dispensing followed by aspiration.

**Table A1:** The washer was configured with the following parameters.

Method	Component	Parameter
Wash	Number of cycles	2
	Wash format	Plate
	Soak	Yes
	Soak duration	3 s
Dispense	Dispense volume	280 µL/well
	Dispense flow rate	150 µL/well/second
	Bottom dispense	Yes
	Horizontal dispense position	1.006 mm
Aspiration	Aspiration height	5.08 mm
	Horizontal aspiration position	−1.006 mm
	Travel rate	5.0 mm/second
	Aspiration delay	100 milliseconds

Following washing, care was taken to avoid the complete drying of the wells prior to the subsequent fixation or staining steps. For the crystal violet assays, washing after staining was performed under identical conditions to remove unbound dye while maintaining the adherence of the stained biofilm biomass. These parameters were selected based on preliminary optimization to balance washing efficiency and biofilm preservation and were applied consistently across all the experiments.
