# Supplementary material for: Species-Specific Susceptibility of Planktonic and Biofilm Forming Candida Strains to Cyclodextrin-Encapsulated Essential Oils
Source: Pharmaceutics. 2026 Apr 20;18(4):508. doi: 10.3390/pharmaceutics18040508 (PMC13119200; doi:10.3390/pharmaceutics18040508)
Supplement: Supplementary file 1 [file pharmaceutics-18-00508-s001.zip › 02_Supplementary materials_proofread_20260414.pdf]

pharmaceutics

MDPI

Supplementary Materials

Species-specific susceptibility of planktonic and biofilm forming Candida strains to cyclodextrin-encapsulated essential oils

Sourav Das[1,2,†,*], Farid Baradarbarjastehbaf[2], Aliz Sára Szokolics[2], Génesis Katherine Dela Campos[3], Zoltán Gazdag[4], Aleksandar Széchenyi[2,5], Attila Miseta[6], Gábor L. Kovács[1,6], and Tamás Kőszegi[1,6,†]

[1] Molecular Medicine Research Group, Szentágothai Research Center, University of Pécs, Ifjúság u. 20, 7624 Pécs, Hungary; kovacs.l.gabor@pte.hu (G.L.K.); koszegi.tamas@pte.hu (T.K.)
[2] Institute of Pharmaceutical Technology and Biopharmacy, Faculty of Pharmacy, University of Pécs, Rókus u. 2., 7624 Pécs, Hungary; baradarbarjastehbaf.farid@pte.hu (F.B.); szokolics.aliz@edu.pte.hu (A.S.S.); szechenyi.aleksandar@gytk.pte.hu (A.S.)
[3] Institute of Biology, Faculty of Sciences, University of Pécs, Ifjúság u. 6, 7624 Pécs, Hungary; dela.campos.genesis@pte.hu (G.K.D.C.)
[4] Department of Molecular Biology and Microbiology, Institute of Biology, University of Pécs, Ifjúság u. 6, 7624 Pécs, Hungary; gazdag@gamma.ttk.pte.hu
[5] Faculty of Food Technology Osijek, University of Osijek, Franje Kuhaca 20, 31000 Osijek, Croatia
[6] Department of Laboratory Medicine, Medical School, University of Pécs, Ifjúság út 13, 7624 Pécs, Hungary; attila.miseta@aok.pte.hu
* Correspondence: sourav.das@pte.hu; Tel.: +36-20-2983254
† These authors contributed equally to this work.

 https://doi.org/10.3390/pharmaceutics18040508

Table S1. Consumables used throughout the studies

Consumables	Producer/distributor
Dihydrorhodamine 123<br>Dihydroethidium	Cayman Chemicals, Biomarker kft., Gödöllő, Hungary
Lavender oil<br>Lemon balm oil<br>Peppermint oil<br>Thyme oil	Panarom kft., Budapest, Hungary
Lavender oil-RAMEB *<br>Lemon balm oil-RAMEB *<br>Lavender oil-RAMEB *<br>Thyme oil-RAMEB *<br>Randomly methylated β-cyclodextrin	Cyclolab kft., Budapest, Hungary
Sodium chloride<br>0.22 μm vacuum filtration unit<br>Potassium chloride<br>Sodium phosphate dibasic<br>Resazurin sodium salt<br>3-Morpholinopropane sulfonic acid<br>Dimethyl sulfoxide<br>Menadione<br>Propidium iodide<br>SYBR Green I nucleic acid gel stain<br>Amphothericin B<br>Fluconazole<br>RPMI 1640 medium	Merck, Budapest, Hungary
Glacial acetic acid<br>Adenine<br>Agar-agar<br>Crystal violet<br>Dextrose<br>Peptone<br>Potassium phosphate monobasic<br>Yeast extract	Reanal Labor, Budapest, Hungary
Macherey-Nagel NucleoSpin RNA kit	AKTIVIT Kft., Budapest, Hungary

Nuclease-free molecular-grade water<br>High-Capacity cDNA reverse transcription kit	Fisher Scientific (Budapest, Hungary
96-well microtiter plates	SPL Life Sciences, Pocheon-si, Republic of Korea
96-well microtiter plates	Sarstedt AG & Co. KG, Nümbrecht, Germany
BioSyGreen Mix Hi-ROX (2×)	PCR Biosystems, London, UK

*Encapsulation efficiency (%EE) values were based on previously reported data, while loading capacity was not independently quantified in the present study [33].

Table S2. Oligonucleotide primers and amplicon sizes for quantitative real-time PCR (qPCR) analysis of Candida species genes.

This table lists the NCBI accession identification, forward and reverse primer sequences (5'-3'), and the expected amplicon size (in base pairs, bp) for each target gene (*CAT1*, *GPX1*, *SOD1*, and RDN18). References for primer sequences are also provided. Gene targets, accession IDs, primer pairs, expected amplicon sizes, and annealing temperatures used for *Candida albicans* (Ca), *Candida tropicalis* (Ct), *Candida dubliniensis* (Cd) and *Candida krusei* (Ck) PCR.

Gene	Species	Role	Accession identification/sequence	Forward primer (5'-->3')	Reverse primer (5'-->3')	Amplicon size (bp)	Ta* (°C)
CAT1	**Ca**	Model sequence	XM_713725.2	CGGTGCTGTTTTCAACCCAC	GTGGCAG-CAC-CATTCCAAAC	153	50
	**Ct**	Alignment sequence	CP047870.1, XM_002549860.1, AB181391.1, M18832.1, X06660.1, X13978.1	ACCATGACTGAA-GCTCAAGCTAA	TGGAATGGAG-TAGCAGCACC	455	56
	**Cd**	Alignment sequence	XM_002417253.1, FM992688.1, AB181389.1	CCCACACATGAGA-GACGGTG	TTGGATTGCAG-CATCAGCAC	280	56
	**Ck**	Alignment	LC510309.1, MF580721.1, CP021089.	TTGACTCGTTGGCG-CATTTC	AG-GTTTCCCTCCTCGGTGTA	256	58

		sequence	1, CP028774.1				
GPX1	**Ca**	Model sequence	XM_709203.2	ATGGTGAACAA-GCAGATCCAGT	TGCCAC-GGGTCTAG-TAAACG	155	51
	**Ct**	Alignment sequence	CP047872.1, CP047872.1, XM_002548604.1	CAWKTAAWTSTRGWYWYACTCCAMAAT	AMTKYTTCAAR-ATKYSRTTTMA	202	50
	**Cd**	Alignment sequence	XM_002420834.1, FM992693.1, XM_002420833.1, XM_002420832.1	TRGG-WTTCCCNTGTAAYCAAT	ATTCCAY-WTNAYTCKRK-KNRWNC	321	48
	**Ck**	Alignment sequence	NC_042508.1	GGCAAGGTT-GTTCTGATCGTCAA	ATTATCGG-GAAGGAGA-CACCATA	208	55
SOD1	**Ca**	Model sequence	XM_019475368.1	TGTCGCTGTT-GTCAGAGGTG	GGACCAG-CAGAAGTACAACCA	177	50
	**Ct**	Alignment sequence	CP047875.1, M55527.1, DQ515959.1, PX836787.1	CGACTGTTT-GGAAGGGATGT	CTTGGATGTGG-TAGCCGTTT	232	55
	**Cd**	Alignment sequence	FM992695.1, X99399.1, MW281616.1, MW281619.1, MW281617.1	AAACGGCTAC-CACATCCAAG	CCAA-GCCCAAGGTTCAACTA	249	55
	**Ck**	Alignment	PQ364113.1, PQ364112.	TAACGATA-CAGGGCCTTTGG	AG-GGCTAGCCAGAAGGAAAG	229	56

		sequence	1, XR_003834612.1, CP039617.1				
**RDN18**	**Ca**	Model sequence	XR_002086442.1	AATGCCTTCGGGCTCTTTGA	CCTT-GGATGTGG-TAGCCGTT	195	52
	**Ct**	Alignment sequence	XM_019475368.1, CP047871.1, XM_002545332.1	TKSYRMTGKTMCYC-AMKTCA	TTCTAM-MRYCWSCRK-KRYCR	326	52
	**Cd**	Alignment sequence	XM_002419841.1, FM992691.1	CRAATCCRCMYY-WACCAYAAT	AATCCRCMYY-WACCAYAATYWYYWG	255	50
	**Ck**	Alignment sequence	CP028773.1, CP028531.1, XM_029463928.1, CP133381.1, CP093506.1, CP039612.1, CP021090.1	CGA-GATCGTCTTCACCCGAG	TGGTT-GTACTTCTGCCGGTC	214	56

*Annealing temperature (Ta: °C)

Table S3. Minimum inhibitory concentration (MIC_{90}) raw data. All treatments are significantly different compared to growth control (*n*=6, p < .001).

Strain	Treatment	MIC_{90} (µg/mL, mean ± SE)
**Ca1372**	**AM**	0.241 ± 0.006 $^{III, IV, V, VI, VII, IX}$
	**FL**	1.867 ± 0.046 $^{III, IV, V, IX}$
	**L**	15.450 ± 0.249 $^{I, II, IV, V, VI, VII, VIII, IX, X}$
	**B**	7.567 ± 0.152 $^{I, II, III, V, VII, VIII, X}$
	**P**	30.131 ± 1.018 $^{I, II, III, IV, VI, VII, VIII, IX, X}$
	**T**	4.176 ± 0.184 $^{I, III, V}$
	**RL**	3.946 ± 0.085 $^{I, III, IV, V, IX}$
	**RB**	1.966 ± 0.052 $^{III, IV, V, IX}$
	**RP**	7.445 ± 0.160 $^{I, II, III, V, VII, VIII, X}$

	**RT**	0.959 ± 0.024 [III, IV, V, IX]
**Ca1423**	**AM**	0.230 ± 0.004 [II, III, IV, V, VI, VII, VIII, IX]
	**FL**	29.019 ± 0.524 [I, III, V, VI, VII, VIII, X]
	**L**	62.868 ± 1.093 [I, II, IV, VI, VII, VIII, IX, X]
	**B**	31.592 ± 0.705 [I, III, V, VI, VII, VIII, X]
	**P**	65.333 ± 1.594 [I, II, IV, VI, VII, VIII, IX, X]
	**T**	14.607 ± 0.595 [I, II, III, IV, V, VIII, IX, X]
	**RL**	15.491 ± 0.331 [I, II, III, IV, V, VIII, IX, X]
	**RB**	7.737 ± 0.334 [I, II, III, IV, V, VI, VII, IX, X]
	**RP**	29.738 ± 0.848 [I, III, V, VI, VII, VIII, X]
	**RT**	3.612 ± 0.220 [II, III, IV, V, VI, VII, VIII, IX]
**Ca1424**	**AM**	0.247 ± 0.012 [II, III, IV, V, VI, VII, VIII, IX, X]
	**FL**	31.771 ± 0.892 [I, III, V, VI, VII, VIII, X]
	**L**	62.236 ± 1.577 [I, II, IV, VI, VII, VIII, IX, X]
	**B**	29.995 ± 0.805 [I, III, V, VI, VII, VIII, IX, X]
	**P**	62.413 ± 1.491 [I, II, IV, VI, VII, VIII, IX, X]
	**T**	14.876 ± 0.535 [I, II, III, IV, V, VIII, IX, X]
	**RL**	14.973 ± 0.272 [I, II, III, IV, V, VIII, IX, X]
	**RB**	7.652 ± 0.207 [I, II, III, IV, V, VI, VII, IX, X]
	**RP**	33.483 ± 1.152 [I, III, IV, V, VI, VII, VIII, X]
	**RT**	3.813 ± 0.105 [I, II, III, IV, V, VI, VII, VIII, IX]
**Ct1368**	**AM**	0.226 ± 0.007 [II, III, IV, V, VI, VII, VIII, IX, X]
	**FL**	28.202 ± 1.387 [I, III, IV, V, IX, X]
	**L**	62.113 ± 1.281 [I, II, VI, VII, VIII, IX, X]
	**B**	61.819 ± 1.713 [I, II, VI, VII, VIII, X]
	**P**	61.525 ± 2.183 [I, II, VI, VII, VIII, X]
	**T**	31.607 ± 0.648 [I, III, IV, V, IX, X]
	**RL**	30.452 ± 0.492 [I, III, IV, V, IX, X]
	**RB**	29.932 ± 1.246 [I, III, IV, V, IX, X]
	**RP**	58.487 ± 2.522 [I, II, III, VI, VII, VIII, X]
	**RT**	16.018 ± 0.573 [I, II, III, IV, V, VI, VII, VIII, IX]
**Ct1432**	**AM**	0.231 ± 0.002 [III, IV, V, VI, VII, VIII, IX, X]
	**FL**	0.476 ± 0.008 [III, IV, V, VI, VII, VIII, IX]
	**L**	66.557 ± 2.479 [I, II, IV, V, VI, VII, VIII, IX, X]
	**B**	32.762 ± 1.943 [I, II, III, V, VI, VII, VIII, X]
	**P**	62.824 ± 1.521 [I, II, III, IV, VI, VII, VIII, IX, X]
	**T**	15.238 ± 0.296 [I, II, III, IV, V, VIII, IX, X]
	**RL**	14.572 ± 0.446 [I, II, III, IV, V, VIII, IX, X]
	**RB**	8.075 ± 0.211 [I, II, III, IV, V, VI, VII, IX, X]
	**RP**	31.359 ± 1.206 [I, II, III, V, VI, VII, VIII, X]
	**RT**	3.849 ± 0.173 [I, III, IV, V, VI, VII, VIII, IX]
**Ck[illegible]**	**AM**	0.218 ± 0.009 [II, III, IV, V, VI, VII, VIII, IX, X]

	**FL**	30.028 ± 1.156 $^{I, III, V, VI, VII, VIII, X}$
	**L**	62.432 ± 1.034 $^{I, II, IV, VI, VII, VIII, IX, X}$
	**B**	32.746 ± 0.917 $^{I, III, V, VI, VII, VIII, X}$
	**P**	65.235 ± 1.415 $^{I, II, IV, VI, VII, VIII, IX, X}$
	**T**	16.026 ± 0.715 $^{I, II, III, IV, V, VIII, IX, X}$
	**RL**	15.254 ± 0.365 $^{I, II, III, IV, V, VIII, IX, X}$
	**RB**	7.477 ± 0.410 $^{I, II, III, IV, V, VI, VII, IX, X}$
	**RP**	30.076 ± 1.000 $^{I, III, V, VI, VII, VIII, X}$
	**RT**	3.962 ± 0.087 $^{I, II, III, IV, V, VI, VII, VIII, IX}$
**Ck1447**	**AM**	0.108 ± 0.007 $^{II, III, IV, V, VI, VII, VIII, IX, X}$
	**FL**	14.681 ± 0.407 $^{I, III, IV, V, VI, VII, VIII, IX}$
	**L**	62.860 ± 2.289 $^{I, II, V, VI, VII, VIII, IX, X}$
	**B**	63.225 ± 1.692 $^{I, II, V, VI, VII, VIII, IX, X}$
	**P**	59.165 ± 1.765 $^{I, II, III, IV, VI, VII, VIII, X}$
	**T**	30.567 ± 0.697 $^{I, II, III, IV, V, IX, X}$
	**RL**	30.786 ± 0.623 $^{I, II, III, IV, V, IX, X}$
	**RB**	30.989 ± 0.345 $^{I, II, III, IV, V, IX, X}$
	**RP**	57.812 ± 3.113 $^{I, II, III, IV, VI, VII, VIII, X}$
	**RT**	14.245 ± 0.649 $^{I, III, IV, V, VI, VII, VIII, IX}$
**Cd1470**	**AM**	0.095 ± 0.002 $^{III, IV, V, VI, VII, VIII, IX, X}$
	**FL**	0.103 ± 0.004 $^{III, IV, V, VI, VII, VIII, IX, X}$
	**L**	46.715 ± 6.380 $^{I, II, IV, VI, VII, VIII, IX, X}$
	**B**	54.752 ± 6.284 $^{I, II, III, V, VI, VII, VIII, IX, X}$
	**P**	44.023 ± 3.698 $^{I, II, IV, VI, VIII, IX, X}$
	**T**	35.399 ± 3.488 $^{I, II, III, IV, V, VII, VIII, IX, X}$
	**RL**	43.276 ± 2.322 $^{I, II, III, IV, VI, VIII, IX, X}$
	**RB**	24.802 ± 2.702 $^{I, II, III, IV, V, VI, VII, IX, X}$
	**RP**	64.789 ± 2.747 $^{I, II, III, IV, V, VI, VII, VIII, X}$
	**RT**	17.725 ± 1.789 $^{I, II, III, IV, V, VI, VII, VIII, IX}$
**Cd1471**	**AM**	0.093 ± 0.004 $^{III, IV, V, VI, VII, VIII, IX, X}$
	**FL**	0.230 ± 0.009 $^{III, IV, V, VI, VII, VIII, IX, X}$
	**L**	61.357 ± 1.670 $^{I, II, VI, VII, VIII, IX, X}$
	**B**	59.536 ± 2.353 $^{I, II, V, VI, VII, VIII, IX, X}$
	**P**	63.745 ± 1.662 $^{I, II, IV, VI, VII, VIII, X}$
	**T**	29.979 ± 0.755 $^{I, II, III, IV, V, IX, X}$
	**RL**	31.100 ± 0.717 $^{I, II, III, IV, V, IX, X}$
	**RB**	30.176 ± 0.720 $^{I, II, III, IV, V, IX, X}$
	**RP**	65.249 ± 2.411 $^{I, II, III, IV, VI, VII, VIII, X}$
	**RT**	14.935 ± 0.797 $^{I, II, III, IV, V, VI, VII, VIII, IX}$

I: statistically significant ($p < .001$) when compared to AM
II: statistically significant ($p < .001$) when compared to FL
III: statistically significant ($p < .001$) when compared to L

IV: statistically significant (p < .001) when compared to B
V: statistically significant (p < .001) when compared to P
VI: statistically significant (p < .001) when compared to T
VII: statistically significant (p < .001) when compared to RL
VIII: statistically significant (p < .001) when compared to RB
IX: statistically significant (p < .001) when compared to RP
X: statistically significant (p < .001) when compared to RT

Table S4. Minimum effective concentration (EC_{10}) raw data. All treatments are significantly different compared to growth control (*n*=6, p < .001).

Strain	Treatment	EC_{10} (µg/mL, mean ± SE)
Ca1372	AM	0.067 ± 0.002 [III, IV, V, VI, VII, IX]
	FL	0.490 ± 0.011 [III, IV, V, VI, IX]
	L	4.583 ± 0.079 [I, II, IV, V, VI, VII, VIII, IX, X]
	B	2.575 ± 0.086 [I, II, III, V, VI, VII, VIII, X]
	P	9.664 ± 0.328 [I, II, III, IV, VI, VII, VIII, IX, X]
	T	1.199 ± 0.016 [I, II, III, IV, V, IX, X]
	RL	0.941 ± 0.039 [I, III, IV, V, IX, X]
	RB	0.654 ± 0.013 [III, IV, V, IX]
	RP	2.119 ± 0.033 [I, II, III, V, VI, VII, VIII, X]
	RT	0.228 ± 0.005 [III, IV, V, VI, VII, IX]
Ca1423	AM	0.074 ± 0.002 [II, III, IV, V, VI, VII, VIII, IX, X]
	FL	8.082 ± 0.270 [I, III, V, VI, VII, VIII, IX, X]
	L	16.343 ± 0.722 [I, II, IV, V, VI, VII, VIII, IX, X]
	B	7.669 ± 0.141 [I, III, V, VI, VII, VIII, IX, X]
	P	13.943 ± 0.240 [I, II, III, IV, VI, VII, VIII, IX, X]
	T	4.615 ± 0.156 [I, II, III, IV, V, VIII, IX, X]
	RL	4.221 ± 0.080 [I, II, III, IV, V, VIII, IX, X]
	RB	2.430 ± 0.076 [I, II, III, IV, V, VI, VII, IX, X]
	RP	10.330 ± 0.298 [I, II, III, IV, V, VI, VII, VIII, X]
	RT	0.864 ± 0.019 [I, II, III, IV, V, VI, VII, VIII, IX]
Ca1424	AM	0.071 ± 0.002 [II, III, IV, V, VI, VII, VIII, IX, X]
	FL	7.601 ± 0.134 [I, III, V, VI, VII, VIII, IX, X]
	L	16.612 ± 0.525 [I, II, IV, V, VI, VII, VIII, IX, X]
	B	7.997 ± 0.386 [I, III, V, VI, VII, VIII, IX, X]
	P	15.826 ± 0.614 [I, II, III, IV, VI, VII, VIII, IX, X]
	T	4.128 ± 0.045 [I, II, III, IV, V, VIII, IX, X]
	RL	4.688 ± 0.191 [I, II, III, IV, V, VIII, IX, X]
	RB	1.823 ± 0.066 [I, II, III, IV, V, VI, VII, IX, X]
	RP	9.747 ± 0.408 [I, II, III, IV, V, VI, VII, VIII, X]
	RT	1.097 ± 0.020 [I, II, III, IV, V, VI, VII, VIII, IX]
Ct1368	AM	0.062 ± 0.001 [II, III, IV, V, VI, VII, VIII, IX, X]
	FL	7.749 ± 0.130 [I, III, IV, V, VII, IX, X]

	**L**	20.719 ± 0.448 [I, II, IV, V, VI, VII, VIII, IX, X]
	**B**	16.934 ± 0.545 [I, II, III, VI, VII, VIII, IX, X]
	**P**	16.919 ± 0.559 [I, II, III, VI, VII, VIII, IX, X]
	**T**	8.158 ± 0.126 [I, III, IV, V, VII, IX, X]
	**RL**	9.667 ± 0.176 [I, II, III, IV, V, VI, VIII, IX, X]
	**RB**	7.748 ± 0.148 [I, III, IV, V, VII, IX, X]
	**RP**	14.673 ± 0.398 [I, II, III, IV, V, VI, VII, VIII, X]
	**RT**	4.066 ± 0.075 [I, II, III, IV, V, VI, VII, VIII, IX]
**Ct1432**	**AM**	0.061 ± 0.002 [II, III, IV, V, VI, VII, VIII, IX, X]
	**FL**	7.304 ± 0.111 [I, III, IV, V, VI, VII, VIII, IX, X]
	**L**	15.204 ± 0.497 [I, II, IV, V, VI, VII, VIII, IX, X]
	**B**	20.076 ± 0.435 [I, II, III, V, VI, VII, VIII, IX, X]
	**P**	16.871 ± 0.257 [I, II, III, IV, VI, VII, VIII, X]
	**T**	9.381 ± 0.280 [I, II, III, IV, V, VII, IX, X]
	**RL**	8.624 ± 0.165 [I, II, III, IV, V, VI, IX, X]
	**RB**	8.907 ± 0.170 [I, II, III, IV, V, IX, X]
	**RP**	17.213 ± 0.508 [I, II, III, IV, VI, VII, VIII, X]
	**RT**	5.121 ± 0.124 [I, II, III, IV, V, VI, VII, VIII, IX]
**Ck779**	**AM**	0.062 ± 0.002 [III, IV, V, VI, VII, VIII, IX, X]
	**FL**	0.113 ± 0.002 [III, IV, V, VI, VII, VIII, IX, X]
	**L**	19.208 ± 0.569 [I, II, IV, V, VI, VII, VIII, IX, X]
	**B**	9.555 ± 0.224 [I, II, III, V, VI, VII, VIII, IX, X]
	**P**	15.205 ± 0.537 [I, II, III, IV, VI, VII, VIII, IX, X]
	**T**	4.336 ± 0.112 [I, II, III, IV, V, VIII, IX, X]
	**RL**	4.119 ± 0.075 [I, II, III, IV, V, VIII, IX, X]
	**RB**	2.037 ± 0.044 [I, II, III, IV, V, VI, VII, IX, X]
	**RP**	7.168 ± 0.153 [I, II, III, IV, V, VI, VII, VIII, X]
	**RT**	0.964 ± 0.021 [I, II, III, IV, V, VI, VII, VIII, IX]
**Ck1447**	**AM**	0.061 ± 0.001 [II, III, IV, V, VI, VII, VIII, IX, X]
	**FL**	9.856 ± 0.167 [I, III, IV, V, VI, VII, VIII, IX, X]
	**L**	14.190 ± 0.547 [I, II, IV, V, VI, VII, VIII, IX, X]
	**B**	8.557 ± 0.250 [I, II, III, V, VI, VII, VIII, IX, X]
	**P**	18.995 ± 0.508 [I, II, III, IV, VI, VII, VIII, IX, X]
	**T**	3.720 ± 0.181 [I, II, III, IV, V, VIII, IX, X]
	**RL**	4.024 ± 0.100 [I, II, III, IV, V, VIII, IX, X]
	**RB**	2.252 ± 0.079 [I, II, III, IV, V, VI, VII, IX, X]
	**RP**	7.378 ± 0.399 [I, II, III, IV, V, VI, VII, VIII, X]
	**RT**	1.210 ± 0.033 [I, II, III, IV, V, VI, VII, VIII, IX]
**Cd1470**	**AM**	0.025 ± 0.001 [II, III, IV, V, VI, VII, VIII, IX, X]
	**FL**	3.419 ± 0.080 [I, III, IV, V, VI, VII, VIII, IX]
	**L**	20.578 ± 0.500 [I, II, IV, V, VI, VII, VIII, IX, X]
	**B**	19.292 ± 0.650 [I, II, III, V, VI, VII, VIII, IX, X]

	**P**	16.249 ± 0.535 $^{I, II, III, IV, VI, VII, VIII, IX, X}$
	**T**	7.711 ± 0.230 $^{I, II, III, IV, V, VIII, IX, X}$
	**RL**	7.642 ± 0.148 $^{I, II, III, IV, V, VIII, IX, X}$
	**RB**	6.686 ± 0.213 $^{I, II, III, IV, V, VI, VII, IX, X}$
	**RP**	17.847 ± 0.475 $^{I, II, III, IV, V, VI, VII, VIII, X}$
	**RT**	4.042 ± 0.093 $^{I, III, IV, V, VI, VII, VIII, IX}$
**Cd1471**	**AM**	0.029 ± 0.001 $^{III, IV, V, VI, VII, VIII, IX, X}$
	**FL**	0.063 ± 0.004 $^{III, IV, V, VI, VII, VIII, IX, X}$
	**L**	16.356 ± 0.215 $^{I, II, IV, V, VI, VII, VIII, IX, X}$
	**B**	17.691 ± 0.422 $^{I, II, III, V, VI, VII, VIII, IX, X}$
	**P**	20.439 ± 0.442 $^{I, II, III, IV, VI, VII, VIII, X}$
	**T**	6.621 ± 0.292 $^{I, II, III, IV, V, VII, VIII, IX, X}$
	**RL**	9.798 ± 0.498 $^{I, II, III, IV, V, VI, IX, X}$
	**RB**	9.641 ± 0.185 $^{I, II, III, IV, V, VI, IX, X}$
	**RP**	20.847 ± 0.673 $^{I, II, III, IV, VI, VII, VIII, X}$
	**RT**	1.799 ± 0.061 $^{I, II, III, IV, V, VI, VII, VIII, IX}$

I: statistically significant (p < .001) when compared to AM
II: statistically significant (p < .001) when compared to FL
III: statistically significant (p < .001) when compared to L
IV: statistically significant (p < .001) when compared to B
V: statistically significant (p < .001) when compared to P
VI: statistically significant (p < .001) when compared to T
VII: statistically significant (p < .001) when compared to RL
VIII: statistically significant (p < .001) when compared to RB
IX: statistically significant (p < .001) when compared to RP
X: statistically significant (p < .001) when compared to RT

Table S5. Reactive nitrogen species (RNS) and reactive oxygen species (ROS) generation in different Candida strains. All treatments are significantly different compared to untreated control (UC, *n*=6, p < .001).

**Strain**	**Treatment**	**%increment compared to UC**	
		**RNS (mean ± SE)**	**ROS (mean ± SE)**
**Ca1372**	**AM**	154.958 ± 2.588 $^{III, IV, V, VI, VII, IX}$	188.531 ± 4.137 $^{II, III, IV, V, VI, VII, VIII, IX, X}$
	**FL**	157.031 ± 2.665 $^{III, IV, V, VI, VII, IX}$	108.601 ± 5.025 $^{I, X}$
	**L**	96.431 ± 3.880 $^{I, II, IV, VI, VII, VIII, IX, X}$	102.285 ± 3.402 $^{I, X}$
	**B**	114.111 ± 2.915 $^{I, II, III, V, VI, VII, VIII, X}$	101.125 ± 3.058 $^{I, X}$
	**P**	93.693 ± 2.624 $^{I, II, IV, VI, VII, VIII, IX, X}$	99.674 ± 3.004 $^{I, X}$
	**T**	136.193 ± 3.115 $^{I, II, III, IV, V, VIII, IX, X}$	100.963 ± 3.530 $^{I, X}$
	**RL**	142.656 ± 3.389 $^{I, II, III, IV, V, IX, X}$	102.040 ± 2.245 $^{I, X}$
	**RB**	153.618 ± 3.551 $^{III, IV, V, VI, IX}$	100.334 ± 1.783 $^{I, X}$

	**RP**	117.837 ± 2.878 $^{I, II, III, V, VI, VII, VIII, X}$	104.151 ± 1.177 $^{I, X}$
	**RT**	155.300 ± 2.417 $^{III, IV, V, VI, VII, IX}$	143.533 ± 1.885 $^{I, II, III, IV, V, VI, VII, VIII, IX}$
	**MN**	272.620 ± 12.362 $^{I, II, III, IV, V, VI, VII, VIII, IX, X}$	309.466 ± 14.567 $^{I, II, III, IV, V, VI, VII, VIII, IX, X}$
**Ca1423**	**AM**	155.259 ± 3.353 $^{II, III, IV, V, VI, VII, VIII, IX}$	184.253 ± 2.109 $^{II, III, IV, V, VI, VII, VIII, IX, X}$
	**FL**	95.933 ± 2.786 $^{I, VIII, X}$	102.380 ± 3.428 ^{I}
	**L**	95.401 ± 1.803 $^{I, VIII, X}$	101.990 ± 3.125 ^{I}
	**B**	93.746 ± 2.650 $^{I, VIII, X}$	99.831 ± 2.850 ^{I}
	**P**	95.953 ± 2.877 $^{I, VIII, X}$	100.577 ± 3.057 ^{I}
	**T**	97.638 ± 2.166 $^{I, VIII, X}$	99.112 ± 4.339 ^{I}
	**RL**	97.064 ± 2.574 $^{I, VIII, X}$	101.422 ± 4.258 ^{I}
	**RB**	113.411 ± 3.095 $^{I, II, III, IV, V, VI, VII, IX, X}$	99.781 ± 4.263 ^{I}
	**RP**	93.378 ± 2.966 $^{I, VIII, X}$	101.598 ± 3.630 ^{I}
	**RT**	145.894 ± 2.610 $^{II, III, IV, V, VI, VII, VIII, IX}$	99.765 ± 3.158 ^{I}
	**MN**	282.236 ± 22.352 $^{I, II, III, IV, V, VI, VII, VIII, IX, X}$	338.165 ± 21.737 $^{I, II, III, IV, V, VI, VII, VIII, IX, X}$
**Ca1424**	**AM**	157.004 ± 1.756 $^{II, III, IV, V, VI, VII, VIII, IX, X}$	190.070 ± 3.048 $^{II, III, IV, V, VI, VII, VIII, IX, X}$
	**FL**	95.964 ± 3.474 $^{I, VIII, X}$	102.434 ± 3.337 ^{I}
	**L**	94.799 ± 3.118 $^{I, VIII, X}$	99.728 ± 3.697 ^{I}
	**B**	93.984 ± 2.934 $^{I, VIII, X}$	100.960 ± 2.978 ^{I}
	**P**	93.512 ± 3.083 $^{I, VIII, X}$	100.358 ± 4.113 ^{I}
	**T**	96.706 ± 2.600 $^{I, VIII, X}$	98.581 ± 4.367 ^{I}
	**RL**	93.536 ± 3.029 $^{I, VIII, X}$	98.712 ± 2.607 ^{I}
	**RB**	122.822 ± 2.784 $^{I, II, III, IV, V, VI, VII, IX, X}$	97.702 ± 2.411 ^{I}
	**RP**	95.686 ± 3.111 $^{I, VIII, X}$	100.983 ± 4.400 ^{I}
	**RT**	136.722 ± 3.446 $^{I, II, III, IV, V, VI, VII, VIII, IX}$	99.679 ± 2.806 ^{I}
	**MN**	283.787 ± 16.646 $^{I, II, III, IV, V, VI, VII, VIII, IX, X}$	335.953 ± 16.987 $^{I, II, III, IV, V, VI, VII, VIII, IX, X}$
**Ct1368**	**AM**	157.707 ± 2.799 $^{II, III, IV, V, VI, VII, VIII, IX, X}$	193.575 ± 3.522 $^{II, III, IV, V, VI, VII, VIII, IX, X}$
	**FL**	95.904 ± 2.676 ^{I}	101.120 ± 3.894 ^{I}
	**L**	92.018 ± 1.581 ^{I}	100.324 ± 3.344 ^{I}
	**B**	94.231 ± 3.408 ^{I}	100.832 ± 3.557 ^{I}
	**P**	95.235 ± 2.739 ^{I}	99.912 ± 4.170 ^{I}
	**T**	94.189 ± 2.337 ^{I}	100.518 ± 2.736 ^{I}
	**RL**	93.789 ± 3.105 ^{I}	99.315 ± 3.109 ^{I}
	**RB**	93.233 ± 3.189 ^{I}	99.416 ± 2.675 ^{I}
	**RP**	95.264 ± 2.279 ^{I}	97.098 ± 1.796 ^{I}
	**RT**	100.041 ± 3.517 ^{I}	99.469 ± 3.407 ^{I}
	**MN**	276.653 ± 16.895 $^{I, II, III, IV, V, VI, VII, VIII, IX, X}$	338.896 ± 15.621 $^{I, II, III, IV, V, VI, VII, VIII, IX, X}$
**Ct1432**	**AM**	157.288 ± 2.636 $^{II, III, IV, V, VI, VII, VIII, IX, X}$	191.872 ± 2.828 $^{II, III, IV, V, VI, VII, VIII, IX, X}$
	**FL**	91.406 ± 0.916 ^{I}	99.930 ± 4.045 ^{I}

	**L**	96.918 ± 2.377 [I]	99.438 ± 3.813 [I]
	**B**	95.508 ± 2.730 [I]	99.941 ± 3.051 [I]
	**P**	93.646 ± 3.832 [I]	99.370 ± 2.404 [I]
	**T**	94.256 ± 3.412 [I]	97.952 ± 3.499 [I]
	**RL**	96.426 ± 3.404 [I]	99.524 ± 3.637 [I]
	**RB**	93.300 ± 2.468 [I]	100.835 ± 3.569 [I]
	**RP**	93.523 ± 3.137 [I]	99.959 ± 3.517 [I]
	**RT**	96.056 ± 3.002 [I]	99.028 ± 4.636 [I]
	**MN**	273.260 ± 11.001 [I, II, III, IV, V, VI, VII, VIII, IX, X]	344.456 ± 13.768 [I, II, III, IV, V, VI, VII, VIII, IX, X]
**Ck779**	**AM**	155.771 ± 3.225 [III, IV, V, VI, VII, VIII, IX, X]	192.599 ± 2.978 [II, III, IV, V, VI, VII, VIII, IX, X]
	**FL**	155.990 ± 2.107 [III, IV, V, VI, VII, VIII, IX, X]	169.169 ± 5.258 [I, III, IV, V, VI, VII, VIII, IX, X]
	**L**	94.589 ± 1.800 [I, II, VIII, X]	100.152 ± 4.410 [I, II]
	**B**	96.181 ± 3.648 [I, II, VIII, X]	99.159 ± 2.399 [I, II]
	**P**	95.933 ± 2.066 [I, II, VIII, X]	99.831 ± 3.186 [I, II]
	**T**	97.055 ± 3.720 [I, II, VIII, X]	101.264 ± 3.774 [I, II]
	**RL**	96.826 ± 2.770 [I, II, VIII, X]	100.734 ± 1.465 [I, II]
	**RB**	119.437 ± 3.796 [I, II, III, IV, V, VI, VII, IX, X]	98.669 ± 2.944 [I, II]
	**RP**	98.298 ± 2.206 [I, II, VIII, X]	98.637 ± 3.482 [I, II]
	**RT**	143.987 ± 2.060 [I, II, III, IV, V, VI, VII, VIII, IX]	99.248 ± 3.601 [I, II]
	**MN**	279.340 ± 18.200 [I, II, III, IV, V, VI, VII, VIII, IX, X]	335.342 ± 19.259 [I, II, III, IV, V, VI, VII, VIII, IX, X]
**Ck1447**	**AM**	157.167 ± 2.750 [II, III, IV, V, VI, VII, VIII, IX, X]	193.650 ± 2.847 [II, III, IV, V, VI, VII, VIII, IX, X]
	**FL**	96.430 ± 2.030 [I, VIII, X]	99.502 ± 3.490 [I]
	**L**	96.095 ± 2.347 [I, VIII, X]	100.815 ± 3.263 [I]
	**B**	94.377 ± 3.116 [I, VIII, X]	101.192 ± 3.578 [I]
	**P**	97.306 ± 2.256 [I, VIII, X]	100.670 ± 4.129 [I]
	**T**	103.109 ± 1.802 [I, VIII, X]	103.111 ± 2.749 [I]
	**RL**	98.764 ± 3.200 [I, VIII, X]	98.502 ± 1.871 [I]
	**RB**	114.820 ± 3.454 [I, II, III, IV, V, VI, VII, IX, X]	98.721 ± 3.175 [I]
	**RP**	94.663 ± 2.389 [I, VIII, X]	101.576 ± 3.475 [I]
	**RT**	134.077 ± 1.942 [I, II, III, IV, V, VI, VII, VIII, IX]	99.605 ± 2.592 [I]
	**MN**	286.294 ± 13.327 [I, II, III, IV, V, VI, VII, VIII, IX, X]	327.619 ± 16.596 [I, II, III, IV, V, VI, VII, VIII, IX, X]
**Cd1470**	**AM**	154.928 ± 1.794 [II, III, IV, V, VI, VII, VIII, IX, X]	221.684 ± 2.764 [II, III, IV, V, VI, VII, VIII, IX, X]
	**FL**	102.949 ± 3.263 [I]	100.366 ± 5.071 [I]
	**L**	95.049 ± 2.255 [I]	99.374 ± 2.908 [I]
	**B**	95.604 ± 2.863 [I]	100.191 ± 4.690 [I]
	**P**	95.433 ± 3.513 [I]	100.178 ± 3.799 [I]
	**T**	94.628 ± 2.859 [I]	98.955 ± 4.461 [I]
	**RL**	93.400 ± 2.987 [I]	100.528 ± 3.758 [I]

	**RB**	93.267 ± 3.175 [I]	100.579 ± 3.855 [I]
	**RP**	96.341 ± 1.755 [I]	102.653 ± 1.804 [I]
	**RT**	98.273 ± 3.180 [I]	100.207 ± 3.162 [I]
	**MN**	273.821 ± 13.698 [I, II, III, IV, V, VI, VII, VIII, IX, X]	341.157 ± 12.062 [I, II, III, IV, V, VI, VII, VIII, IX, X]
**Cd1471**	**AM**	157.176 ± 3.415 [III, IV, V, VI, VII, VIII, IX, X]	221.202 ± 2.160 [II, III, IV, V, VI, VII, VIII, IX, X]
	**FL**	156.871 ± 4.116 [III, IV, V, VI, VII, VIII, IX, X]	190.540 ± 4.576 [I, III, IV, V, VI, VII, VIII, IX, X]
	**L**	94.078 ± 3.063 [I, II, X]	99.483 ± 2.710 [I, II]
	**B**	93.823 ± 2.463 [I, II, X]	101.265 ± 3.562 [I, II]
	**P**	95.605 ± 2.885 [I, II, X]	99.814 ± 4.005 [I, II]
	**T**	95.511 ± 2.928 [I, II, X]	102.071 ± 2.860 [I, II]
	**RL**	96.308 ± 2.686 [I, II, X]	100.975 ± 4.463 [I, II]
	**RB**	94.656 ± 4.018 [I, II, X]	101.445 ± 4.086 [I, II]
	**RP**	96.991 ± 3.141 [I, II, X]	101.510 ± 4.231 [I, II]
	**RT**	121.537 ± 2.229 [I, II, III, IV, V, VI, VII, VIII, IX]	101.544 ± 2.626 [I, II]
	**MN**	291.786 ± 8.775 [I, II, III, IV, V, VI, VII, VIII, IX, X]	330.806 ± 14.904 [I, II, III, IV, V, VI, VII, VIII, IX, X]

I: statistically significant (p < .001) when compared to AM
II: statistically significant (p < .001) when compared to FL
III: statistically significant (p < .001) when compared to L
IV: statistically significant (p < .001) when compared to B
V: statistically significant (p < .001) when compared to P
VI: statistically significant (p < .001) when compared to T
VII: statistically significant (p < .001) when compared to RL
VIII: statistically significant (p < .001) when compared to RB
IX: statistically significant (p < .001) when compared to RP
X: statistically significant (p < .001) when compared to RT

Table S6. Reduced percentage in planktonic metabolism (PMT) and viability (PVA) compared to untreated control (UC). All treatments are significantly different compared to untreated control (UC, *n*=6, p < .001).

Strain	Treat-ment	Duration (h)	%PMT (mean ± SE)	%PVA (mean ± SE)
**Ca1372**	**AM**	0	109.163 ± 2.497	99.288 ± 0.793
		2	98.078 ± 0.965 [IX, X]	98.782 ± 1.427 [IX, X]
		4	95.857 ± 1.013 [IX, X]	99.537 ± 0.895 [IX, X]
		8	94.080 ± 1.421 [IX, X]	97.185 ± 1.697 [IX, X]
		16	88.796 ± 1.416 [V, IX, X]	94.434 ± 1.912
	**FL**	0	109.648 ± 0.906	99.405 ± 0.678 [IX, X]
		2	98.083 ± 1.279 [IX, X]	99.479 ± 1.146 [IX, X]
		4	96.167 ± 1.464 [IX, X]	99.640 ± 0.539 [IX, X]

	8	94.043 ± 1.046 [IX, X]	96.811 ± 0.928 [IX, X]
	16	89.539 ± 1.524 [IX, X]	95.114 ± 1.128 [IX, X]
**L**	0	108.452 ± 2.618	99.272 ± 0.645
	2	98.016 ± 1.155 [IX, X]	98.279 ± 1.606 [IX, X]
	4	96.595 ± 1.028 [IX, X]	99.390 ± 1.038 [IX, X]
	8	95.226 ± 1.050 [IX, X]	98.107 ± 1.168 [IX, X]
	16	91.462 ± 1.009 [IX, X]	94.897 ± 1.104 [IX, X]
**B**	0	108.895 ± 2.190	99.307 ± 0.800
	2	99.569 ± 0.747 [IX, X]	99.356 ± 0.766 [IX, X]
	4	97.012 ± 1.436 [IX, X]	102.268 ± 2.035 [IX, X]
	8	95.508 ± 0.968 [IX, X]	97.134 ± 0.989 [IX, X]
	16	91.244 ± 1.685 [IX, X]	95.345 ± 1.669 [IX, X]
**P**	0	110.083 ± 2.537	99.817 ± 0.301
	2	98.141 ± 1.142 [IX, X]	99.766 ± 1.141 [I, IX, X]
	4	96.830 ± 1.479 [IX, X]	99.020 ± 1.249 [IX, X]
	8	95.405 ± 1.200 [IX, X]	97.531 ± 0.945 [IX, X]
	16	92.259 ± 0.770 [I, IX, X]	95.551 ± 0.624 [IX, X]
**T**	0	109.396 ± 1.510	99.938 ± 0.151
	2	98.823 ± 0.968 [IX, X]	99.276 ± 1.395 [IX, X]
	4	97.113 ± 1.441 [IX, X]	99.592 ± 0.638 [IX, X]
	8	94.562 ± 1.502 [IX, X]	96.441 ± 1.105 [IX, X]
	16	91.538 ± 1.424 [IX, X]	95.749 ± 1.168 [IX, X]
**RL**	0	110.693 ± 1.448	99.009 ± 0.878
	2	98.436 ± 1.764 [IX, X]	99.102 ± 0.562 [IX, X]
	4	97.934 ± 1.086 [IX, X]	99.494 ± 0.545 [IX, X]
	8	95.446 ± 0.966 [IX, X]	97.961 ± 1.783 [IX, X]
	16	91.432 ± 0.622 [IX, X]	94.523 ± 0.844 [IX, X]
**RB**	0	110.243 ± 1.688	99.512 ± 0.594
	2	99.246 ± 0.886 [IX, X]	99.273 ± 0.486 [IX, X]
	4	96.554 ± 0.964 [IX, X]	99.587 ± 0.811 [IX, X]
	8	94.873 ± 1.297 [IX, X]	97.302 ± 1.318 [IX, X]
	16	91.837 ± 0.998 [IX, X]	94.485 ± 1.183 [IX, X]
**RP**	0	111.339 ± 2.882	100.242 ± 0.910
	2	78.261 ± 1.305 [I, II, III, IV, V, VI, VII, VIII, X]	61.169 ± 1.567 [I, II, III, IV, V, VI, VII, VIII, X]
	4	75.730 ± 1.581 [I, II, III, IV, V, VI, VII, VIII, X]	78.968 ± 0.664 [I, II, III, IV, V, VI, VII, VIII, X]
	8	73.319 ± 1.551 [I, II, III, IV, V, VI, VII, VIII, X]	77.232 ± 0.586 [I, II, III, IV, V, VI, VII, VIII, X]
	16	69.779 ± 0.652 [I, II, III, IV, V, VI, VII, VIII, X]	74.044 ± 1.584 [I, II, III, IV, V, VI, VII, VIII, X]
**RT**	0	108.381 ± 2.779	100.398 ± 1.448
	2	67.014 ± 0.824 [I, II, III, IV, V, VI, VII, VIII, IX]	61.950 ± 0.935 [I, II, III, IV, V, VI, VII, VIII, IX]

		4	65.415 ± 0.556 [I, II, III, IV, V, VI, VII, VIII, IX]	50.246 ± 1.405 [I, II, III, IV, V, VI, VII, VIII, IX]
		8	63.687 ± 1.189 [I, II, III, IV, V, VI, VII, VIII, IX]	47.364 ± 0.790 [I, II, III, IV, V, VI, VII, VIII, IX]
		16	59.782 ± 1.193 [I, II, III, IV, V, VI, VII, VIII, IX]	44.508 ± 1.643 [I, II, III, IV, V, VI, VII, VIII, IX]
	**MN**	0	109.271 ± 2.781	106.724 ± 2.303
		2	75.053 ± 2.422 [I, II, III, IV, V, VI, VII, VIII, X]	65.996 ± 1.281 [I, II, III, IV, V, VI, VII, VIII, IX, X]
		4	72.048 ± 1.042 [I, II, III, IV, V, VI, VII, VIII, IX, X]	54.018 ± 3.011 [I, II, III, IV, V, VI, VII, VIII, IX, X]
		8	69.550 ± 1.497 [I, II, III, IV, V, VI, VII, VIII, IX, X]	52.484 ± 1.424 [I, II, III, IV, V, VI, VII, VIII, IX, X]
		16	66.596 ± 1.693 [I, II, III, IV, V, VI, VII, VIII, X]	48.864 ± 1.255 [I, II, III, IV, V, VI, VII, VIII, IX, X]
**Ca1423**	**AM**	0	110.991 ± 2.434	106.706 ± 0.537
		2	97.729 ± 1.528 [IX, X]	88.368 ± 0.866 [II, IV, IX, X]
		4	95.725 ± 1.063 [IX, X]	81.111 ± 1.693 [III, V, IX, X]
		8	93.592 ± 1.517 [IX, X]	75.408 ± 0.997 [IX, X]
		16	89.425 ± 1.119 [IX, X]	71.703 ± 1.290 [IX, X]
	**FL**	0	108.389 ± 2.670	107.042 ± 1.217
		2	99.082 ± 1.157 [IX, X]	91.175 ± 0.703 [I, IX, X]
		4	97.549 ± 1.546 [IX, X]	89.472 ± 1.876 [IX, X]
		8	95.188 ± 1.099 [IX, X]	84.336 ± 0.747 [IX, X]
		16	91.444 ± 0.674 [IX, X]	86.604 ± 1.612 [IX, X]
	**L**	0	108.993 ± 2.870	106.977 ± 0.943
		2	98.853 ± 1.299 [IX, X]	90.300 ± 0.896 [IX, X]
		4	98.345 ± 1.131 [IX, X]	89.139 ± 1.023 [I, IX, X]
		8	95.680 ± 0.650 [IX, X]	88.952 ± 0.923 [IX, X]
		16	92.065 ± 1.267 [IX, X]	86.212 ± 0.970 [IX, X]
	**B**	0	109.095 ± 2.410	98.336 ± 0.715
		2	99.465 ± 0.645 [IX, X]	91.207 ± 1.419 [I, IX, X]
		4	97.536 ± 1.387 [IX, X]	93.036 ± 1.459 [IX, X]
		8	95.158 ± 1.341 [IX, X]	89.598 ± 0.985 [IX, X]
		16	91.296 ± 1.350 [IX, X]	87.144 ± 1.616 [IX, X]
	**P**	0	109.733 ± 1.850	97.137 ± 1.401
		2	98.299 ± 1.325 [IX, X]	91.078 ± 1.214 [IX, X]
		4	97.310 ± 1.267 [IX, X]	94.531 ± 1.327 [I, IX, X]
		8	95.568 ± 1.259 [IX, X]	89.250 ± 1.129 [IX, X]
		16	92.670 ± 0.850 [IX, X]	86.571 ± 1.622 [IX, X]
	**T**	0	109.814 ± 1.988	97.811 ± 1.640
		2	98.691 ± 0.888 [IX, X]	90.727 ± 0.895 [IX, X]
		4	97.690 ± 1.184 [IX, X]	83.474 ± 1.549 [IX, X]
		8	94.741 ± 1.402 [IX, X]	99.679 ± 0.636 [IX, X]

		16	92.194 ± 1.242 [IX, X]	96.077 ± 0.621 [IX, X]
	**RL**	0	109.626 ± 1.515	107.401 ± 1.521
		2	98.464 ± 1.279 [IX, X]	90.140 ± 0.942 [IX, X]
		4	96.782 ± 1.208 [IX, X]	83.733 ± 1.058 [IX, X]
		8	94.943 ± 1.069 [IX, X]	99.398 ± 0.729 [IX, X]
		16	90.972 ± 0.903 [IX, X]	96.208 ± 1.184 [IX, X]
	**RB**	0	111.767 ± 1.745 [II]	102.943 ± 1.099
		2	98.036 ± 1.040 [IX, X]	88.508 ± 1.097 [IX, X]
		4	96.847 ± 0.666 [IX, X]	82.058 ± 1.585 [IX, X]
		8	95.220 ± 1.312 [IX, X]	99.549 ± 0.497 [IX, X]
		16	92.375 ± 1.012 [IX, X]	96.973 ± 0.880 [IX, X]
	**RP**	0	109.245 ± 1.243	107.411 ± 1.713
		2	77.951 ± 1.203 [I, II, III, IV, V, VI, VII, VIII, X]	69.857 ± 0.717 [I, II, III, IV, V, VI, VII, VIII, X]
		4	77.144 ± 1.126 [I, II, III, IV, V, VI, VII, VIII, X]	62.680 ± 1.176 [I, II, III, IV, V, VI, VII, VIII, X]
		8	73.100 ± 1.447 [I, II, III, IV, V, VI, VII, VIII, X]	79.581 ± 0.947 [I, II, III, IV, V, VI, VII, VIII, X]
		16	69.337 ± 1.310 [I, II, III, IV, V, VI, VII, VIII, X]	76.521 ± 1.302 [I, II, III, IV, V, VI, VII, VIII, X]
	**RT**	0	109.458 ± 2.073	106.204 ± 0.543
		2	47.336 ± 1.117 [I, II, III, IV, V, VI, VII, VIII, IX]	38.841 ± 1.390 [I, II, III, IV, V, VI, VII, VIII, IX]
		4	46.145 ± 0.952 [I, II, III, IV, V, VI, VII, VIII, IX]	31.379 ± 1.196 [I, II, III, IV, V, VI, VII, VIII, IX]
		8	43.739 ± 1.208 [I, II, III, IV, V, VI, VII, VIII, IX]	50.203 ± 1.903 [I, II, III, IV, V, VI, VII, VIII, IX]
		16	40.107 ± 0.729 [I, II, III, IV, V, VI, VII, VIII, IX]	46.482 ± 1.810 [I, II, III, IV, V, VI, VII, VIII, IX]
	**MN**	0	110.487 ± 2.576	104.070 ± 2.143
		2	52.161 ± 0.935 [I, II, III, IV, V, VI, VII, VIII, IX, X]	43.977 ± 1.518 [I, II, III, IV, V, VI, VII, VIII, IX, X]
		4	50.309 ± 1.671 [I, II, III, IV, V, VI, VII, VIII, IX, X]	34.528 ± 1.992 [I, II, III, IV, V, VI, VII, VIII, IX, X]
		8	48.454 ± 2.077 [I, II, III, IV, V, VI, VII, VIII, IX, X]	54.740 ± 1.075 [I, II, III, IV, V, VI, VII, VIII, IX, X]
		16	44.212 ± 1.235 [I, II, III, IV, V, VI, VII, VIII, IX, X]	51.624 ± 2.185 [I, II, III, IV, V, VI, VII, VIII, IX, X]
**Ca1424**	**AM**	0	110.537 ± 2.312	104.273 ± 0.981
		2	98.304 ± 1.464 [IX, X]	93.550 ± 1.002 [IX, X]
		4	96.607 ± 1.665 [IX, X]	88.940 ± 0.514 [IX, X]
		8	92.792 ± 1.125 [IX, X]	81.487 ± 1.609 [IX, X]
		16	88.905 ± 1.195 [IX, X]	99.476 ± 0.816 [IX, X]
	**FL**	0	109.122 ± 2.137	103.834 ± 1.726
		2	97.568 ± 0.402 [IX, X]	93.840 ± 1.333 [IX, X]

	4	96.866 ± 0.750 IX, X	89.269 ± 0.713 IX, X
	8	94.952 ± 0.697 IX, X	83.018 ± 0.609 IX, X
	16	91.735 ± 1.456 IX, X	98.947 ± 0.983 IX, X
**L**	0	106.610 ± 2.592	104.902 ± 0.856
	2	98.631 ± 0.940 IX, X	94.044 ± 0.890 IX, X
	4	96.766 ± 0.718 IX, X	89.417 ± 1.027 IX, X
	8	94.517 ± 0.753 IX, X	82.181 ± 1.321 IX, X
	16	92.108 ± 0.991 IX, X	99.386 ± 0.752 IX, X
**B**	0	110.055 ± 1.943	104.997 ± 1.530
	2	98.242 ± 1.220 IX, X	93.955 ± 1.249 IX, X
	4	97.198 ± 1.489 IX, X	89.362 ± 0.785 IX, X
	8	95.035 ± 1.405 IX, X	82.098 ± 1.413 IX, X
	16	91.434 ± 1.278 IX, X	99.346 ± 0.864 IX, X
**P**	0	109.153 ± 2.024	104.851 ± 1.679
	2	98.253 ± 1.091 IX, X	94.372 ± 1.526 IX, X
	4	97.381 ± 0.822 IX, X	89.141 ± 0.868 IX, X
	8	95.465 ± 1.093 IX, X	80.889 ± 0.598 IX, X
	16	92.089 ± 1.411 IX, X	99.657 ± 0.841 IX, X
**T**	0	110.565 ± 3.190	104.898 ± 1.636
	2	97.722 ± 1.152 IX, X	93.356 ± 1.580 IX, X
	4	97.812 ± 1.063 IX, X	90.059 ± 1.057 IX, X
	8	94.677 ± 0.781 IX, X	81.907 ± 1.541 IX, X
	16	91.152 ± 1.190 IX, X	99.549 ± 0.675 IX, X
**RL**	0	108.525 ± 1.935	105.552 ± 1.288
	2	99.007 ± 1.339 IX, X	94.677 ± 1.300 IX, X
	4	96.667 ± 1.204 IX, X	90.100 ± 1.529 IX, X
	8	94.334 ± 1.102 IX, X	81.639 ± 1.618 IX, X
	16	91.774 ± 1.280 IX, X	99.154 ± 1.095
**RB**	0	110.213 ± 2.785 III	99.687 ± 1.174 IX, X
	2	98.129 ± 1.287 IX, X	94.407 ± 1.228 IX, X
	4	96.233 ± 1.361 IX, X	89.697 ± 0.880 IX, X
	8	92.616 ± 1.006 IX, X	81.696 ± 1.044 IX, X
	16	89.956 ± 1.367 IX, X	99.487 ± 0.878 IX, X
**RP**	0	111.032 ± 2.296 III	103.615 ± 1.715
	2	78.177 ± 0.997 I, II, III, IV, V, VI, VII, VIII, X	73.585 ± 1.158 I, II, III, IV, V, VI, VII, VIII, X
	4	75.780 ± 1.105 I, II, III, IV, V, VI, VII, VIII, X	68.728 ± 0.704 I, II, III, IV, V, VI, VII, VIII, X
	8	73.828 ± 0.895 I, II, III, IV, V, VI, VII, VIII, X	61.353 ± 1.027 I, II, III, IV, V, VI, VII, VIII, X
	16	70.565 ± 0.987 I, II, III, IV, V, VI, VII, VIII, X	69.399 ± 0.937 I, II, III, IV, V, VI, VII, VIII, X
**RT**	0	109.502 ± 1.745	100.680 ± 0.824

		2	48.236 ± 0.943 [I, II, III, IV, V, VI, VII, VIII, IX]	44.505 ± 1.360 [I, II, III, IV, V, VI, VII, VIII, IX]
		4	46.628 ± 1.134 [I, II, III, IV, V, VI, VII, VIII, IX]	39.466 ± 1.190 [I, II, III, IV, V, VI, VII, VIII, IX]
		8	43.007 ± 1.326 [I, II, III, IV, V, VI, VII, VIII, IX]	32.012 ± 1.657 [I, II, III, IV, V, VI, VII, VIII, IX]
		16	39.369 ± 1.284 [I, II, III, IV, V, VI, VII, VIII, IX]	48.970 ± 1.475 [I, II, III, IV, V, VI, VII, VIII, IX]
	**MN**	0	101.429 ± 2.775	102.325 ± 1.288
		2	53.774 ± 1.147 [I, II, III, IV, V, VI, VII, VIII, IX, X]	48.890 ± 1.323 [I, II, III, IV, V, VI, VII, VIII, IX, X]
		4	50.425 ± 1.297 [I, II, III, IV, V, VI, VII, VIII, IX, X]	43.616 ± 2.596 [I, II, III, IV, V, VI, VII, VIII, IX, X]
		8	47.576 ± 1.546 [I, II, III, IV, V, VI, VII, VIII, IX, X]	36.592 ± 1.950 [I, II, III, IV, V, VI, VII, VIII, IX, X]
		16	42.941 ± 1.785 [I, II, III, IV, V, VI, VII, VIII, IX, X]	54.093 ± 2.236 [I, II, III, IV, V, VI, VII, VIII, IX, X]
**Ct1368**	**AM**	0	101.081 ± 2.673	99.136 ± 1.246
		2	97.956 ± 1.661 [IX, X]	97.128 ± 1.572 [IX, X]
		4	95.622 ± 0.970 [IX, X]	93.330 ± 1.451 [IX, X]
		8	92.992 ± 0.651 [IX, X]	89.372 ± 1.744 [IX, X]
		16	89.345 ± 1.141 [IX, X]	80.842 ± 1.227 [IX, X]
	**FL**	0	110.387 ± 2.778	89.943 ± 1.041
		2	97.494 ± 1.263 [IX, X]	97.134 ± 1.501 [IX, X]
		4	95.851 ± 1.343 [IX, X]	94.899 ± 1.785 [IX, X]
		8	93.460 ± 0.880 [IX, X]	89.538 ± 0.843 [IX, X]
		16	90.419 ± 0.479 [IX, X]	81.391 ± 1.033 [IX, X]
	**L**	0	111.035 ± 3.072	99.560 ± 0.906
		2	97.548 ± 0.739 [IX, X]	96.651 ± 1.104 [IX, X]
		4	96.080 ± 1.184 [IX, X]	94.144 ± 1.281 [IX, X]
		8	93.493 ± 1.021 [IX, X]	89.562 ± 1.420 [IX, X]
		16	90.379 ± 0.961 [IX, X]	81.795 ± 0.978 [IX, X]
	**B**	0	108.387 ± 2.287	99.813 ± 1.117
		2	98.203 ± 1.525 [IX, X]	97.296 ± 1.124 [IX, X]
		4	95.820 ± 1.345 [IX, X]	93.640 ± 0.718 [IX, X]
		8	93.417 ± 0.697 [IX, X]	88.726 ± 1.225 [IX, X]
		16	90.225 ± 1.402 [IX, X]	81.086 ± 1.330 [IX, X]
	**P**	0	110.946 ± 0.779	91.468 ± 1.925 [IX, X]
		2	97.686 ± 1.407 [IX, X]	97.893 ± 1.503 [IX, X]
		4	96.182 ± 1.084 [IX, X]	93.049 ± 0.700 [IX, X]
		8	93.555 ± 1.033 [IX, X]	89.555 ± 1.208 [IX, X]
		16	88.846 ± 0.813 [IX, X]	81.568 ± 1.608 [IX, X]
	**T**	0	110.950 ± 1.244	100.789 ± 1.446
		2	97.448 ± 0.883 [IX, X]	97.044 ± 1.222 [IX, X]
		4	95.704 ± 1.237 [IX, X]	93.918 ± 1.678 [IX, X]

		8	93.870 ± 1.225 IX, X	87.819 ± 0.793 IX, X
		16	89.859 ± 1.156 IX, X	80.975 ± 1.608 IX, X
	**RL**	0	110.249 ± 2.778	100.996 ± 1.471
		2	97.368 ± 1.038 IX, X	96.975 ± 1.122 IX, X
		4	96.427 ± 1.194 IX, X	94.155 ± 0.851 IX, X
		8	93.829 ± 1.180 IX, X	89.379 ± 1.218 IX, X
		16	89.591 ± 1.385 IX, X	82.436 ± 0.910 IX, X
	**RB**	0	108.905 ± 2.154	101.076 ± 1.525
		2	97.842 ± 0.885 IX, X	97.365 ± 1.583 IX, X
		4	96.178 ± 1.274 IX, X	92.915 ± 0.776 IX, X
		8	93.383 ± 0.968 IX, X	90.255 ± 1.388 IX, X
		16	89.619 ± 1.238 IX, X	80.981 ± 1.177 IX, X
	**RP**	0	109.704 ± 1.956	109.368 ± 1.772
		2	77.973 ± 1.387 I, II, III, IV, V, VI, VII, VIII, X	67.340 ± 1.079 I, II, III, IV, V, VI, VII, VIII, X
		4	75.523 ± 1.011 I, II, III, IV, V, VI, VII, VIII, X	63.354 ± 1.510 I, II, III, IV, V, VI, VII, VIII, X
		8	72.602 ± 0.949 I, II, III, IV, V, VI, VII, VIII, X	59.540 ± 1.269 I, II, III, IV, V, VI, VII, VIII, X
		16	69.461 ± 0.604 I, II, III, IV, V, VI, VII, VIII, X	52.136 ± 1.533 I, II, III, IV, V, VI, VII, VIII, X
	**RT**	0	110.642 ± 2.372	100.062 ± 1.154
		2	47.535 ± 1.410 I, II, III, IV, V, VI, VII, VIII, IX	47.020 ± 1.179 I, II, III, IV, V, VI, VII, VIII, IX
		4	46.666 ± 1.246 I, II, III, IV, V, VI, VII, VIII, IX	44.811 ± 1.067 I, II, III, IV, V, VI, VII, VIII, IX
		8	43.312 ± 1.344 I, II, III, IV, V, VI, VII, VIII, IX	40.111 ± 1.098 I, II, III, IV, V, VI, VII, VIII, IX
		16	39.731 ± 1.329 I, II, III, IV, V, VI, VII, VIII, IX	32.677 ± 0.649 I, II, III, IV, V, VI, VII, VIII, IX
	**MN**	0	109.037 ± 1.761	105.627 ± 1.439
		2	52.548 ± 1.681 I, II, III, IV, V, VI, VII, VIII, IX, X	52.081 ± 2.063 I, II, III, IV, V, VI, VII, VIII, IX, X
		4	51.251 ± 0.934 I, II, III, IV, V, VI, VII, VIII, IX, X	49.956 ± 1.813 I, II, III, IV, V, VI, VII, VIII, IX, X
		8	47.558 ± 2.505 I, II, III, IV, V, VI, VII, VIII, IX, X	44.201 ± 1.730 I, II, III, IV, V, VI, VII, VIII, IX, X
		16	44.137 ± 1.925 I, II, III, IV, V, VI, VII, VIII, IX, X	37.647 ± 1.040 I, II, III, IV, V, VI, VII, VIII, IX, X
**Ct1432**	**AM**	0	110.502 ± 2.468	100.479 ± 1.391
		2	97.808 ± 1.304 IX, X	99.347 ± 0.656 IX, X
		4	95.920 ± 1.110 IX, X	97.403 ± 0.883 IX, X
		8	93.519 ± 1.439 IX, X	93.661 ± 1.516 IX, X
		16	89.061 ± 1.102 IX, X	89.594 ± 1.737 IX, X
	**FL**	0	109.910 ± 2.998	101.312 ± 1.669
		2	97.934 ± 1.407 IX, X	99.596 ± 0.606 IX, X

	4	96.459 ± 1.235 [IX, X]	96.723 ± 1.832 [IX, X]
	8	93.510 ± 0.587 [IX, X]	93.943 ± 1.797 [IX, X]
	16	89.288 ± 0.998 [IX, X]	88.754 ± 1.305 [IX, X]
**L**	0	109.122 ± 1.585	103.375 ± 1.575
	2	97.884 ± 1.241 [IX, X]	98.823 ± 1.207 [IX, X]
	4	95.423 ± 1.131 [IX, X]	97.172 ± 0.952 [IX, X]
	8	93.772 ± 1.283 [IX, X]	94.141 ± 1.164 [IX, X]
	16	89.879 ± 0.803 [IX, X]	89.437 ± 1.394 [IX, X]
**B**	0	109.637 ± 2.897	102.098 ± 2.060
	2	98.261 ± 1.138 [IX, X]	99.726 ± 0.671 [IX, X]
	4	95.868 ± 0.691 [IX, X]	97.138 ± 1.154 [IX, X]
	8	92.792 ± 1.112 [IX, X]	94.652 ± 1.494 [IX, X]
	16	89.187 ± 1.036 [IX, X]	88.957 ± 1.743 [IX, X]
**P**	0	110.052 ± 3.359	104.636 ± 0.836
	2	98.738 ± 1.119 [IX, X]	99.958 ± 0.103 [IX, X]
	4	95.216 ± 0.870 [IX, X]	96.483 ± 1.196 [IX, X]
	8	93.672 ± 1.391 [IX, X]	94.362 ± 1.543 [IX, X]
	16	89.799 ± 1.383 [IX, X]	89.513 ± 1.672 [IX, X]
**T**	0	110.845 ± 2.410	103.131 ± 1.648
	2	98.130 ± 1.341 [IX, X]	99.042 ± 0.941 [IX, X]
	4	96.100 ± 1.545 [IX, X]	97.594 ± 1.600 [IX, X]
	8	92.327 ± 0.456 [IX, X]	93.184 ± 1.262 [IX, X]
	16	89.064 ± 1.156 [IX, X]	88.467 ± 1.040 [IX, X]
**RL**	0	110.339 ± 2.292	103.292 ± 0.846
	2	98.076 ± 1.032 [IX, X]	99.576 ± 0.749 [IX, X]
	4	95.910 ± 1.449 [IX, X]	96.942 ± 1.676 [IX, X]
	8	93.238 ± 1.124 [IX, X]	94.416 ± 1.137 [IX, X]
	16	90.157 ± 0.625 [IX, X]	89.504 ± 1.524 [IX, X]
**RB**	0	110.371 ± 3.009	104.137 ± 1.502
	2	98.402 ± 1.093 [IX, X]	99.457 ± 1.113 [IX, X]
	4	95.539 ± 0.784 [IX, X]	96.286 ± 1.226 [IX, X]
	8	94.005 ± 1.538 [IX, X]	95.163 ± 1.437 [IX, X]
	16	89.004 ± 0.751 [IX, X]	89.021 ± 1.559 [IX, X]
**RP**	0	111.009 ± 2.190	102.041 ± 0.817
	2	68.509 ± 0.706 [I, II, III, IV, V, VI, VII, VIII, X]	69.665 ± 0.845 [I, II, III, IV, V, VI, VII, VIII, X]
	4	65.345 ± 1.482 [I, II, III, IV, V, VI, VII, VIII, X]	66.474 ± 1.160 [I, II, III, IV, V, VI, VII, VIII, X]
	8	63.099 ± 1.260 [I, II, III, IV, V, VI, VII, VIII, X]	64.657 ± 1.001 [I, II, III, IV, V, VI, VII, VIII, X]
	16	60.086 ± 1.466 [I, II, III, IV, V, VI, VII, VIII, X]	59.530 ± 1.653 [I, II, III, IV, V, VI, VII, VIII, X]
**RT**	0	111.878 ± 1.772	101.690 ± 0.980

		2	47.806 ± 1.055 $^{I, II, III, IV, V, VI, VII, VIII, IX}$	49.757 ± 1.263 $^{I, II, III, IV, V, VI, VII, VIII, IX}$
		4	47.063 ± 0.732 $^{I, II, III, IV, V, VI, VII, VIII, IX}$	46.514 ± 1.481 $^{I, II, III, IV, V, VI, VII, VIII, IX}$
		8	43.885 ± 0.761 $^{I, II, III, IV, V, VI, VII, VIII, IX}$	44.376 ± 1.375 $^{I, II, III, IV, V, VI, VII, VIII, IX}$
		16	40.659 ± 0.416 $^{I, II, III, IV, V, VI, VII, VIII, IX}$	38.471 ± 0.524 $^{I, II, III, IV, V, VI, VII, VIII, IX}$
	**MN**	0	110.796 ± 1.744	108.626 ± 1.576
		2	52.774 ± 1.789 $^{I, II, III, IV, V, VI, VII, VIII, IX, X}$	54.216 ± 1.091 $^{I, II, III, IV, V, VI, VII, VIII, IX, X}$
		4	51.831 ± 1.546 $^{I, II, III, IV, V, VI, VII, VIII, IX, X}$	50.797 ± 1.300 $^{I, II, III, IV, V, VI, VII, VIII, IX, X}$
		8	48.397 ± 1.370 $^{I, II, III, IV, V, VI, VII, VIII, IX, X}$	49.104 ± 1.372 $^{I, II, III, IV, V, VI, VII, VIII, IX, X}$
		16	45.188 ± 1.176 $^{I, II, III, IV, V, VI, VII, VIII, IX, X}$	41.952 ± 0.989 $^{I, II, III, IV, V, VI, VII, VIII, IX, X}$
**Ck779**	**AM**	0	111.494 ± 1.740	99.022 ± 1.083
		2	97.857 ± 0.661 $^{IX, X}$	81.566 ± 1.484 $^{IX, X}$
		4	95.734 ± 1.169 $^{IX, X}$	98.971 ± 1.052 $^{VIII, IX, X}$
		8	93.456 ± 1.148 $^{IX, X}$	96.532 ± 1.351 $^{VIII, IX, X}$
		16	89.537 ± 1.272 $^{IX, X}$	93.942 ± 1.316 $^{VIII, IX, X}$
	**FL**	0	108.272 ± 1.746	99.384 ± 0.976
		2	97.955 ± 1.252 $^{IX, X}$	81.741 ± 0.856 $^{IX, X}$
		4	96.011 ± 1.525 $^{IX, X}$	99.372 ± 1.032 $^{VIII, IX, X}$
		8	92.732 ± 1.271 $^{IX, X}$	96.711 ± 1.156 $^{VIII, IX, X}$
		16	90.101 ± 1.024 $^{IX, X}$	94.476 ± 1.588 $^{VIII, IX, X}$
	**L**	0	109.023 ± 2.087	99.684 ± 0.515
		2	98.039 ± 1.382 $^{IX, X}$	81.454 ± 1.003 $^{IX, X}$
		4	96.459 ± 1.270 $^{IX, X}$	99.803 ± 0.483 $^{VIII, IX, X}$
		8	93.393 ± 1.515 $^{IX, X}$	96.777 ± 1.194 $^{VIII, IX, X}$
		16	89.759 ± 0.998 $^{IX, X}$	93.321 ± 1.445 $^{VIII, IX, X}$
	**B**	0	109.302 ± 2.361	99.134 ± 1.132
		2	98.326 ± 1.288 $^{IX, X}$	81.731 ± 0.751 $^{IX, X}$
		4	96.624 ± 1.365 $^{IX, X}$	99.398 ± 0.855 $^{VIII, IX, X}$
		8	93.407 ± 1.245 $^{IX, X}$	96.918 ± 1.082 $^{VIII, IX, X}$
		16	90.242 ± 0.530 $^{IX, X}$	94.661 ± 1.874 $^{VIII, IX, X}$
	**P**	0	107.596 ± 1.918 $^{I, VIII}$	99.674 ± 0.686
		2	97.510 ± 1.104 $^{IX, X}$	81.207 ± 0.829 $^{IX, X}$
		4	96.397 ± 1.218 $^{IX, X}$	99.403 ± 0.811 $^{VIII, IX, X}$
		8	93.548 ± 1.572 $^{IX, X}$	96.406 ± 1.064 $^{VIII, IX, X}$
		16	89.313 ± 0.991 $^{IX, X}$	99.358 ± 1.201 $^{VIII, IX, X}$
	**T**	0	109.711 ± 3.102	99.691 ± 0.489
		2	98.613 ± 1.561 $^{IX, X}$	81.178 ± 1.596 $^{IX, X}$
		4	95.926 ± 1.098 $^{IX, X}$	99.111 ± 0.720 $^{VIII, IX, X}$

		8	92.523 ± 0.728 [IX, X]	96.437 ± 1.013 [VIII, IX, X]
		16	89.435 ± 1.520 [IX, X]	94.525 ± 1.189 [VIII, IX, X]
	**RL**	0	109.218 ± 3.174	99.631 ± 0.581
		2	97.403 ± 1.460 [IX, X]	82.400 ± 1.033 [IX, X]
		4	96.227 ± 1.267 [IX, X]	99.786 ± 0.342 [VIII, IX, X]
		8	93.382 ± 1.162 [IX, X]	96.353 ± 1.312 [VIII, IX, X]
		16	90.268 ± 0.713 [IX, X]	94.683 ± 1.265 [VIII, IX, X]
	**RB**	0	111.180 ± 3.589 [V]	99.164 ± 0.700
		2	97.923 ± 1.228 [IX, X]	82.397 ± 0.951 [IX, X]
		4	96.926 ± 1.327 [IX, X]	70.435 ± 1.043 [I, II, III, IV, V, VI, VII, X]
		8	93.431 ± 1.206 [IX, X]	67.471 ± 1.372 [I, II, III, IV, V, VI, VII, X]
		16	90.310 ± 0.688 [IX, X]	63.597 ± 1.601 [I, II, III, IV, V, VI, VII, X]
	**RP**	0	108.227 ± 1.164	100.060 ± 1.677
		2	67.241 ± 1.084 [I, II, III, IV, V, VI, VII, VIII, X]	52.027 ± 1.627 [I, II, III, IV, V, VI, VII, VIII, X]
		4	66.633 ± 0.839 [I, II, III, IV, V, VI, VII, VIII, X]	70.586 ± 0.764 [I, II, III, IV, V, VI, VII, X]
		8	63.451 ± 1.598 [I, II, III, IV, V, VI, VII, VIII, X]	66.794 ± 1.366 [I, II, III, IV, V, VI, VII, X]
		16	59.852 ± 1.078 [I, II, III, IV, V, VI, VII, VIII, X]	63.236 ± 0.828 [I, II, III, IV, V, VI, VII, X]
	**RT**	0	109.460 ± 2.788	100.359 ± 1.913
		2	47.556 ± 1.363 [I, II, III, IV, V, VI, VII, VIII, IX]	30.096 ± 0.793 [I, II, III, IV, V, VI, VII, VIII, IX]
		4	46.383 ± 0.996 [I, II, III, IV, V, VI, VII, VIII, IX]	49.582 ± 0.953 [I, II, III, IV, V, VI, VII, VIII, IX]
		8	42.271 ± 0.642 [I, II, III, IV, V, VI, VII, VIII, IX]	45.615 ± 1.405 [I, II, III, IV, V, VI, VII, VIII, IX]
		16	38.359 ± 0.542 [I, II, III, IV, V, VI, VII, VIII, IX]	43.532 ± 1.735 [I, II, III, IV, V, VI, VII, VIII, IX]
	**MN**	0	109.893 ± 1.900	104.391 ± 2.535
		2	51.937 ± 1.826 [I, II, III, IV, V, VI, VII, VIII, IX, X]	54.487 ± 1.133 [I, II, III, IV, V, VI, VII, VIII, IX, X]
		4	50.947 ± 1.194 [I, II, III, IV, V, VI, VII, VIII, IX, X]	55.572 ± 0.969 [I, II, III, IV, V, VI, VII, VIII, IX, X]
		8	45.587 ± 0.802 [I, II, III, IV, V, VI, VII, VIII, IX, X]	50.020 ± 2.242 [I, II, III, IV, V, VI, VII, VIII, IX, X]
		16	42.220 ± 1.242 [I, II, III, IV, V, VI, VII, VIII, IX, X]	47.484 ± 1.454 [I, II, III, IV, V, VI, VII, VIII, IX, X]
**Ck1447**	**AM**	0	108.956 ± 2.536 [VI]	106.375 ± 1.191
		2	97.934 ± 1.218 [VIII, IX, X]	89.317 ± 2.053 [VIII, IX, X]
		4	95.822 ± 0.950 [VIII, IX, X]	82.098 ± 1.327 [VIII, IX, X]
		8	93.481 ± 1.380 [VIII, IX, X]	99.851 ± 0.365 [IV, VIII, IX, X]
		16	89.627 ± 0.973 [VIII, IX, X]	96.190 ± 1.657 [IV, VIII, IX, X]

**FL**	0	109.917 ± 1.413	98.343 ± 1.240
	2	97.835 ± 0.996 [VIII, IX, X]	90.242 ± 1.242 [VIII, IX, X]
	4	96.229 ± 1.356 [VIII, IX, X]	82.235 ± 1.403 [VIII, IX, X]
	8	94.011 ± 1.221 [VIII, IX, X]	99.282 ± 0.807 [IV, VIII, IX, X]
	16	89.922 ± 1.369 [VIII, IX, X]	97.162 ± 1.128 [IV, VIII, IX, X]
**L**	0	108.440 ± 1.557 [VI]	98.045 ± 1.433
	2	97.853 ± 0.708 [VIII, IX, X]	88.421 ± 0.759 [VIII, IX, X]
	4	95.539 ± 1.308 [VIII, IX, X]	80.906 ± 1.963 [VIII, IX, X]
	8	92.500 ± 0.798 [VIII, IX, X]	99.863 ± 0.213 [IV, VIII, IX, X]
	16	88.943 ± 1.679 [VIII, IX, X]	96.303 ± 1.600 [IV, VIII, IX, X]
**B**	0	109.270 ± 3.611 [VI]	98.380 ± 0.593
	2	98.125 ± 1.039 [VIII, IX, X]	88.269 ± 0.961 [VIII, IX, X]
	4	96.540 ± 1.197 [VIII, IX, X]	81.030 ± 1.256 [VIII, IX, X]
	8	92.408 ± 0.903 [VIII, IX, X]	90.343 ± 0.618 [I, II, III, V, VI, VII, VIII, IX, X]
	16	88.769 ± 1.176 [VIII, IX, X]	86.745 ± 1.368 [I, II, III, V, VI, VII, VIII, IX, X]
**P**	0	109.836 ± 2.344	97.230 ± 1.386
	2	97.564 ± 1.231 [VIII, IX, X]	89.375 ± 1.478 [VIII, IX, X]
	4	96.542 ± 1.394 [VIII, IX, X]	81.449 ± 1.593 [VIII, IX, X]
	8	93.378 ± 1.148 [VIII, IX, X]	99.794 ± 0.291 [IV, VIII, IX, X]
	16	89.406 ± 1.259 [VIII, IX, X]	96.835 ± 0.972 [IV, VIII, IX, X]
**T**	0	112.753 ± 1.508 [I, III, IV, VIII, X]	107.338 ± 1.099
	2	97.898 ± 1.319 [VIII, IX, X]	88.445 ± 1.299 [VIII, IX, X]
	4	96.756 ± 1.005 [VIII, IX, X]	81.275 ± 1.280 [VIII, IX, X]
	8	92.804 ± 1.185 [VIII, IX, X]	98.605 ± 1.109 [IV, VIII, IX, X]
	16	89.214 ± 1.006 [VIII, IX, X]	97.324 ± 1.639 [IV, VIII, IX, X]
**RL**	0	110.153 ± 2.034	107.815 ± 1.311
	2	97.313 ± 0.939 [VIII, IX, X]	89.595 ± 2.084 [VIII, IX, X]
	4	96.442 ± 1.511 [VIII, IX, X]	81.389 ± 1.063 [VIII, IX, X]
	8	93.826 ± 1.563 [VIII, IX, X]	99.143 ± 0.758 [IV, VIII, IX, X]
	16	89.227 ± 0.996 [VIII, IX, X]	96.151 ± 1.214 [IV, VIII, IX, X]
**RB**	0	109.271 ± 2.147 [VI]	101.158 ± 0.868
	2	68.339 ± 1.195 [I, II, III, IV, V, VI, VII, X]	58.595 ± 0.654 [I, II, III, IV, V, VI, VII, X]
	4	65.380 ± 1.443 [I, II, III, IV, V, VI, VII, X]	51.374 ± 1.710 [I, II, III, IV, V, VI, VII, X]
	8	62.580 ± 0.745 [I, II, III, IV, V, VI, VII, X]	69.210 ± 1.006 [I, II, III, IV, V, VI, VII, X]
	16	59.230 ± 1.364 [I, II, III, IV, V, VI, VII, X]	66.285 ± 1.558 [I, II, III, IV, V, VI, VII, X]
**RP**	0	110.078 ± 1.529	107.061 ± 0.981
	2	68.013 ± 1.132 [I, II, III, IV, V, VI, VII, X]	59.612 ± 1.518 [I, II, III, IV, V, VI, VII, X]

		4	65.392 ± 0.933 $^{I, II, III, IV, V, VI, VII, X}$	51.949 ± 1.434 $^{I, II, III, IV, V, VI, VII, X}$
		8	63.710 ± 0.905 $^{I, II, III, IV, V, VI, VII, X}$	69.894 ± 0.974 $^{I, II, III, IV, V, VI, VII, X}$
		16	59.598 ± 1.374 $^{I, II, III, IV, V, VI, VII, X}$	67.095 ± 0.828 $^{I, II, III, IV, V, VI, VII, X}$
	**RT**	0	109.382 ± 0.802	106.316 ± 1.282
		2	46.778 ± 0.890 $^{I, II, III, IV, V, VI, VII, VIII, IX}$	39.692 ± 1.446 $^{I, II, III, IV, V, VI, VII, VIII, IX}$
		4	45.153 ± 1.401 $^{I, II, III, IV, V, VI, VII, VIII, IX}$	31.451 ± 1.252 $^{I, II, III, IV, V, VI, VII, VIII, IX}$
		8	43.800 ± 1.255 $^{I, II, III, IV, V, VI, VII, VIII, IX}$	49.618 ± 1.588 $^{I, II, III, IV, V, VI, VII, VIII, IX}$
		16	39.840 ± 0.821 $^{I, II, III, IV, V, VI, VII, VIII, IX}$	46.408 ± 1.900 $^{I, II, III, IV, V, VI, VII, VIII, IX}$
	**MN**	0	109.652 ± 1.944	101.117 ± 1.178
		2	50.961 ± 1.945 $^{I, II, III, IV, V, VI, VII, VIII, IX, X}$	43.930 ± 1.994 $^{I, II, III, IV, V, VI, VII, VIII, IX, X}$
		4	49.737 ± 1.379 $^{I, II, III, IV, V, VI, VII, VIII, IX, X}$	35.836 ± 1.824 $^{I, II, III, IV, V, VI, VII, VIII, IX, X}$
		8	48.148 ± 1.819 $^{I, II, III, IV, V, VI, VII, VIII, IX, X}$	54.239 ± 1.588 $^{I, II, III, IV, V, VI, VII, VIII, IX, X}$
		16	43.735 ± 1.494 $^{I, II, III, IV, V, VI, VII, VIII, IX, X}$	50.344 ± 2.754 $^{I, II, III, IV, V, VI, VII, VIII, IX, X}$
**Cd1470**	**AM**	0	110.507 ± 2.817	104.260 ± 1.000
		2	97.392 ± 0.948 $^{IV, VIII, IX, X}$	95.570 ± 1.152 $^{IV, VIII, IX, X}$
		4	97.370 ± 1.085 $^{IV, VIII, IX, X}$	89.803 ± 1.048 $^{IV, VIII, IX, X}$
		8	93.769 ± 1.089 $^{IV, VIII, IX, X}$	81.532 ± 0.569 $^{IV, VIII, IX, X}$
		16	89.685 ± 0.855 $^{IV, VIII, IX, X}$	99.342 ± 0.746 $^{IV, VIII, IX}$
	**FL**	0	110.791 ± 3.177	105.672 ± 1.219
		2	98.472 ± 0.798 $^{IV, VIII, IX, X}$	94.474 ± 1.521 $^{IV, VIII, IX, X}$
		4	96.592 ± 1.307 $^{IV, VIII, IX, X}$	88.926 ± 1.107 $^{IV, VIII, IX, X}$
		8	93.164 ± 1.293 $^{IV, VIII, IX, X}$	81.426 ± 0.678 $^{IV, VIII, IX, X}$
		16	89.527 ± 0.993 $^{IV, VIII, IX, X}$	99.445 ± 0.587 $^{IV, VIII, IX}$
	**L**	0	110.085 ± 2.099	106.750 ± 1.226
		2	97.250 ± 1.238 $^{IV, VIII, IX, X}$	93.455 ± 1.417 $^{IV, VIII, IX, X}$
		4	95.575 ± 1.011 $^{IV, VIII, IX, X}$	88.514 ± 0.506 $^{IV, VIII, IX, X}$
		8	92.547 ± 0.534 $^{IV, VIII, IX, X}$	81.825 ± 1.248 $^{IV, VIII, IX, X}$
		16	89.828 ± 1.514 $^{IV, VIII, IX, X}$	99.863 ± 0.187 $^{IV, VIII, IX}$
	**B**	0	109.980 ± 1.841	105.657 ± 1.496
		2	87.745 ± 1.417 $^{I, II, III, V, VI, VII, VIII, IX, X}$	85.126 ± 1.440 $^{I, II, III, V, VI, VII, VIII, IX, X}$
		4	86.961 ± 0.918 $^{I, II, III, V, VI, VII, VIII, IX, X}$	79.328 ± 0.748 $^{I, II, III, V, VI, VII, VIII, IX, X}$
		8	83.479 ± 1.082 $^{I, II, III, V, VI, VII, VIII, IX, X}$	71.726 ± 1.391 $^{I, II, III, V, VI, VII, VIII, IX, X}$

	16	79.629 ± 1.442 [I, II, III, V, VI, VII, VIII, IX, X]	89.860 ± 1.114 [I, II, III, V, VI, VII, VIII, IX, X]
**P**	0	109.583 ± 3.437	104.959 ± 1.360
	2	97.815 ± 0.918 [IV, VIII, IX, X]	93.152 ± 0.860 [IV, VIII, IX, X]
	4	95.076 ± 0.665 [IV, VIII, IX, X]	90.532 ± 1.157 [IV, VIII, IX, X]
	8	94.141 ± 0.922 [IV, VIII, IX, X]	80.945 ± 1.413 [IV, VIII, IX, X]
	16	89.128 ± 1.365 [IV, VIII, IX, X]	99.381 ± 0.483 [IV, VIII, IX]
**T**	0	111.315 ± 2.531	105.579 ± 1.157
	2	98.566 ± 1.409 [IV, VIII, IX, X]	93.728 ± 1.276 [IV, VIII, IX, X]
	4	95.935 ± 1.302 [IV, VIII, IX, X]	89.613 ± 0.712 [IV, VIII, IX, X]
	8	93.694 ± 0.891 [IV, VIII, IX, X]	81.948 ± 0.733 [IV, VIII, IX, X]
	16	90.237 ± 0.403 [IV, VIII, IX, X]	99.005 ± 0.874 [IV, VIII, IX]
**RL**	0	110.386 ± 2.476	104.881 ± 1.157
	2	97.405 ± 1.109 [IV, VIII, IX, X]	93.946 ± 1.232 [IV, VIII, IX, X]
	4	95.557 ± 1.022 [IV, VIII, IX, X]	88.725 ± 1.133 [IV, VIII, IX, X]
	8	92.608 ± 1.312 [IV, VIII, IX, X]	81.741 ± 1.018 [IV, VIII, IX, X]
	16	89.723 ± 0.938 [IV, VIII, IX, X]	99.058 ± 1.066 [IV, VIII, IX]
**RB**	0	109.336 ± 2.249	104.830 ± 0.524
	2	67.320 ± 1.585 [I, II, III, IV, V, VI, VII, X]	64.095 ± 1.283 [I, II, III, IV, V, VI, VII, X]
	4	66.147 ± 1.200 [I, II, III, IV, V, VI, VII, X]	59.812 ± 1.547 [I, II, III, IV, V, VI, VII, X]
	8	63.698 ± 1.335 [I, II, III, IV, V, VI, VII, X]	51.744 ± 1.303 [I, II, III, IV, V, VI, VII, X]
	16	59.831 ± 1.191 [I, II, III, IV, V, VI, VII, X]	100.333 ± 1.190 [I, II, III, IV, V, VI, VII, X]
**RP**	0	109.821 ± 3.150	104.721 ± 1.271
	2	67.746 ± 1.066 [I, II, III, IV, V, VI, VII, X]	63.445 ± 1.288 [I, II, III, IV, V, VI, VII, X]
	4	66.014 ± 1.189 [I, II, III, IV, V, VI, VII, X]	59.725 ± 1.136 [I, II, III, IV, V, VI, VII, X]
	8	63.400 ± 1.055 [I, II, III, IV, V, VI, VII, X]	51.590 ± 1.092 [I, II, III, IV, V, VI, VII, X]
	16	59.377 ± 1.278 [I, II, III, IV, V, VI, VII, X]	70.809 ± 1.249 [I, II, III, IV, V, VI, VII, X]
**RT**	0	109.992 ± 1.689	104.197 ± 1.184
	2	47.476 ± 1.243 [I, II, III, IV, V, VI, VII, VIII, IX]	44.411 ± 1.179 [I, II, III, IV, V, VI, VII, VIII, IX]
	4	46.602 ± 1.238 [I, II, III, IV, V, VI, VII, VIII, IX]	39.708 ± 1.199 [I, II, III, IV, V, VI, VII, VIII, IX]
	8	43.189 ± 1.107 [I, II, III, IV, V, VI, VII, VIII, IX]	30.567 ± 1.195 [I, II, III, IV, V, VI, VII, VIII, IX]
	16	38.787 ± 1.131 [I, II, III, IV, V, VI, VII, VIII, IX]	29.514 ± 0.968 [IV, VIII, IX]
**MN**	0	110.449 ± 1.973	108.133 ± 1.668
	2	51.743 ± 2.744 [I, II, III, IV, V, VI, VII, VIII, IX, X]	49.298 ± 1.694 [I, II, III, IV, V, VI, VII, VIII, IX, X]

		4	50.603 ± 1.596 $^{I, II, III, IV, V, VI, VII, VIII, IX, X}$	43.685 ± 1.758 $^{I, II, III, IV, V, VI, VII, VIII, IX, X}$
		8	47.562 ± 1.343 $^{I, II, III, IV, V, VI, VII, VIII, IX, X}$	34.857 ± 1.429 $^{I, II, III, IV, V, VI, VII, VIII, IX, X}$
		16	42.881 ± 1.564 $^{I, II, III, IV, V, VI, VII, VIII, IX, X}$	30.000 ± 0.000 $^{IV, VIII, IX}$
**Cd1471**	**AM**	0	107.879 ± 2.515	109.790 ± 2.144
		2	97.224 ± 1.265 $^{IV, VIII, IX}$	96.545 ± 1.500 $^{IV, VIII, IX}$
		4	96.108 ± 1.045 $^{IV, VIII, IX}$	93.697 ± 1.435 $^{IV, VIII, IX}$
		8	94.052 ± 0.502 $^{IV, VIII, IX}$	89.801 ± 0.353 $^{IV, VIII, IX}$
		16	90.100 ± 1.173 $^{IV, VIII, IX}$	82.056 ± 1.673 $^{IV, VIII, IX}$
	**FL**	0	109.883 ± 1.690	101.382 ± 0.907
		2	97.543 ± 0.855 $^{IV, VIII, IX}$	96.630 ± 1.094 $^{IV, VIII, IX}$
		4	95.940 ± 1.107 $^{IV, VIII, IX}$	93.985 ± 1.255 $^{IV, VIII, IX}$
		8	92.954 ± 0.774 $^{IV, VIII, IX}$	89.243 ± 0.834 $^{IV, VIII, IX}$
		16	89.294 ± 1.049 $^{IV, VIII, IX}$	81.203 ± 1.128 $^{IV, VIII, IX, X}$
	**L**	0	111.456 ± 2.018 $^{I, VII}$	101.570 ± 1.050
		2	98.300 ± 1.444 $^{IV, VIII, IX}$	97.484 ± 1.224 $^{IV, VIII, IX}$
		4	96.488 ± 1.186 $^{IV, VIII, IX}$	94.194 ± 1.482 $^{IV, VIII, IX}$
		8	93.172 ± 1.152 $^{IV, VIII, IX}$	89.062 ± 0.988 $^{IV, VIII, IX}$
		16	89.446 ± 1.448 $^{IV, VIII, IX}$	81.247 ± 1.417 $^{IV, VIII, IX, X}$
	**B**	0	110.140 ± 2.571	101.185 ± 1.508
		2	87.947 ± 0.624 $^{I, II, III, V, VI, VII, VIII, IX, X}$	86.925 ± 1.187 $^{I, II, III, V, VI, VII, VIII, IX, X}$
		4	86.646 ± 0.966 $^{I, II, III, V, VI, VII, VIII, IX, X}$	84.617 ± 0.724 $^{I, II, III, V, VI, VII, VIII, IX, X}$
		8	83.854 ± 0.483 $^{I, II, III, V, VI, VII, VIII, IX, X}$	79.838 ± 0.950 $^{I, II, III, V, VI, VII, VIII, IX, X}$
		16	79.745 ± 1.076 $^{I, II, III, V, VI, VII, VIII, IX, X}$	71.910 ± 1.631 $^{I, II, III, V, VI, VII, VIII, IX, X}$
	**P**	0	109.266 ± 2.861	101.499 ± 1.069
		2	97.211 ± 0.770 $^{IV, VIII, IX}$	95.911 ± 0.800 $^{IV, VIII, IX}$
		4	96.872 ± 1.283 $^{IV, VIII, IX}$	95.408 ± 1.151 $^{IV, VIII, IX}$
		8	92.678 ± 1.077 $^{IV, VIII, IX}$	88.487 ± 1.716 $^{IV, VIII, IX}$
		16	89.748 ± 1.382 $^{IV, VIII, IX}$	81.801 ± 1.545 $^{IV, VIII, IX}$
	**T**	0	108.770 ± 2.126	103.013 ± 1.502
		2	98.124 ± 1.340 $^{IV, VIII, IX}$	97.644 ± 1.488 $^{IV, VIII, IX}$
		4	96.270 ± 1.005 $^{IV, VIII, IX}$	93.814 ± 0.895 $^{IV, VIII, IX}$
		8	93.720 ± 1.494 $^{IV, VIII, IX}$	89.674 ± 1.705 $^{IV, VIII, IX}$
		16	89.906 ± 1.188 $^{IV, VIII, IX}$	82.289 ± 1.215 $^{IV, VIII, IX}$
	**RL**	0	107.597 ± 1.797 $^{III, VIII, IX}$	102.106 ± 1.527
		2	98.556 ± 1.283 $^{IV, VIII, IX}$	97.128 ± 1.080 $^{IV, VIII, IX}$
		4	96.160 ± 0.827 $^{IV, VIII, IX}$	94.270 ± 1.079 $^{IV, VIII, IX}$
		8	92.877 ± 1.129 $^{IV, VIII, IX}$	88.745 ± 1.572 $^{IV, VIII, IX}$
		16	89.200 ± 1.305 $^{IV, VIII, IX}$	81.063 ± 1.289 $^{IV, VIII, IX, X}$

**RB**	0	111.533 ± 3.148 [I, VII]	101.430 ± 1.539
	2	68.279 ± 1.361 [I, II, III, IV, V, VI, VII, X]	67.089 ± 1.929 [I, II, III, IV, V, VI, VII, X]
	4	65.787 ± 1.134 [I, II, III, IV, V, VI, VII, X]	63.815 ± 1.112 [I, II, III, IV, V, VI, VII, X]
	8	62.724 ± 0.994 [I, II, III, IV, V, VI, VII, X]	58.589 ± 1.248 [I, II, III, IV, V, VI, VII, X]
	16	59.165 ± 0.901 [I, II, III, IV, V, VI, VII, X]	51.330 ± 1.271 [I, II, III, IV, V, VI, VII, X]
**RP**	0	110.947 ± 3.027 [VII]	108.856 ± 1.592
	2	67.898 ± 1.596 [I, II, III, IV, V, VI, VII, X]	67.025 ± 1.703 [I, II, III, IV, V, VI, VII, X]
	4	66.462 ± 1.039 [I, II, III, IV, V, VI, VII, X]	64.374 ± 0.837 [I, II, III, IV, V, VI, VII, X]
	8	63.069 ± 1.220 [I, II, III, IV, V, VI, VII, X]	59.029 ± 1.326 [I, II, III, IV, V, VI, VII, X]
	16	58.731 ± 0.824 [I, II, III, IV, V, VI, VII, X]	50.856 ± 0.677 [I, II, III, IV, V, VI, VII, X]
**RT**	0	110.504 ± 2.228	109.691 ± 0.956
	2	98.872 ± 0.728 [IV, VIII, IX]	97.574 ± 0.541 [IV, VIII, IX]
	4	97.161 ± 1.147 [IV, VIII, IX]	95.011 ± 1.331 [IV, VIII, IX]
	8	93.718 ± 0.451 [IV, VIII, IX]	90.091 ± 0.957 [IV, VIII, IX]
	16	91.952 ± 1.474 [IV, VIII, IX]	84.130 ± 1.619 [II, III, IV, VII, VIII, IX]
**MN**	0	109.225 ± 3.120	104.349 ± 2.020
	2	78.924 ± 2.581 [I, II, III, IV, V, VI, VII, VIII, IX, X]	66.266 ± 5.026 [I, II, IV, V, VII, VIII, IX]
	4	55.969 ± 3.053 [I, II, III, IV, V, VI, VII, VIII, IX, X]	52.555 ± 3.555 [I, II, III, IV, V, VI, VII, VIII, IX, X]
	8	42.407 ± 2.509 [I, II, III, IV, V, VI, VII, VIII, IX, X]	38.247 ± 1.848 [I, II, III, IV, V, VI, VII, VIII, IX, X]
	16	30.098 ± 1.260 [I, II, III, IV, V, VI, VII, VIII, IX, X]	32.072 ± 1.364 [I, II, III, IV, V, VI, VII, VIII, IX, X]

I: statistically significant ($p < .001$) when compared to AM at respective sampling duration
II: statistically significant ($p < .001$) when compared to FL at respective sampling duration
III: statistically significant ($p < .001$) when compared to L at respective sampling duration
IV: statistically significant ($p < .001$) when compared to B at respective sampling duration
V: statistically significant ($p < .001$) when compared to P at respective sampling duration
VI: statistically significant ($p < .001$) when compared to T at respective sampling duration
VII: statistically significant ($p < .001$) when compared to RL at respective sampling duration
VIII: statistically significant ($p < .001$) when compared to RB at respective sampling duration
IX: statistically significant ($p < .001$) when compared to RP at respective sampling duration
X: statistically significant ($p < .001$) when compared to RT at respective sampling duration

Table S7. Reduced percentage in total biofilm biomass (BB), biofilm attached cellular metabolic activity (BMT) and viability (BVA) compared to untreated biofilm control (UBC). All treatments are significantly different compared to untreated control (UBC, *n*=6, p < .001).

Strain	Treatment	%BB (mean ± SE)	%BMT (mean ± SE)	%BVA (mean ± SE)
Ca1372	AM	30.314 ± 1.171 [II, III, IV, V, VI, VII, VIII, IX, X]	34.453 ± 1.156 [II, III, IV, V, VI, VII, VIII, IX, X]	25.016 ± 0.979 [II, III, IV, V, VI, VII, VIII, IX, X]
	FL	103.309 ± 1.634 [I, III, IV, V, VI, VII, VIII, IX, X]	53.506 ± 1.559 [I, III, IV, V, VI, VII, IX]	48.655 ± 1.386 [I, III, IV, V, VI, VII, IX, X]
	L	113.858 ± 1.846 [I, II, X]	57.961 ± 1.839 [I, II, VIII, X]	56.752 ± 1.136 [I, II, VIII, X]
	B	114.021 ± 1.350 [I, II, X]	57.276 ± 1.103 [I, II, VIII, X]	58.279 ± 1.676 [I, II, VIII, X]
	P	115.142 ± 2.300 [I, II, X]	58.372 ± 1.157 [I, II, VIII, X]	57.004 ± 0.798 [I, II, VIII, X]
	T	114.016 ± 2.198 [I, II, X]	58.396 ± 1.746 [I, II, VIII, X]	58.267 ± 1.690 [I, II, VIII, X]
	RL	113.709 ± 2.394 [I, II, X]	58.114 ± 1.005 [I, II, VIII, X]	56.684 ± 1.343 [I, II, VIII, X]
	RB	113.150 ± 2.372 [I, II, X]	54.276 ± 1.209 [I, III, IV, V, VI, VII, IX, X]	51.113 ± 1.137 [I, III, IV, V, VI, VII, IX, X]
	RP	114.185 ± 1.941 [I, II, X]	58.849 ± 1.390 [I, II, VIII, X]	57.555 ± 1.500 [I, II, VIII, X]
	RT	66.542 ± 1.680 [I, II, III, IV, V, VI, VII, VIII, IX]	50.608 ± 0.824 [I, III, IV, V, VI, VII, VIII, IX]	41.797 ± 1.133 [I, II, III, IV, V, VI, VII, VIII, IX]
Ca1423	AM	32.866 ± 0.718 [II, III, IV, V, VI, VII, VIII, IX, X]	37.327 ± 1.109 [II, III, IV, V, VI, VII, VIII, IX, X]	27.215 ± 0.401 [II, III, IV, V, VI, VII, VIII, IX, X]
	FL	113.730 ± 2.523 [I]	57.229 ± 0.969 [I]	58.158 ± 1.288 [I]
	L	115.334 ± 2.038 [I]	57.901 ± 1.216 [I]	58.066 ± 0.647 [I]
	B	112.841 ± 1.791 [I]	57.427 ± 0.820 [I]	58.472 ± 1.693 [I]
	P	113.723 ± 2.500 [I]	58.294 ± 1.308 [I]	57.964 ± 1.119 [I]
	T	113.858 ± 2.307 [I]	56.929 ± 0.715 [I]	57.350 ± 1.575 [I]
	RL	114.068 ± 2.153 [I]	58.479 ± 1.358 [I]	56.900 ± 1.154 [I]
	RB	112.291 ± 2.171 [I]	57.602 ± 1.149 [I]	58.291 ± 1.172 [I]
	RP	113.907 ± 2.185 [I]	58.172 ± 1.475 [I]	57.431 ± 1.308 [I]
	RT	114.750 ± 1.552 [I]	58.464 ± 1.386 [I]	58.350 ± 1.877 [I]
Ca1424	AM	30.666 ± 0.998 [II, III, IV, V, VI, VII, VIII, IX, X]	34.998 ± 1.070 [II, III, IV, V, VI, VII, VIII, IX, X]	25.849 ± 0.422 [II, III, IV, V, VI, VII, VIII, IX, X]
	FL	114.818 ± 2.246 [I]	58.156 ± 1.573 [I]	58.644 ± 1.706 [I]
	L	113.728 ± 2.915 [I]	57.464 ± 1.436 [I]	57.970 ± 1.179 [I]
	B	113.598 ± 2.673 [I]	58.354 ± 1.826 [I]	59.187 ± 1.042 [I]
	P	113.634 ± 3.024 [I]	58.736 ± 1.319 [I]	57.826 ± 1.435 [I]
	T	112.179 ± 1.478 [I]	57.806 ± 1.824 [I]	56.997 ± 0.992 [I]
	RL	114.063 ± 1.096 [I]	57.773 ± 1.558 [I]	57.876 ± 1.394 [I]
	RB	114.651 ± 2.850 [I]	57.174 ± 1.567 [I]	57.009 ± 1.036 [I]
	RP	114.039 ± 2.692 [I]	58.078 ± 1.538 [I]	58.944 ± 1.442 [I]
	RT	113.704 ± 2.079 [I]	58.089 ± 1.651 [I]	57.723 ± 1.661 [I]
Ct1368	AM	29.039 ± 0.725 [II, III, IV, V, VI, VII, VIII, IX, X]	33.941 ± 0.465 [II, III, IV, V, VI, VII, VIII, IX, X]	24.439 ± 1.045 [II, III, IV, V, VI, VII, VIII, IX, X]
	FL	114.476 ± 1.646 [I]	57.449 ± 1.145 [I]	58.209 ± 1.417 [I]

	**L**	113.593 ± 2.711 [I]	58.116 ± 1.502 [I]	58.390 ± 1.666 [I]
	**B**	114.375 ± 2.547 [I]	58.494 ± 1.043 [I]	57.582 ± 1.219 [I]
	**P**	113.205 ± 2.851 [I]	57.953 ± 1.704 [I]	57.962 ± 1.306 [I]
	**T**	112.436 ± 2.067 [I]	56.846 ± 1.164 [I]	59.016 ± 1.076 [I]
	**RL**	113.171 ± 2.257 [I]	58.059 ± 1.525 [I]	58.699 ± 1.747 [I]
	**RB**	113.409 ± 2.971 [I]	57.701 ± 1.263 [I]	58.285 ± 1.785 [I]
	**RP**	113.721 ± 2.725 [I]	57.141 ± 1.075 [I]	57.111 ± 1.440 [I]
	**RT**	113.137 ± 1.971 [I]	57.912 ± 1.314 [I]	56.552 ± 0.865 [I]
**Ct1432**	**AM**	28.265 ± 0.982 [II, III, IV, V, VI, VII, VIII, IX, X]	33.916 ± 1.092 [II, III, IV, V, VI, VII, VIII, IX, X]	24.495 ± 0.591 [II, III, IV, V, VI, VII, VIII, IX, X]
	**FL**	113.269 ± 2.092 [I]	58.539 ± 1.895 [I]	58.059 ± 1.380 [I]
	**L**	114.348 ± 1.971 [I]	57.489 ± 0.985 [I]	57.891 ± 0.834 [I]
	**B**	114.129 ± 2.485 [I]	58.572 ± 1.432 [I]	58.553 ± 1.529 [I]
	**P**	113.061 ± 2.148 [I]	58.260 ± 1.878 [I]	59.478 ± 1.218 [I]
	**T**	114.403 ± 2.527 [I]	58.203 ± 1.602 [I]	57.252 ± 1.586 [I]
	**RL**	113.659 ± 2.923 [I]	56.791 ± 1.579 [I]	59.127 ± 0.887 [I]
	**RB**	113.226 ± 1.814 [I]	58.328 ± 1.590 [I]	58.371 ± 1.730 [I]
	**RP**	115.258 ± 2.345 [I]	57.195 ± 1.359 [I]	58.373 ± 1.701 [I]
	**RT**	113.380 ± 2.390 [I]	58.037 ± 1.598 [I]	57.248 ± 1.031 [I]
**Ck779**	**AM**	28.644 ± 0.813 [II, III, IV, V, VI, VII, VIII, IX, X]	33.316 ± 0.771 [II, III, IV, V, VI, VII, VIII, IX, X]	23.999 ± 0.675 [II, III, IV, V, VI, VII, VIII, IX, X]
	**FL**	42.830 ± 0.902 [I, III, IV, V, VI, VII, VIII, IX, X]	40.720 ± 0.761 [I, III, IV, V, VI, VII, VIII, IX, X]	31.398 ± 0.741 [I, III, IV, V, VI, VII, VIII, IX, X]
	**L**	112.719 ± 1.698 [I, II]	57.552 ± 1.525 [I, II]	57.791 ± 0.783 [I, II]
	**B**	112.660 ± 2.161 [I, II]	58.231 ± 1.293 [I, II]	56.968 ± 0.762 [I, II]
	**P**	113.117 ± 2.667 [I, II]	58.567 ± 0.773 [I, II]	58.364 ± 1.688 [I, II]
	**T**	115.576 ± 1.749 [I, II]	58.141 ± 1.248 [I, II]	58.494 ± 1.437 [I, II]
	**RL**	112.696 ± 1.967 [I, II]	58.391 ± 1.326 [I, II]	56.778 ± 1.141 [I, II]
	**RB**	114.147 ± 2.080 [I, II]	58.400 ± 1.519 [I, II]	59.080 ± 1.388 [I, II]
	**RP**	113.253 ± 2.587 [I, II]	58.431 ± 1.562 [I, II]	58.100 ± 1.671 [I, II]
	**RT**	114.023 ± 2.638 [I, II]	57.900 ± 1.444 [I, II]	57.379 ± 1.445 [I, II]
**Ck1447**	**AM**	27.813 ± 0.612 [II, III, IV, V, VI, VII, VIII, IX, X]	34.487 ± 1.027 [II, III, IV, V, VI, VII, VIII, IX, X]	24.849 ± 0.290 [II, III, IV, V, VI, VII, VIII, IX, X]
	**FL**	114.350 ± 2.470 [I]	58.402 ± 1.902 [I]	58.098 ± 1.170 [I]
	**L**	112.646 ± 1.221 [I]	57.842 ± 1.576 [I]	57.251 ± 0.695 [I]
	**B**	113.540 ± 2.364 [I]	58.706 ± 1.733 [I]	57.157 ± 0.812 [I]
	**P**	112.216 ± 1.247 [I]	58.738 ± 1.453 [I]	58.096 ± 1.766 [I]
	**T**	113.464 ± 2.947 [I]	57.399 ± 1.611 [I]	58.320 ± 1.462 [I]
	**RL**	113.984 ± 2.859 [I]	58.687 ± 1.555 [I]	58.623 ± 0.781 [I]
	**RB**	112.439 ± 2.606 [I]	58.246 ± 1.030 [I]	58.021 ± 1.321 [I]
	**RP**	114.029 ± 2.661 [I]	57.479 ± 1.596 [I]	58.378 ± 1.338 [I]
	**RT**	113.058 ± 2.759 [I]	58.858 ± 1.381 [I]	57.779 ± 1.379 [I]

**Cd1470**	**AM**	15.769 ± 0.828 [II, III, IV, V, VI, VII, VIII, IX, X]	25.603 ± 0.708 [II, III, IV, V, VI, VII, VIII, IX, X]	16.519 ± 0.362 [II, III, IV, V, VI, VII, VIII, IX, X]
	**FL**	112.550 ± 1.760 [I]	58.277 ± 1.480 [I]	58.592 ± 1.550 [I]
	**L**	113.441 ± 1.737 [I]	58.877 ± 1.156 [I]	57.894 ± 1.608 [I]
	**B**	113.878 ± 1.486 [I]	58.163 ± 1.478 [I]	57.992 ± 1.973 [I]
	**P**	114.951 ± 1.968 [I]	58.035 ± 1.632 [I]	59.069 ± 1.433 [I]
	**T**	114.449 ± 2.839 [I]	58.513 ± 1.590 [I]	59.292 ± 0.726 [I]
	**RL**	112.076 ± 1.138 [I]	58.072 ± 1.589 [I]	58.331 ± 1.315 [I]
	**RB**	114.007 ± 1.658 [I]	58.705 ± 1.100 [I]	58.729 ± 1.530 [I]
	**RP**	113.341 ± 2.105 [I]	57.270 ± 1.811 [I]	58.043 ± 1.622 [I]
	**RT**	114.576 ± 2.128 [I]	58.001 ± 1.149 [I]	58.625 ± 1.118 [I]
**Cd1471**	**AM**	17.248 ± 0.593 [II, III, IV, V, VI, VII, VIII, IX, X]	26.900 ± 0.404 [II, III, IV, V, VI, VII, VIII, IX, X]	17.617 ± 0.434 [II, III, IV, V, VI, VII, VIII, IX, X]
	**FL**	27.988 ± 0.738 [I, III, IV, V, VI, VII, VIII, IX, X]	34.275 ± 0.861 [I, III, IV, V, VI, VII, VIII, IX, X]	24.698 ± 0.816 [I, III, IV, V, VI, VII, VIII, IX, X]
	**L**	113.306 ± 2.299 [I, II]	57.595 ± 2.006 [I, II]	57.022 ± 1.591 [I, II]
	**B**	115.772 ± 1.995 [I, II]	59.229 ± 0.942 [I, II]	58.242 ± 1.292 [I, II]
	**P**	115.290 ± 2.392 [I, II]	58.352 ± 0.886 [I, II]	57.668 ± 1.357 [I, II]
	**T**	114.894 ± 2.168 [I, II]	57.707 ± 1.420 [I, II]	57.506 ± 0.734 [I, II]
	**RL**	114.408 ± 2.314 [I, II]	57.273 ± 1.205 [I, II]	56.880 ± 1.372 [I, II]
	**RB**	113.405 ± 1.211 [I, II]	58.403 ± 1.385 [I, II]	57.553 ± 1.930 [I, II]
	**RP**	113.338 ± 2.232 [I, II]	57.048 ± 0.629 [I, II]	58.701 ± 1.471 [I, II]
	**RT**	113.612 ± 2.423 [I, II]	58.474 ± 1.180 [I, II]	58.980 ± 0.919 [I, II]

I: statistically significant ($p < .001$) when compared to AM
II: statistically significant ($p < .001$) when compared to FL
III: statistically significant ($p < .001$) when compared to L
IV: statistically significant ($p < .001$) when compared to B
V: statistically significant ($p < .001$) when compared to P
VI: statistically significant ($p < .001$) when compared to T
VII: statistically significant ($p < .001$) when compared to RL
VIII: statistically significant ($p < .001$) when compared to RB
IX: statistically significant ($p < .001$) when compared to RP
X: statistically significant ($p < .001$) when compared to RT

Table S8. Pairwise distance matrix from hierarchical clustering of treatment-induced mechanistic signatures (ROS/RNS and antioxidant gene responses)

	AM	FL	L	B	P	T	RL	RB	RP	RT	MN
**AM**	0.00	11.36	18.70	17.91	18.32	16.83	16.77	12.72	17.25	9.03	17.57
**FL**	11.36	0.00	0.94	0.76	0.85	0.54	0.53	0.07	0.63	0.26	35.05
**L**	18.70	0.94	0.00	0.02	0.01	0.06	0.07	0.69	0.04	2.04	44.47
**B**	17.91	0.76	0.02	0.00	0.00	0.02	0.02	0.51	0.01	1.74	43.98
**P**	18.32	0.85	0.01	0.00	0.00	0.04	0.04	0.59	0.02	1.89	44.45
**T**	16.83	0.54	0.06	0.02	0.04	0.00	0.00	0.34	0.01	1.40	42.47
**RL**	16.77	0.53	0.07	0.02	0.04	0.00	0.00	0.34	0.01	1.39	42.34
**RB**	12.72	0.07	0.69	0.51	0.59	0.34	0.34	0.00	0.41	0.38	37.57
**RP**	17.25	0.63	0.04	0.01	0.02	0.01	0.01	0.41	0.00	1.55	42.89
**RT**	9.03	0.26	2.04	1.74	1.89	1.40	1.39	0.38	1.55	0.00	32.91
**MN**	17.57	35.05	44.47	43.98	44.45	42.47	42.34	37.57	42.89	32.91	0.00

Distances were computed from treatment-level mechanistic profiles derived from species-wise Z-standardized endpoints (ROS, RNS, *CAT1*, *GPX1*, *SOD1*), summarized as treatment × species mean signatures (11 treatments across 4 species). Lower distances indicate greater similarity in overall oxidative–nitrosative/antioxidant response patterns; larger distances indicate mechanistically divergent signatures. Controls were included as anchors: AM and FL (antifungal reference controls) and MN (oxidative-stress positive control). Hierarchical clustering used Euclidean distance with group-average linkage.

Table S9. Principal component analysis of mechanistic markers: eigenvalues and variance explained (unrotated and Varimax-rotated solutions).

Component	Initial Eigenvalues			Extraction Sums of Squared Loadings			Rotation Sums of Squared Loadings		
	**Total**	**% of Variance**	**Cumulative %**	**Total**	**% of Variance**	**Cumulative %**	**Total**	**% of Variance**	**Cumulative %**
**1**	4.311	86.228	86.228	4.311	86.228	86.228	3.625	72.510	72.510
**2**	0.654	13.088	99.316	0.654	13.088	99.316	1.340	26.806	99.316
**3**	0.028	0.565	99.881						
**4**	0.004	0.086	99.967						
**5**	0.002	0.033	100.000						

PCA was performed on treatment × species mean signatures of species-wise Z-standardized ROS, RNS, *CAT1*, *GPX1*, and *SOD1* (N = 44 signatures). Initial eigenvalues report variance captured by each component prior to rotation; extraction sums of squared loadings correspond to the retained component solution; and rotation sums of squared loadings indicate variance redistributed after Varimax rotation (orthogonal). Percent variance and cumulative percent variance are reported for interpretation of dominant mechanistic axes.

Table S10. Rotated component matrix (Varimax) from PCA: loading vectors of ROS/RNS and antioxidant genes

Markers	Component	
	**1**	**2**
**ROS**	2.913	-0.663
**RNS**	2.895	-0.666
CAT1	-2.745	1.197
GPX1	2.745	-1.203
SOD1	-0.834	2.88

Values are rotated component loadings from PCA applied to treatment × species mean signatures of species-wise Z-standardized mechanistic endpoints (ROS, RNS, *CAT1*, *GPX1*, *SOD1*). Loadings indicate the direction and magnitude with which each marker contributes to each principal component (higher absolute values denote stronger contributions). Component 1 represents the dominant mechanistic axis in this dataset, while Component 2 captures secondary structure following orthogonal Varimax rotation.

Table S11. Treatment centroids on the PCA mechanistic landscape (PC1-PC2) derived from within-species Z-standardized ROS/RNS and antioxidant gene signatures (*CAT1*, *GPX1*, *SOD1*)

Treatment	PC1 centroid	PC2 centroid
**AM**	0.15	-2.63
**FL**	-0.25	-0.09
**L**	-0.38	0.6
**B**	-0.41	0.48
**P**	-0.41	0.52
**T**	-0.38	0.4
**RL**	-0.38	0.4
**RB**	-0.34	-0.05
**RP**	-0.38	0.45
**RT**	-0.29	-0.52
**MN**	3.08	0.46

PC1 and PC2 values are treatment centroids (means of PCA factor scores) computed from treatment × species mechanistic signatures (ROS, RNS, *CAT1*, *GPX1*, *SOD1*) after within-species Z-standardization and aggregation to the treatment × species level (n = 4 signatures per treatment; one per species). Positive PC1 reflects higher oxidative/nitrosative stress loading (ROS/RNS/*SOD1* direction) with an opposing *GPX1* contribution, whereas PC2 primarily reflects *CAT1*-centered antioxidant response variation. AM, FL, and MN are reference/anchor controls; L, B, P, T are essential oils and RL, RB, RP, RT are their corresponding RAMEB inclusion-complex formulations.

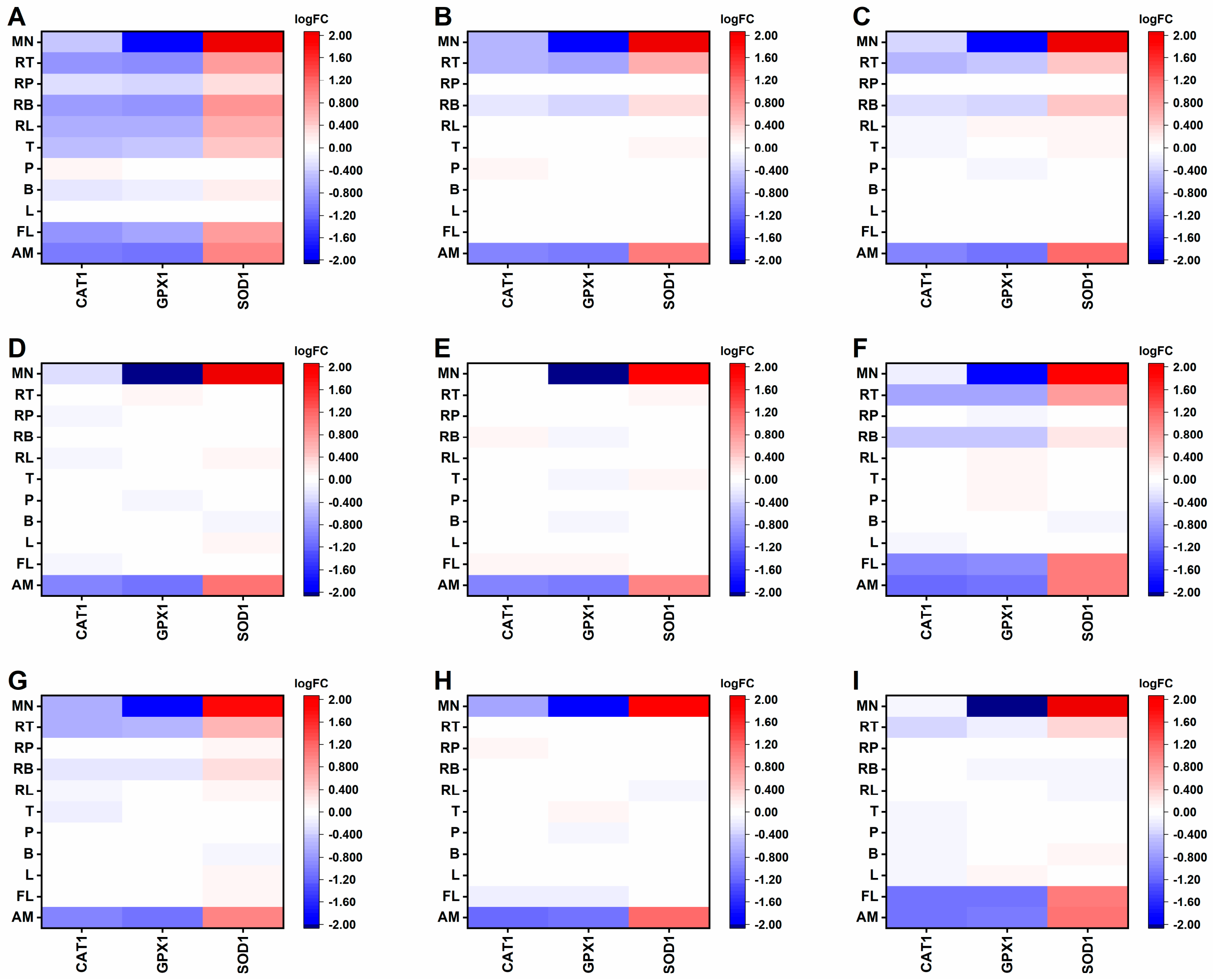

Figure S1. Antioxidant-gene response landscape across treatments (*CAT1*, *GPX1*, *SOD1*).

Heatmaps show treatment-associated changes in antioxidant gene programs for *CAT1*, *GPX1*, and *SOD1* across the full treatment panel (rows; MN, RT, RP, RB, RL, T, P, B, L, FL, AM). Color indicates log fold change (logFC: scale −2 to +2), with red = upregulation and blue = downregulation relative to the reference condition defined in your analysis pipeline. Panels **A-I** correspond to distinct experimental Candida strains (**A**: Ca1372, **B**: Ca1423, **C**: Ca1424, **D**: Ct1368, **E**: Ct1432, **F**: Ck779, **G**: Ck1447, **H**: Cd1470 and **I**: Cd1471); panel labels should be matched to the exact grouping used during aggregation/normalization. Values are derived from aggregated Treatment-level signatures (means) and plotted on a common logFC scale to enable cross-panel comparison; clustering/order is fixed to preserve treatment-to-treatment visual comparability across panels.

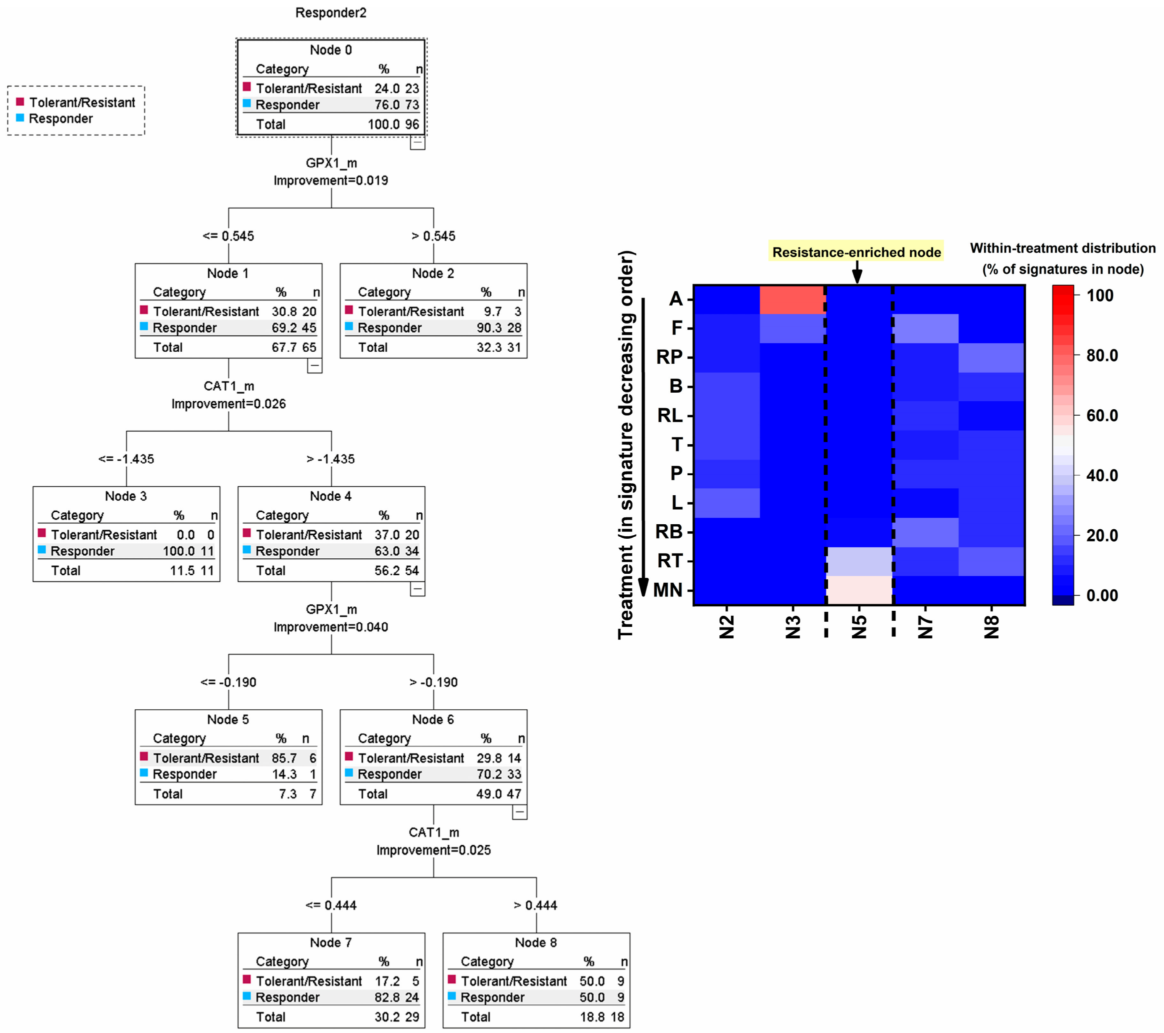

Figure S2. CRT identifies *GPX1-CAT1* thresholds that separate responder vs tolerant/resistant phenotypes and shows treatment enrichment across terminal nodes (controls included).

Left: Classification and regression tree (CRT; dependent variable Responder2, coded 1 = responder (susceptible + hormetic/adaptive) and 0 = tolerant/resistant) fitted to aggregated treatment × species mechanistic signatures (RNS_m, ROS_m, *CAT1*_m, *GPX1*_m, *SOD1*_m (all means); Z-transformed within species). The tree selects *GPX1*_m (mean) as the primary split (≤ 0.545 vs > 0.545). High *GPX1*_m (Node 2) is strongly responder-enriched (90.3% responders; *n* = 31). Low *GPX1*_m (Node 1) is further partitioned by *CAT1*_m (threshold −1.435), yielding a pure responder node (Node 3, 100% responders; *n* = 11) and a mixed branch (Node 4, 63.0% responders; *n* = 54). Node 4 is refined by a second *GPX1*_m threshold (−0.190) to define a tolerant/resistant–enriched terminal node (Node 5, 85.7% tolerant/resistant; *n* = 7) versus a responder-leaning branch (Node 6, 70.2% responders; *n* = 47), which is finally split by *CAT1*_m (0.444) into Node 7 (82.8% responders; *n* = 29) and Node 8 (50% responders; *n* = 18). Node boxes report class proportions and sample sizes; "Improvement" values indicate the split contribution to classification. Right: Heatmap shows within-treatment distributions across terminal nodes (percent of each treatment's signatures assigned to each node; rows

sum to 100%). Treatments are ordered by decreasing overall signature strength (as labeled). The dashed bracket marks the resistance-enriched terminal node (Node 5); warmer colors indicate a higher fraction of that treatment's signatures mapping to a given node. Color scale: 0–100% of a treatment's signatures falling in each terminal node (blue = low; red = high).

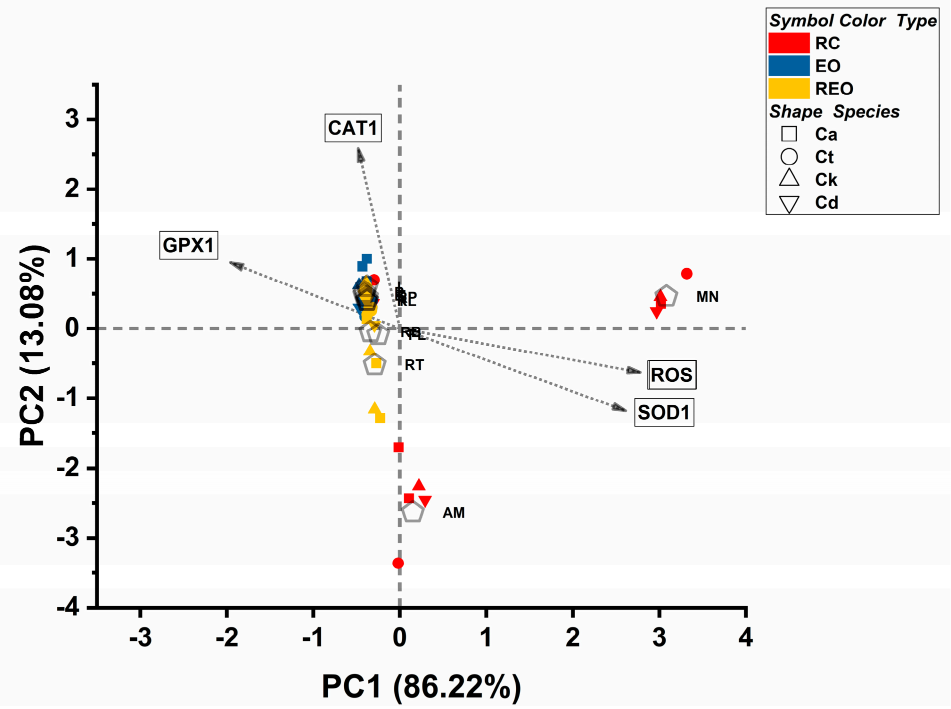

Figure S3. Global multivariate mode-of-action landscape separates stress-intensity and antioxidant-program axes across treatments and species.

Principal component analysis (PCA) was performed on aggregated treatment × species mechanistic signatures (RNS, ROS, *CAT1*, *GPX1*, *SOD1*; Z-transformed within species). The biplot shows PC1 (86.22%) versus PC2 (13.08%) for each treatment–species signature. Points are colored by treatment type (RC = reference controls, EO = essential oils, REO = RAMEB–EO complexes) and shaped by Candida species (Ca, Ct, Ck, Cd). Dashed crosshairs indicate the origin (0,0). Grey arrows depict rotated loading vectors: PC1 captures a dominant stress/redox intensity axis (positive direction aligned with ROS and *SOD1*, opposed to *GPX1*), whereas PC2 reflects a *CAT1*-centered antioxidant-response axis (positive direction aligned with *CAT1*). Centroid labels (treatment codes) indicate the mean position of each treatment across species. Hash marks (#) denote variables with the strongest contributions to the displayed axes (highest absolute loadings).

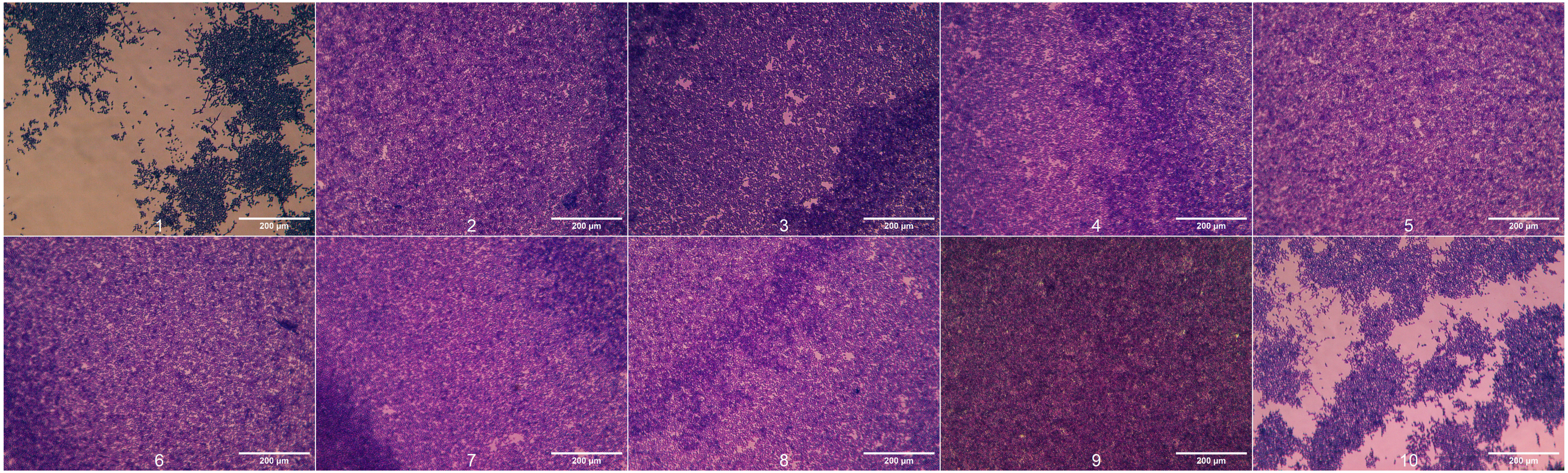

Figure S4. Representative crystal violet stained biofilm biomass images of *Candida albicans* (Ca1372) under treatment conditions.

Bright-field micrographs (20× magnification, scale: 200 µm) showing crystal violet-stained biofilms of *Candida albicans* strain 1372 following exposure to the experimental treatment panel. Biofilms were established under standardized conditions and subsequently treated with native essential oils (panel 3-6 representing L, B, P, T) and their corresponding RAMEB inclusion complexes (panel 7-10 representing RL, RB, RP, RT), alongside appropriate controls (panel 1-2 representing antifungal controls AM and FL, respectively). Crystal violet staining reflects total biofilm biomass, including adherent cells and extracellular matrix components. Differences in staining intensity and surface coverage qualitatively indicate treatment-dependent modulation of biofilm formation and structural integrity. Images are representative of independent experiments performed under identical conditions.

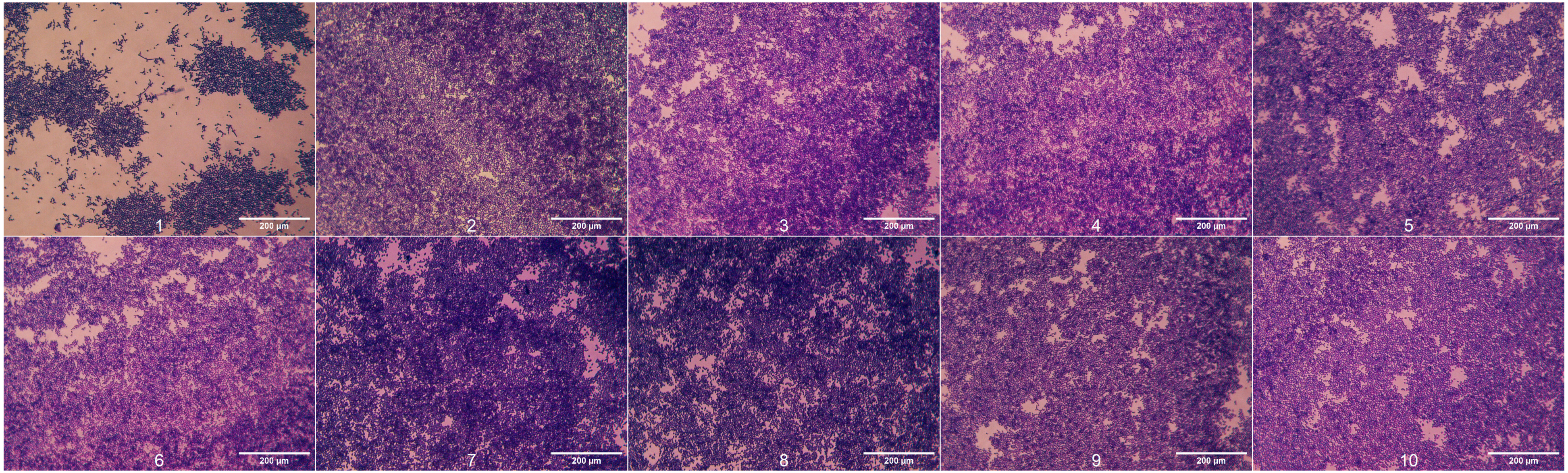

Figure S5. Representative crystal violet stained biofilm biomass images of *Candida albicans* (Ca1423) under treatment conditions.

Bright-field micrographs (20× magnification, scale: 200 µm) showing crystal violet-stained biofilms of *Candida albicans* strain 1423 following exposure to the experimental treatment panel. Biofilms were established under standardized conditions and subsequently treated with native essential oils (panel 3-6 representing L, B, P, T) and their corresponding RAMEB inclusion complexes (panel 7-10 representing RL, RB, RP, RT), alongside appropriate controls (panel 1-2 representing antifungal controls AM and FL, respectively). Crystal violet staining reflects total biofilm biomass, including adherent cells and extracellular matrix components. Differences in staining intensity and surface coverage qualitatively indicate treatment-dependent modulation of biofilm formation and structural integrity. Images are representative of independent experiments performed under identical conditions.

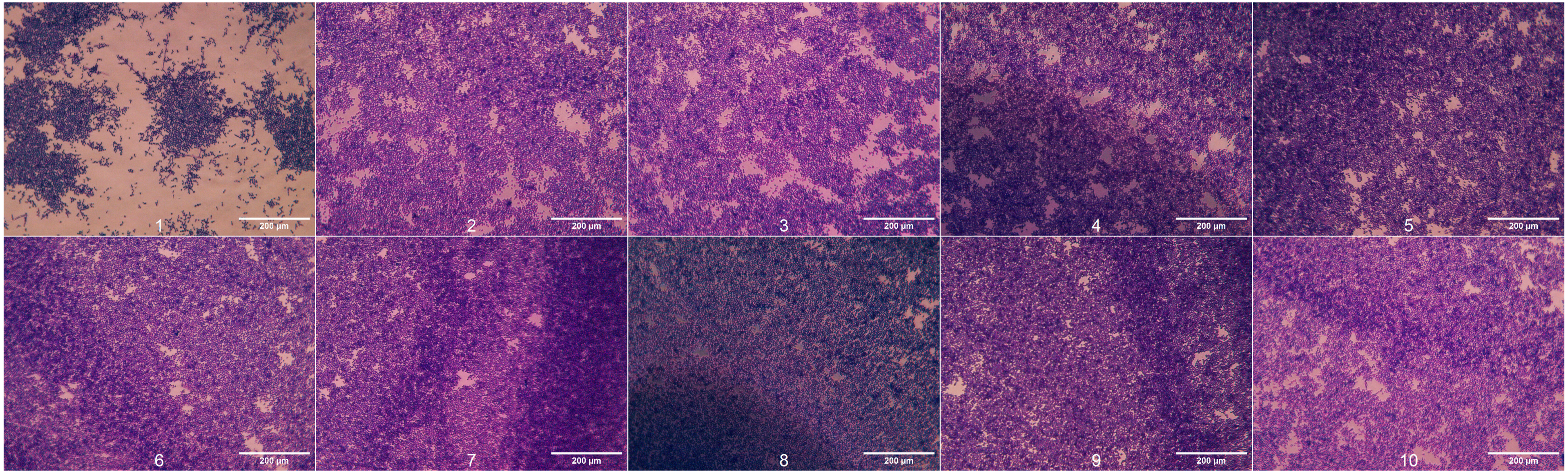

Figure S6. Representative crystal violet stained biofilm biomass images of *Candida albicans* (Ca1424) under treatment conditions.

Bright-field micrographs (20× magnification, scale: 200 µm) showing crystal violet-stained biofilms of *Candida albicans* strain 1424 following exposure to the experimental treatment panel. Biofilms were established under standardized conditions and subsequently treated with native essential oils (panel 3-6 representing L, B, P, T) and their corresponding RAMEB inclusion complexes (panel 7-10 representing RL, RB, RP, RT), alongside appropriate controls (panel 1-2 representing antifungal controls AM and FL, respectively). Crystal violet staining reflects total biofilm biomass, including adherent cells and extracellular matrix components. Differences in staining intensity and surface coverage qualitatively indicate treatment-dependent modulation of biofilm formation and structural integrity. Images are representative of independent experiments performed under identical conditions.

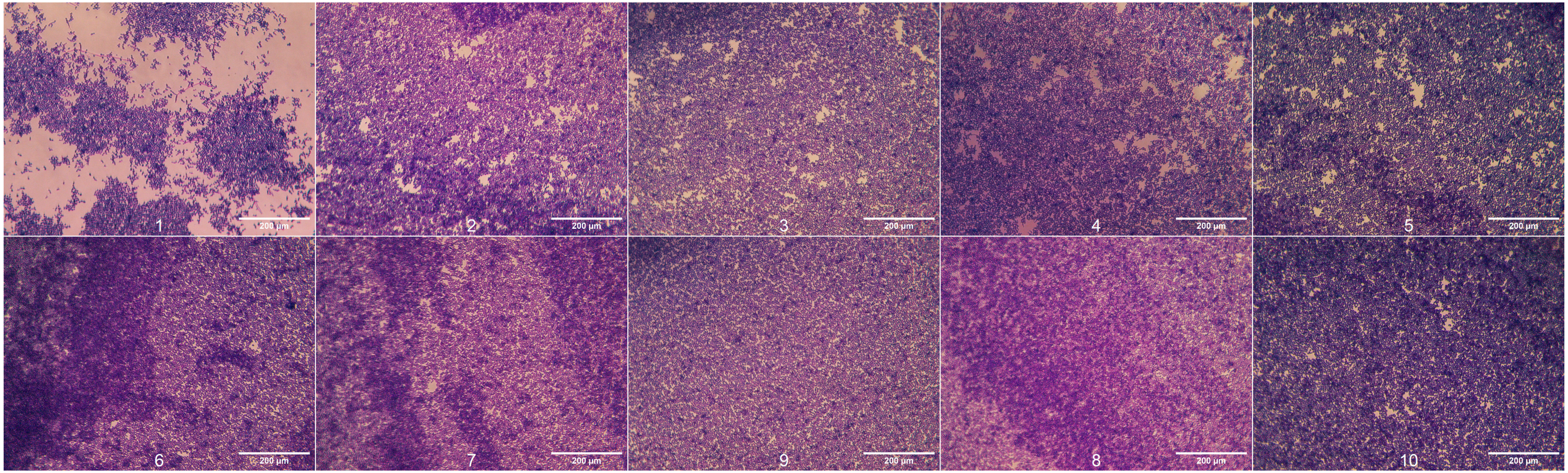

Figure S7. Representative crystal violet stained biofilm biomass images of *Candida tropicalis* (Ct1368) under treatment conditions.

Bright-field micrographs (20× magnification, scale: 200 µm) showing crystal violet-stained biofilms of *Candida tropicalis* strain 1368 following exposure to the experimental treatment panel. Biofilms were established under standardized conditions and subsequently treated with native essential oils (panel 3-6 representing L, B, P, T) and their corresponding RAMEB inclusion complexes (panel 7-10 representing RL, RB, RP, RT), alongside appropriate controls (panel 1-2 representing antifungal controls AM and FL, respectively). Crystal violet staining reflects total biofilm biomass, including adherent cells and extracellular matrix components. Differences in staining intensity and surface coverage qualitatively indicate treatment-dependent modulation of biofilm formation and structural integrity. Images are representative of independent experiments performed under identical conditions.

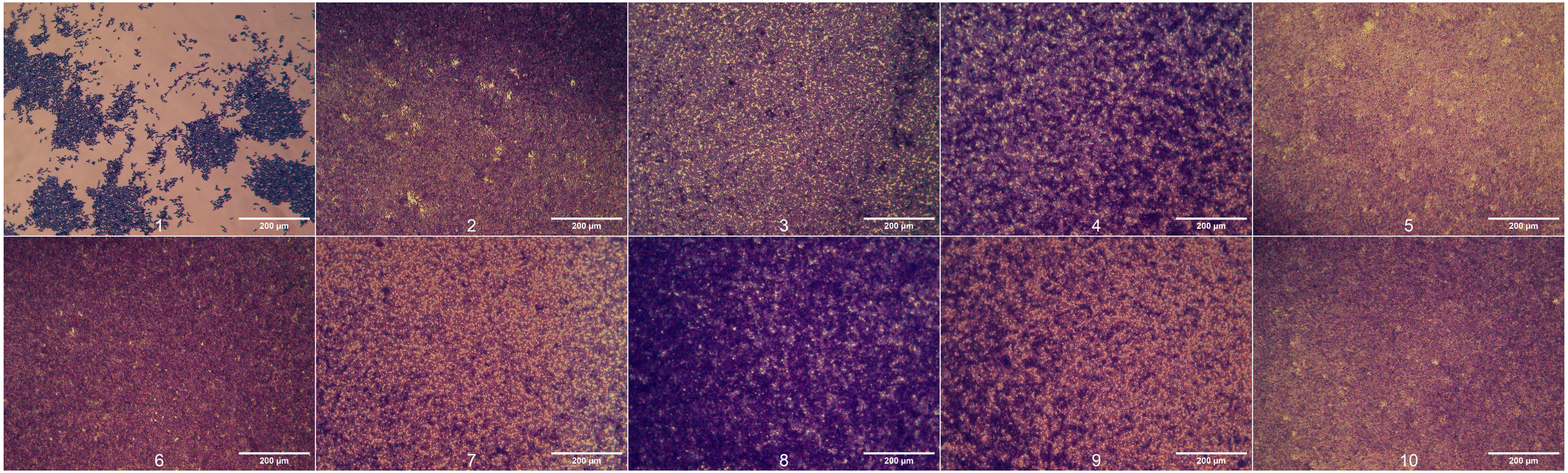

Figure S8. Representative crystal violet stained biofilm biomass images of *Candida tropicalis* (Ct1432) under treatment conditions.

Bright-field micrographs (20× magnification, scale: 200 µm) showing crystal violet-stained biofilms of *Candida tropicalis* strain 1432 following exposure to the experimental treatment panel. Biofilms were established under standardized conditions and subsequently treated with native essential oils (panel 3-6 representing L, B, P, T) and their corresponding RAMEB inclusion complexes (panel 7-10 representing RL, RB, RP, RT), alongside appropriate controls (panel 1-2 representing antifungal controls AM and FL, respectively). Crystal violet staining reflects total biofilm biomass, including adherent cells and extracellular matrix components. Differences in staining intensity and surface coverage qualitatively indicate treatment-dependent modulation of biofilm formation and structural integrity. Images are representative of independent experiments performed under identical conditions.

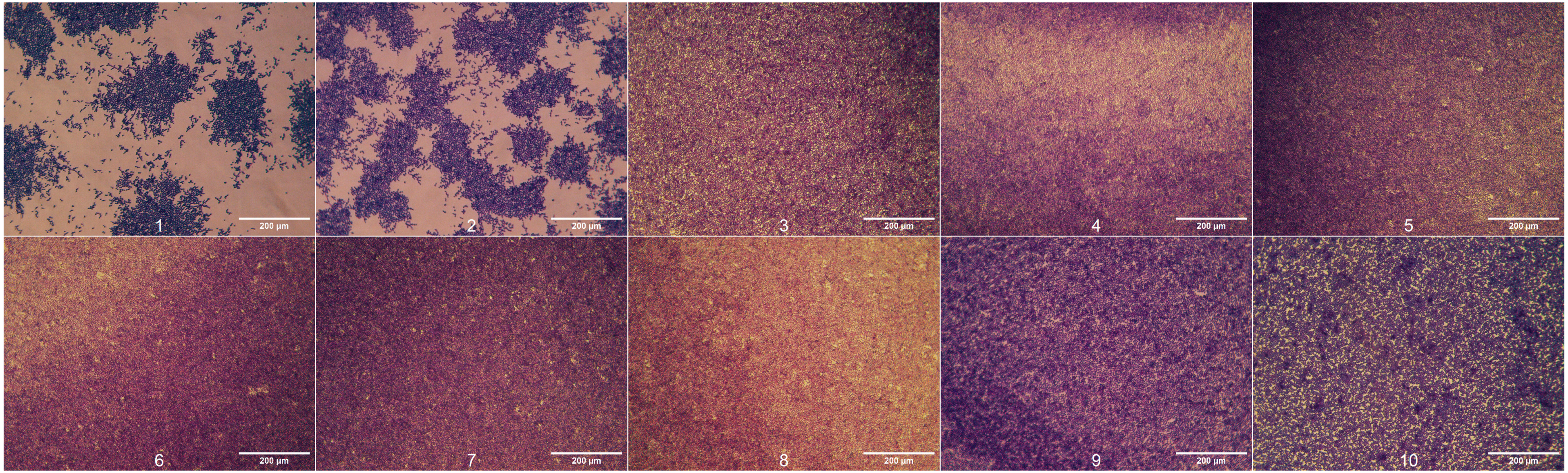

Figure S9. Representative crystal violet stained biofilm biomass images of *Candida krusei* (Ck779) under treatment conditions.

Bright-field micrographs (20× magnification, scale: 200 µm) showing crystal violet-stained biofilms of *Candida krusei* strain 779 following exposure to the experimental treatment panel. Biofilms were established under standardized conditions and subsequently treated with native essential oils (panel 3-6 representing L, B, P, T) and their corresponding RAMEB inclusion complexes (panel 7-10 representing RL, RB, RP, RT), alongside appropriate controls (panel 1-2 representing antifungal controls AM and FL, respectively). Crystal violet staining reflects total biofilm biomass, including adherent cells and extracellular matrix components. Differences in staining intensity and surface coverage qualitatively indicate treatment-dependent modulation of biofilm formation and structural integrity. Images are representative of independent experiments performed under identical conditions.

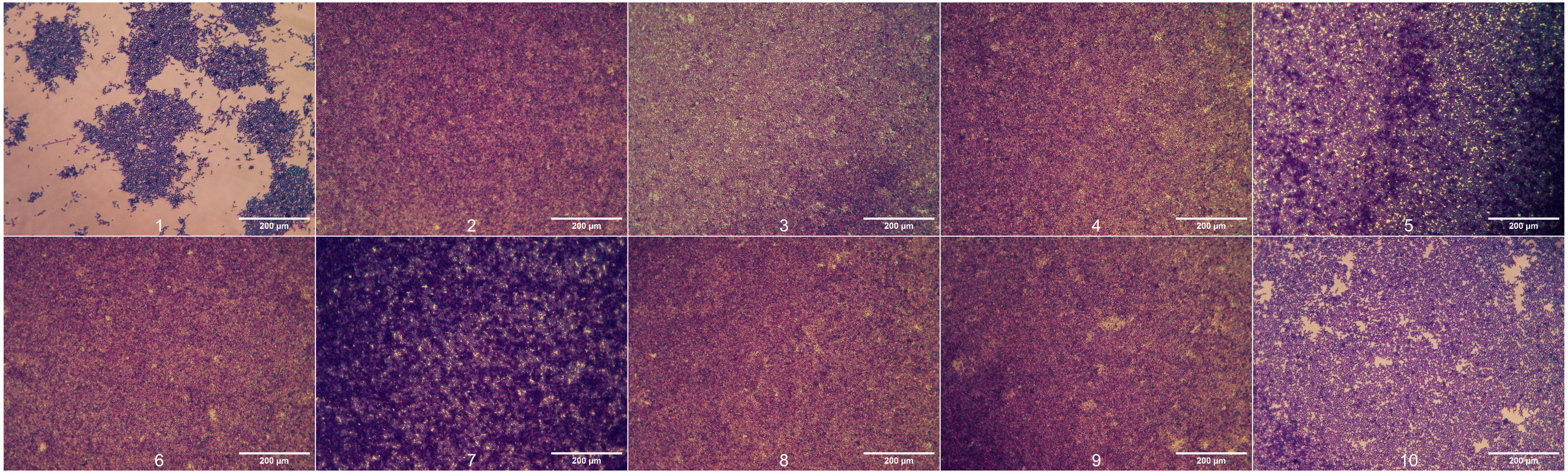

Figure S10. Representative crystal violet stained biofilm biomass images of *Candida krusei* (Ck1447) under treatment conditions.

Bright-field micrographs (20× magnification, scale: 200 µm) showing crystal violet-stained biofilms of *Candida krusei* strain 1447 following exposure to the experimental treatment panel. Biofilms were established under standardized conditions and subsequently treated with native essential oils (panel 3-6 representing L, B, P, T) and their corresponding RAMEB inclusion complexes (panel 7-10 representing RL, RB, RP, RT), alongside appropriate controls (panel 1-2 representing antifungal controls AM and FL, respectively). Crystal violet staining reflects total biofilm biomass, including adherent cells and extracellular matrix components. Differences in staining intensity and surface coverage qualitatively indicate treatment-dependent modulation of biofilm formation and structural integrity. Images are representative of independent experiments performed under identical conditions.

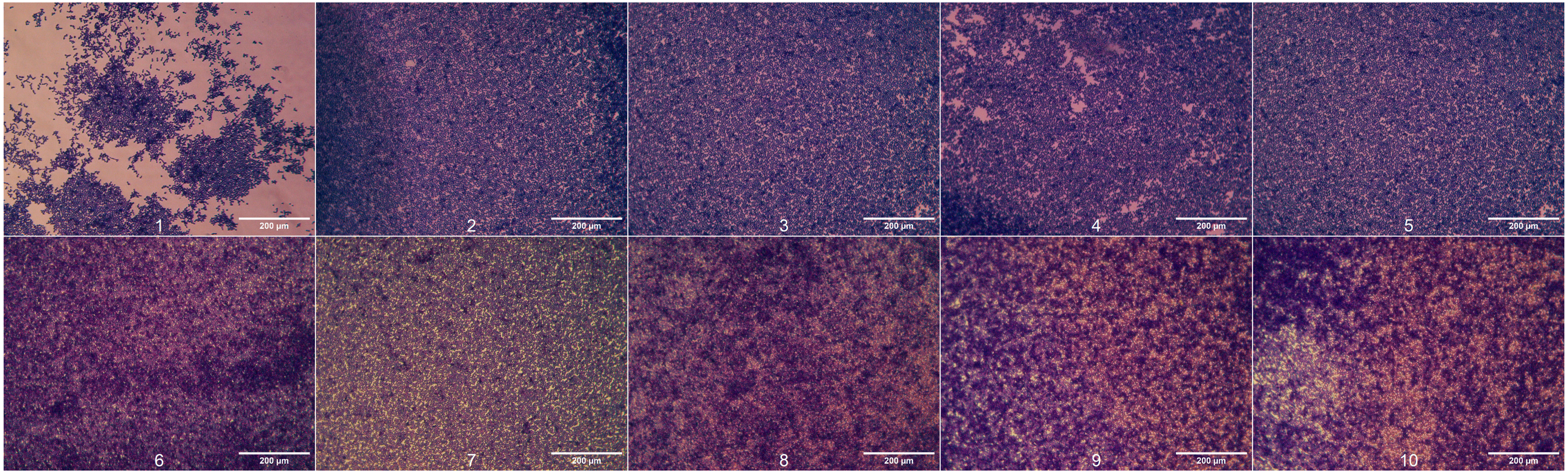

Figure S11. Representative crystal violet stained biofilm biomass images of *Candida dubliniensis* (Cd1470) under treatment conditions.

Bright-field micrographs (20× magnification, scale: 200 µm) showing crystal violet-stained biofilms of *Candida dubliniensis* strain 1470 following exposure to the experimental treatment panel. Biofilms were established under standardized conditions and subsequently treated with native essential oils (panel 3-6 representing L, B, P, T) and their corresponding RAMEB inclusion complexes (panel 7-10 representing RL, RB, RP, RT), alongside appropriate controls (panel 1-2 representing antifungal controls AM and FL, respectively). Crystal violet staining reflects total biofilm biomass, including adherent cells and extracellular matrix components. Differences in staining intensity and surface coverage qualitatively indicate treatment-dependent modulation of biofilm formation and structural integrity. Images are representative of independent experiments performed under identical conditions.

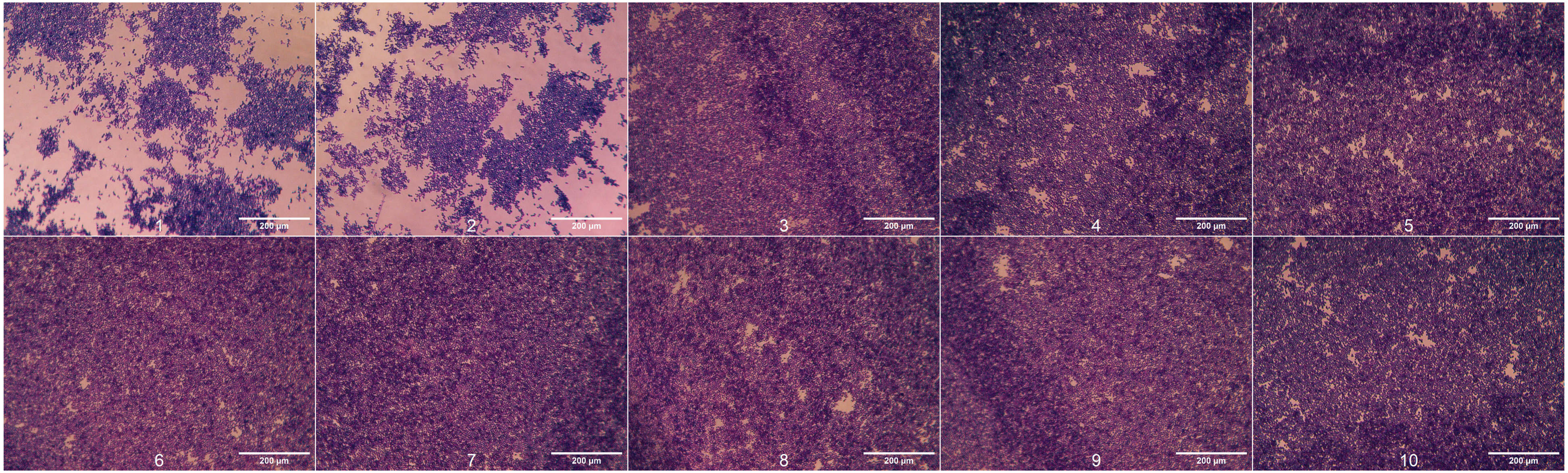

Figure S12. Representative crystal violet stained biofilm biomass images of *Candida dubliniensis* (Cd1471) under treatment conditions.

Bright-field micrographs (20× magnification, scale: 200 µm) showing crystal violet-stained biofilms of *Candida dubliniensis* strain 1471 following exposure to the experimental treatment panel. Biofilms were established under standardized conditions and subsequently treated with native essential oils (panel 3-6 representing L, B, P, T) and their corresponding RAMEB inclusion complexes (panel 7-10 representing RL, RB, RP, RT), alongside appropriate controls (panel 1-2 representing antifungal controls AM and FL, respectively). Crystal violet staining reflects total biofilm biomass, including adherent cells and extracellular matrix components. Differences in staining intensity and surface coverage qualitatively indicate treatment-dependent modulation of biofilm formation and structural integrity. Images are representative of independent experiments performed under identical conditions.

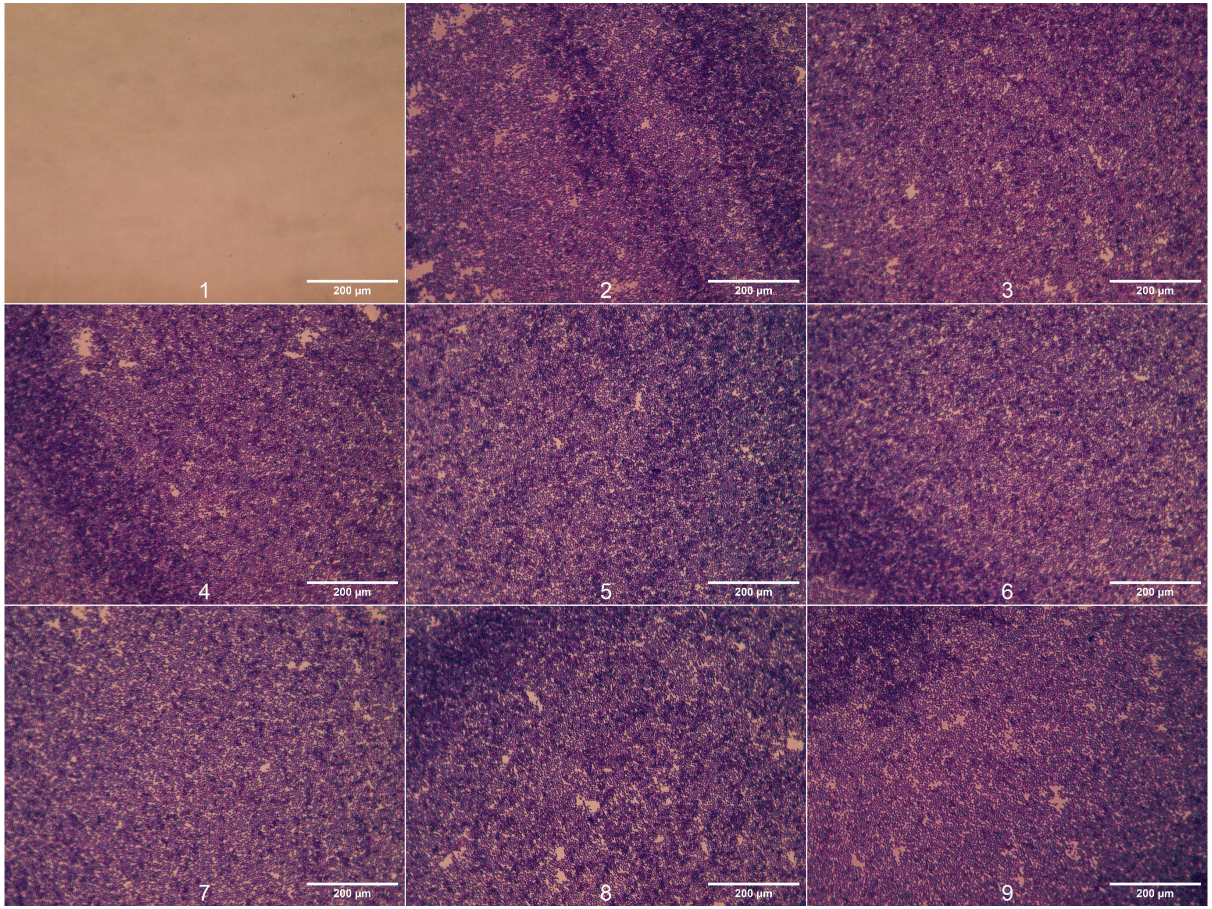

Figure S13. Representative crystal violet stained biofilm biomass images of background control (BC), untreated Candida species biofilm controls (UBCs).

Bright-field micrographs (20× magnification, scale: 200 μm) showing crystal violet-stained untreated biofilms controls (UBC) of tested Candida species. Panel 2-9 represents the *Candida albicans* (Ca1372, Ca1423, Ca1424), *Candida tropicalis* (Ct1368, Ct1432), *Candida krusei* (Ck779, Ck1447) and *Candida dubliniensis* (Cd1470, Cd1471), whereas the panel 1 represents the background noise control (NC), respectively. Crystal violet staining reflects total biofilm biomass, including adherent cells and extracellular matrix components. Differences in staining intensity and surface coverage qualitatively indicate treatment-dependent modulation of biofilm formation and structural integrity. Images are representative of independent experiments performed under identical conditions.

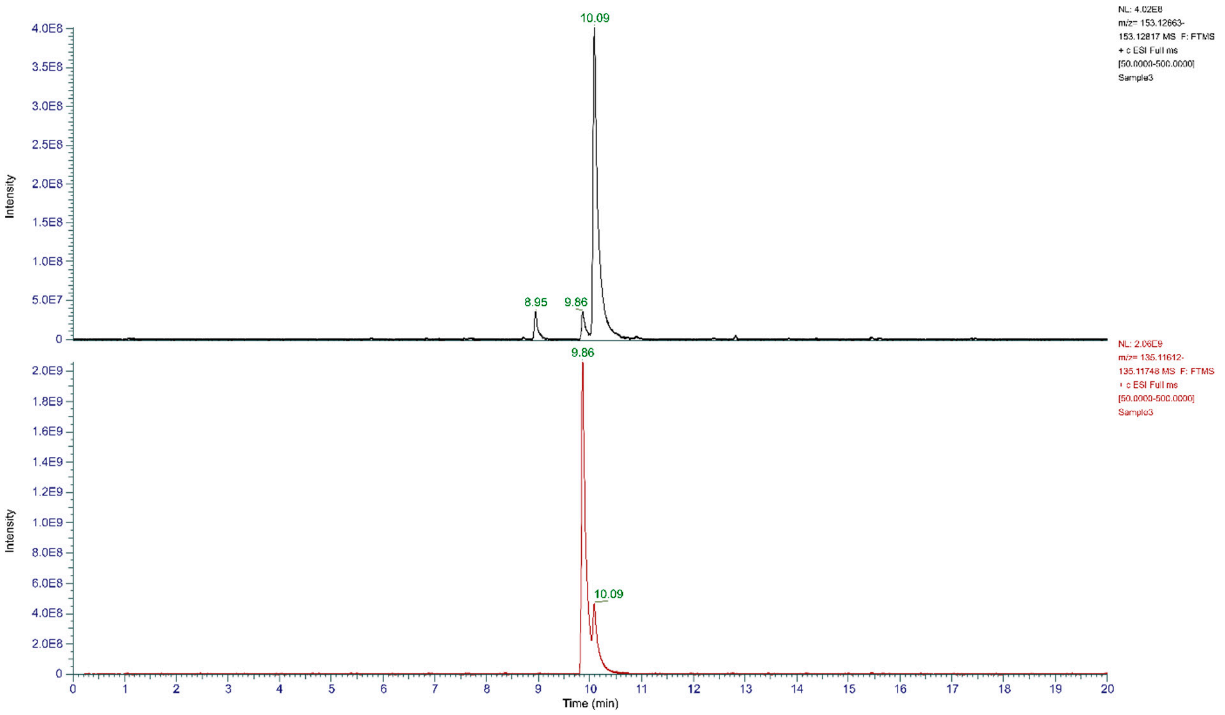

Figure S14. Lemon balm RIC chromatogram: In the upper panel, citral [M+H]+; in the lower panel, citral [M+H−H_2O]+.

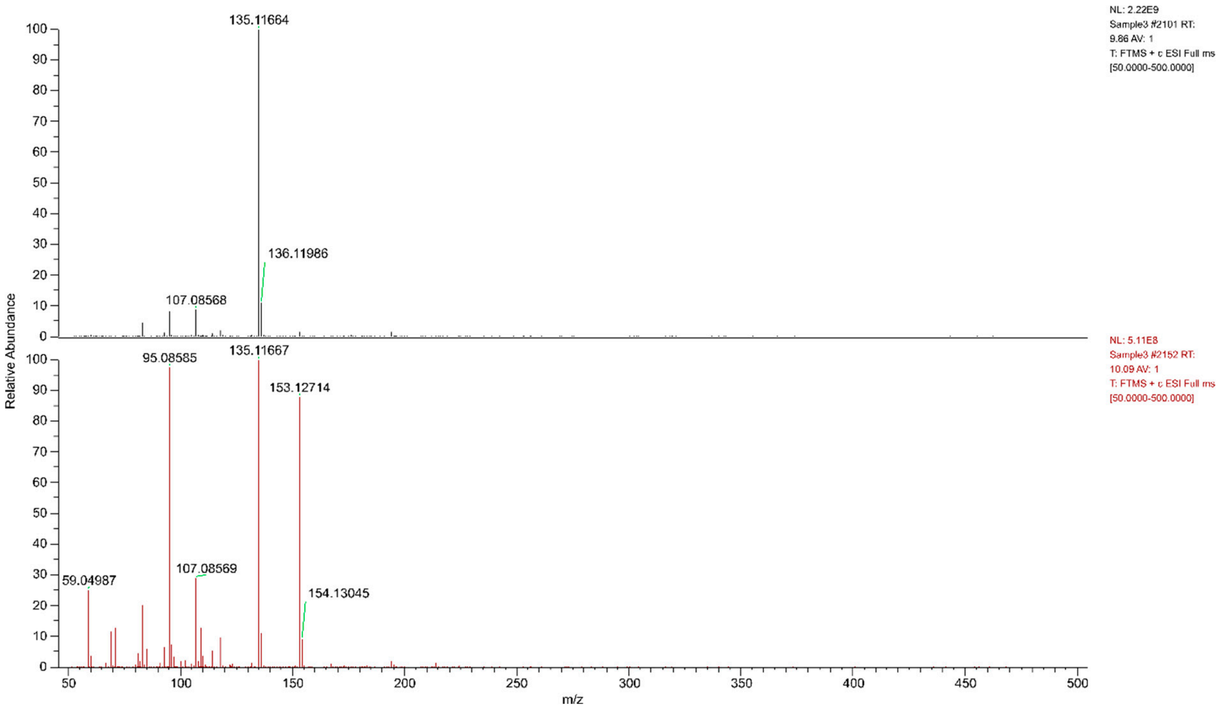

Figure S15. Mass spectrum of the lemon balm oil sample. Upper panel: peak spectrum at tR 9.86; lower panel: peak spectrum at t_R 10.09.

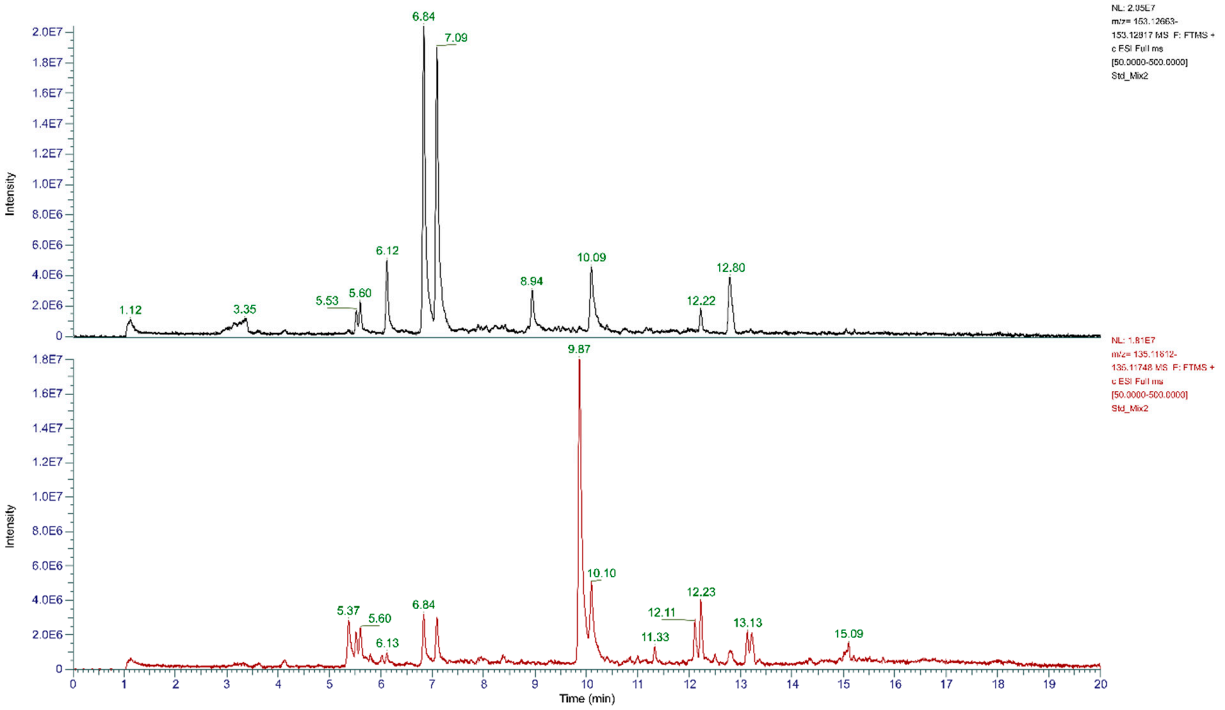

Figure S16. EIC chromatogram of the citral standard. In the upper panel, citral [M+H]+; in the lower panel, citral [M+H−H_2O]+.

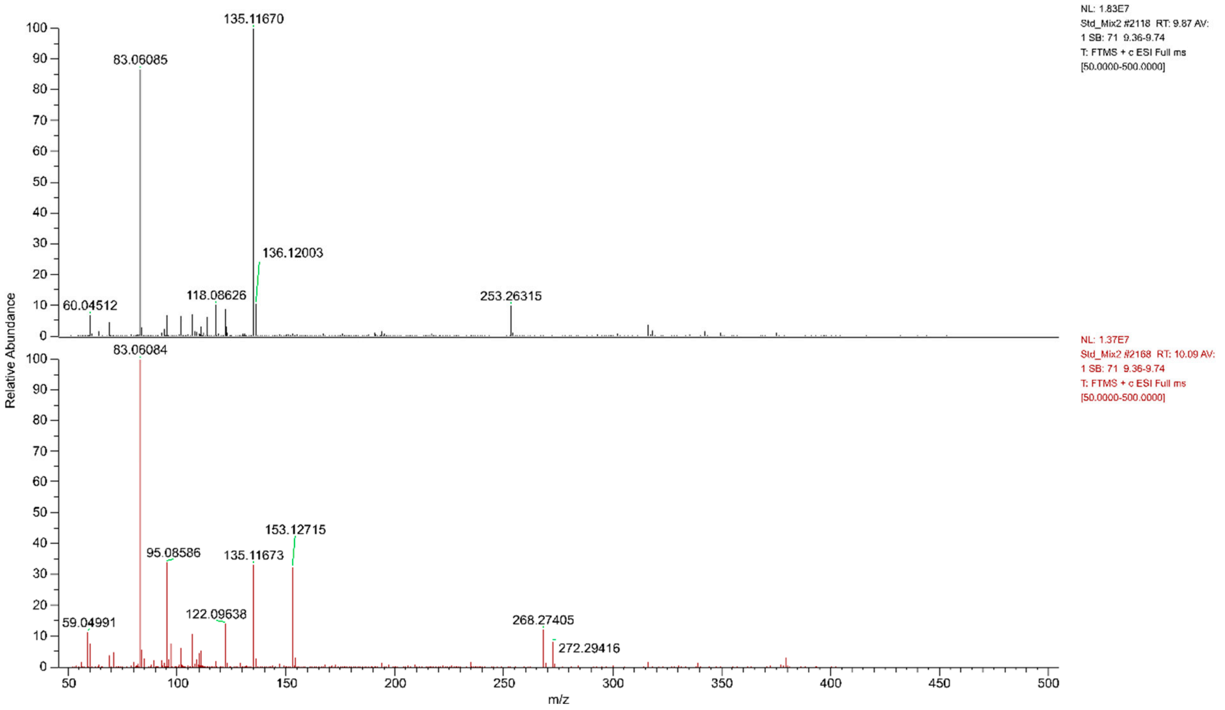

Figure S17. Mass spectrum of the citral standard. Upper panel: peak spectrum at tR 9.87; lower panel: peak spectrum at t_R 10.10.

Table S12. HPLC-HRMS-based annotation of major and minor constituents detected in lemon balm essential oil

#	Compound	t_R (min)		m/z	Area	Total area	Area %
1	Hexyl acetate	1.25	$[M+H]^+$	145.1223	0	966160	0.0044
			$[M+H-H_2O]^+$	127.1117	0		
			$[M+H-CH_3COOH]^+$	85.1012	966160		
2	Eucalyptol	7.37	$[M+H]^+$	155.1430	0	377723	0.0017
			$[M+H-H_2O]^+$	137.1325	377723		
3	Methyl heptenone	8.54	$[M+H]^+$	127.1117	151165242	374050703	1.6878
			$[M+H-H_2O]^+$	109.1012	222885461		
4	Citronellal	8.72	$[M+H]^+$	155.1430	0	1858047	0.0084
			$[M+H-H_2O]^+$	137.1325	1858047		
5	Camphene	8.90	$[M+H]^+$	153.1274	173948203	173948203	0.7849
6	Camphor	9.08	$[M+H]^+$	153.1274	0	12051792	0.0544
			$[M+H-H_2O]^+$	135.1168	12051792		
7	Terpinen-4-ol	9.27	$[M+H]^+$	155.1430	0	10911812	0.0492
			$[M+H-H_2O]^+$	137.1325	10911812		
8	Linalool* (unknown alcohol or Linalool isomer)	9.46	$[M+H]^+$	155.1430	1219637	1609839781	7.2639
			$[M+H-H_2O]^+$	137.1325	1608620144		
9	α-Terpineol	9.63	$[M+H]^+$	155.1430	0	442069948	1.9947
			$[M+H-H_2O]^+$	137.1325	442069948		
10	Neral (β-citral)	9.87	$[M+H]^+$	153.1274	193171285	11796621128	53.2288
			$[M+H-H_2O]^+$	135.1168	11603449843		
11	Geranial (α-citral)	10.10	$[M+H]^+$	153.1274	2723139642	6309163767	28.4683
			$[M+H-H_2O]^+$	135.1168	3586024125		
12	Linalyl acetate	10.51	$[M+H]^+$	197.1536	3203584	3203584	0.0145
			$[M+H-H_2O]^+$	179.1430	0		
13	Geranyl acetate	10.90	$[M+H]^+$	197.1536	998893	998893	0.0045

			$[M+H-H_2O]^+$	179.1430	0		
14	Borneol	11.40	$[M+H]^+$	155.1430	1767895	8008079	0.0361
			$[M+H-H_2O]^+$	137.1325	6240184		
15	β-Myrcene	12.26	$[M+H]^+$	137.1325	812518589	812518589	3.6663
16	Isocaryophyllene	12.89	$[M+H]^+$	205.1951	585303942	585303942	2.6410
17	Cis-β-ocimene	15.38	$[M+H]^+$	137.1325	6284754	6284754	0.0284
18	Limonene	15.50	$[M+H]^+$	137.1325	13910338	13910338	0.0628

The t_R , retention time (min); m/z, mass-to-charge ratio; Area, integrated peak area; Total area, sum of corresponding ion signals; Area %, relative contribution to total ion current. [M+H]+ denotes protonated molecular ion; fragment ions such as $[M+H-H_2O]+$ and $[M+H-CH_3COOH]+$ represent characteristic in-source or MS/MS-derived fragments used for compound annotation. Compound identification was based on accurate mass measurements and fragmentation patterns obtained under positive HESI conditions and compared with literature and reference data. Assignments of minor components and isomeric compounds (e.g., linalool/isomer) should be considered tentative. Relative abundances are semi-quantitative and do not represent absolute concentrations.

pharmaceutics MDPI

Supplementary Method

SM1. HPLC-MS analysis of lavender essential oil (L)

Sample preparation

Essential oil samples were diluted 1:1000 (v/v) in 2-propanol prior to analysis. A reference standard solution was prepared at a concentration of 20 µg/mL in 2-propanol and used for system suitability and signal comparison.

HPLC-HRMS instrumentation and chromatographic conditions

Chromatographic separation was performed using a Thermo Dionex Ultimate 3000 UHPLC system (Dionex, Sunnyvale, CA, USA) coupled to a Thermo Q Exactive Focus quadrupole-Orbitrap high-resolution mass spectrometer (Thermo Fisher Scientific, Waltham, MA, USA). Separation was achieved on a Thermo Accucore RP-MS column (150 mm × 2.1 mm, 2.6 µm particle size) fitted with a corresponding Accucore guard column (5 mm × 2.1 mm, 2.6 µm). The column temperature was maintained at 40 °C, while the autosampler tray was operated under ambient conditions. The mobile phase consisted of a binary solvent system, namely, water containing 0.1% (v/v) formic acid (A) and acetonitrile containing 0.1% (v/v) formic acid (B). Elution was carried out using a gradient program as detailed in Table S11. The flow rate was set at 0.4 mL/min, and the injection volume was 2 µL.

Table S13. HPLC-HRMS gradient program

Time (min)	A (%)	B (%)
0.0	90	10
1.0	90	10
15.0	5	95
20.0	5	95
22.1	90	10
25.0	90	10

Mass spectrometric conditions

Mass spectrometric detection was performed in positive ionization mode using heated electrospray ionization (HESI). Full-scan mass spectra (FullMS-ddMS^2) were acquired over an m/z range of 50-500 at a resolution of 70,000 (FWHM at m/z 200). Data-dependent MS/MS fragmentation was conducted at a resolution of 17,500 using an isolation window of 1.0 m/z and stepped normalized collision energies (NCE) of 20, 40, and 60. The ion source parameters were set as follows: spray voltage 3.50 kV, capillary temperature 300 °C, sheath gas flow rate 30 arbitrary units (AU), auxiliary gas flow rate 10 AU, and auxiliary gas temperature 350 °C.

Data processing

All the data acquisition and evaluation were conducted using Xcalibur software (version 4.2) and Q Exactive Focus software (version 2.1, Thermo Fisher Scientific). Compound annotation was based on accurate mass measurements and fragmentation patterns in comparison with available reference data and literature reports [31,32]

 https://doi.org/10.3390/pharmaceutics18040508
