# Supplementary figures and images for: Species-Specific Susceptibility of Planktonic and Biofilm Forming Candida Strains to Cyclodextrin-Encapsulated Essential Oils
Source: Pharmaceutics. 2026 Apr 20;18(4):508. doi: 10.3390/pharmaceutics18040508 (PMC13119200; doi:10.3390/pharmaceutics18040508)

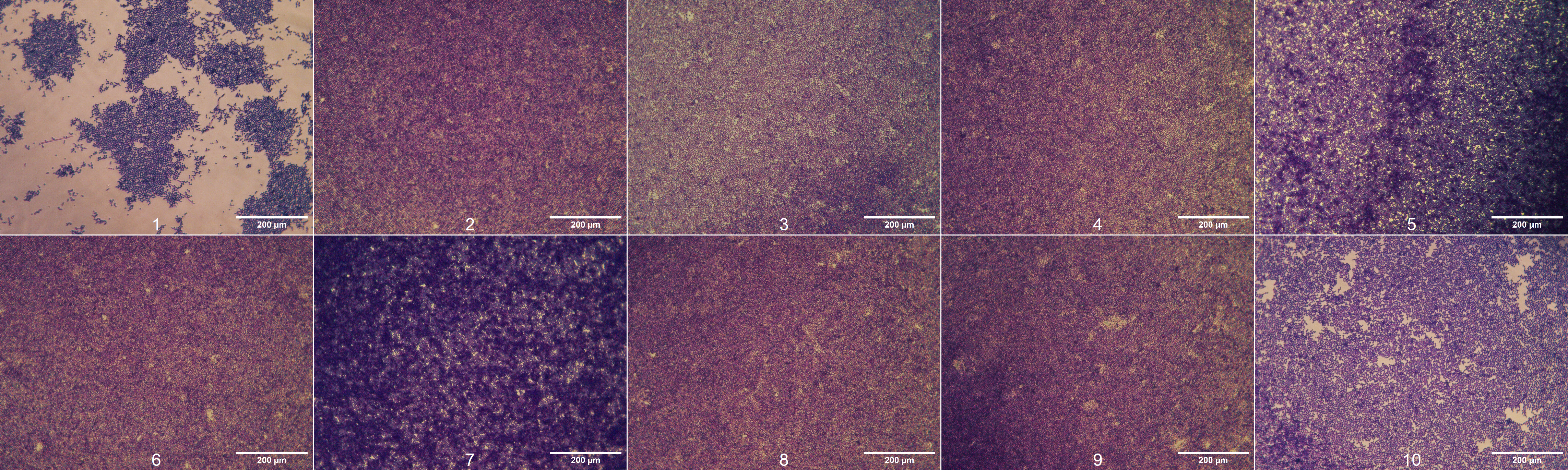

Supplement: Supplementary file 1 [file pharmaceutics-18-00508-s001.zip › Supplementary figures/Figure S10.tif]

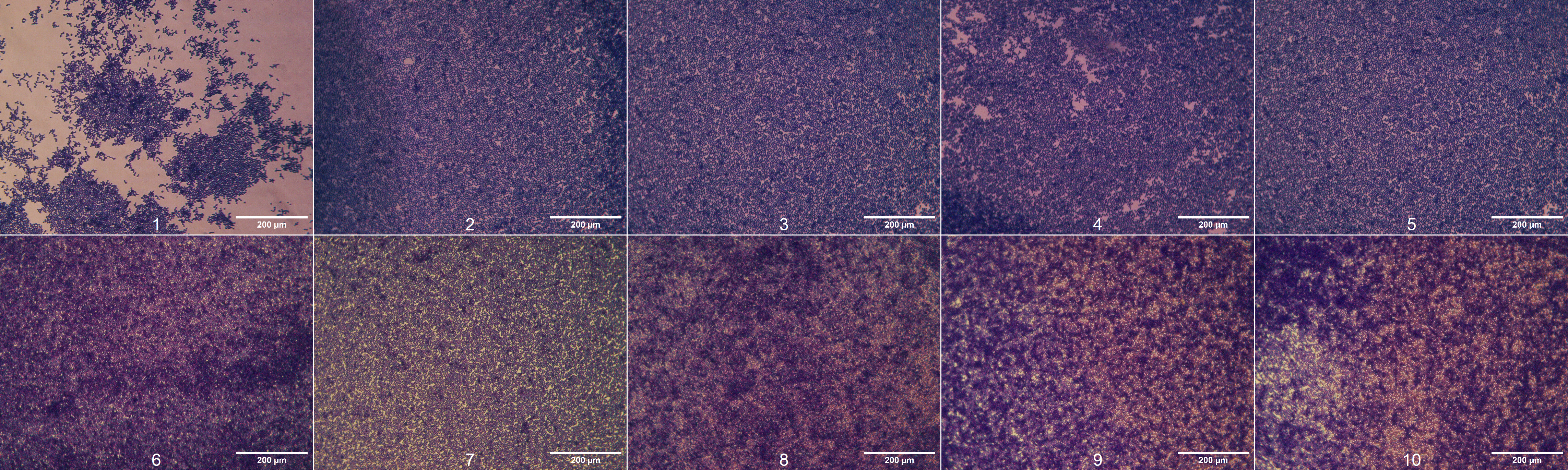

Supplement: Supplementary file 1 [file pharmaceutics-18-00508-s001.zip › Supplementary figures/Figure S11.tif]

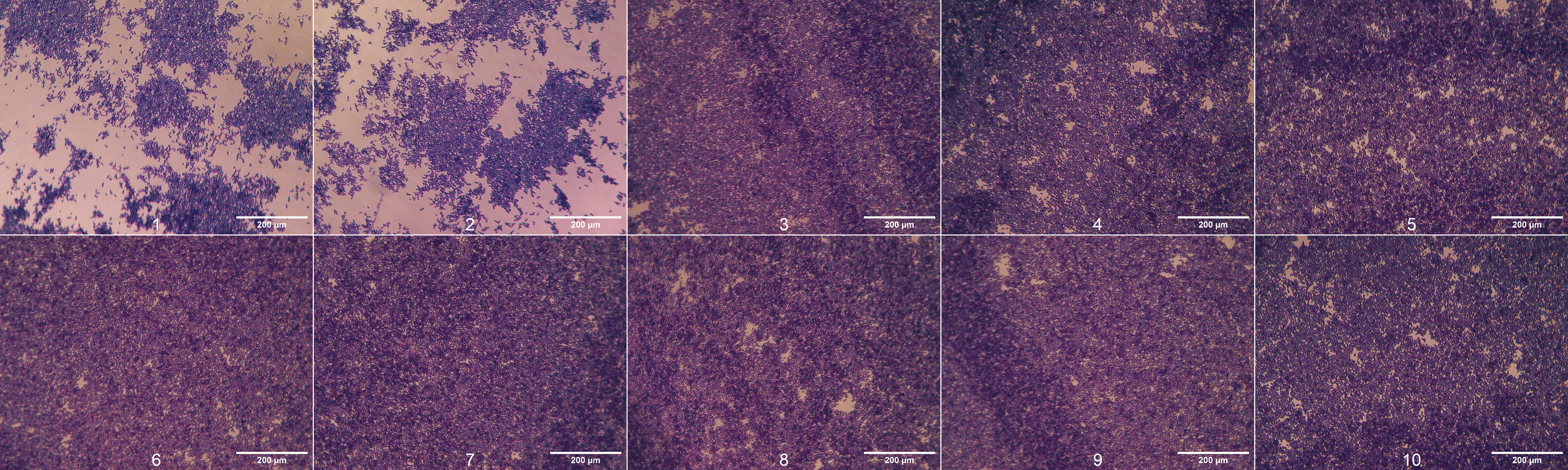

Supplement: Supplementary file 1 [file pharmaceutics-18-00508-s001.zip › Supplementary figures/Figure S12.tif]

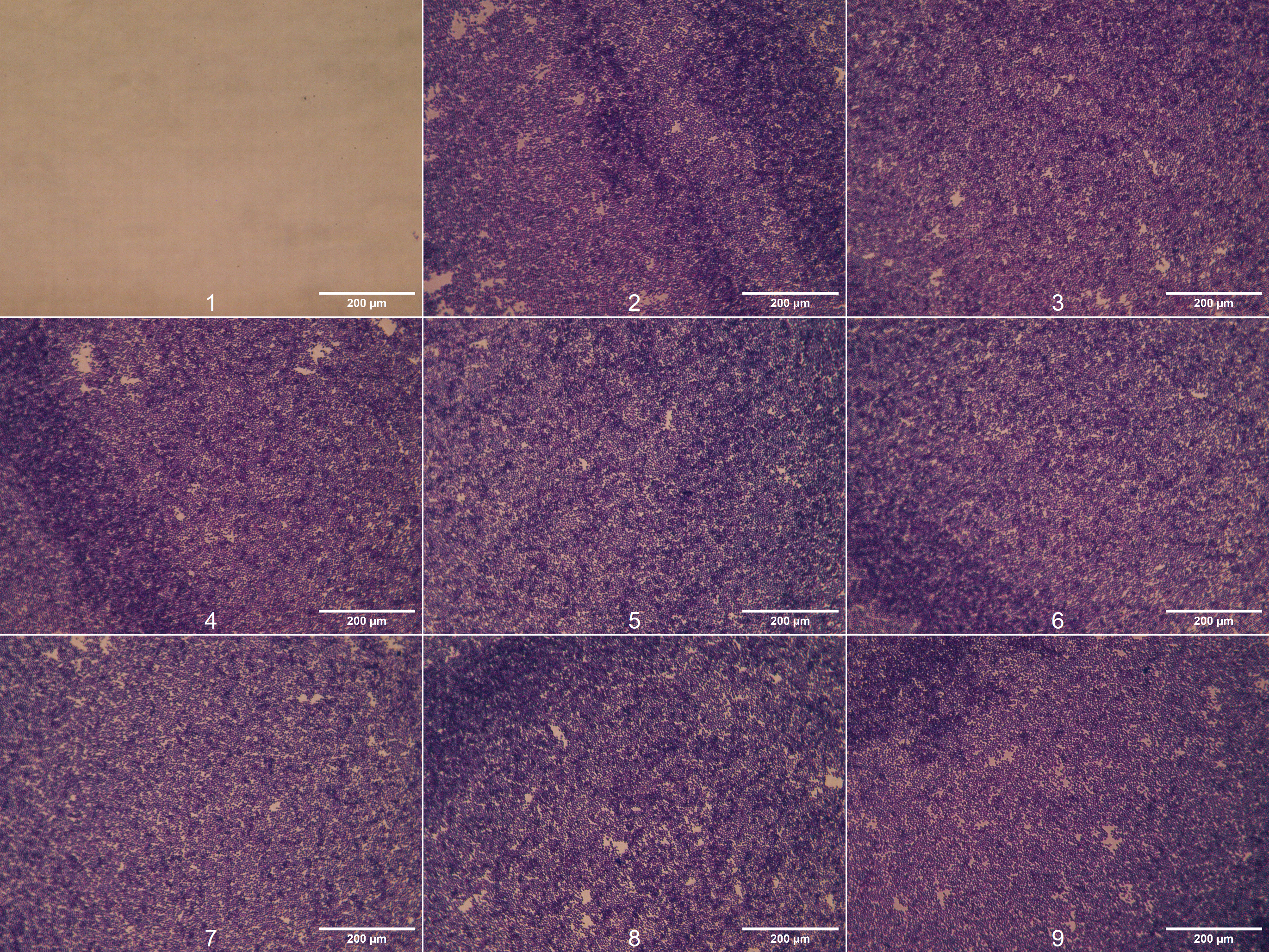

Supplement: Supplementary file 1 [file pharmaceutics-18-00508-s001.zip › Supplementary figures/Figure S13.tif]

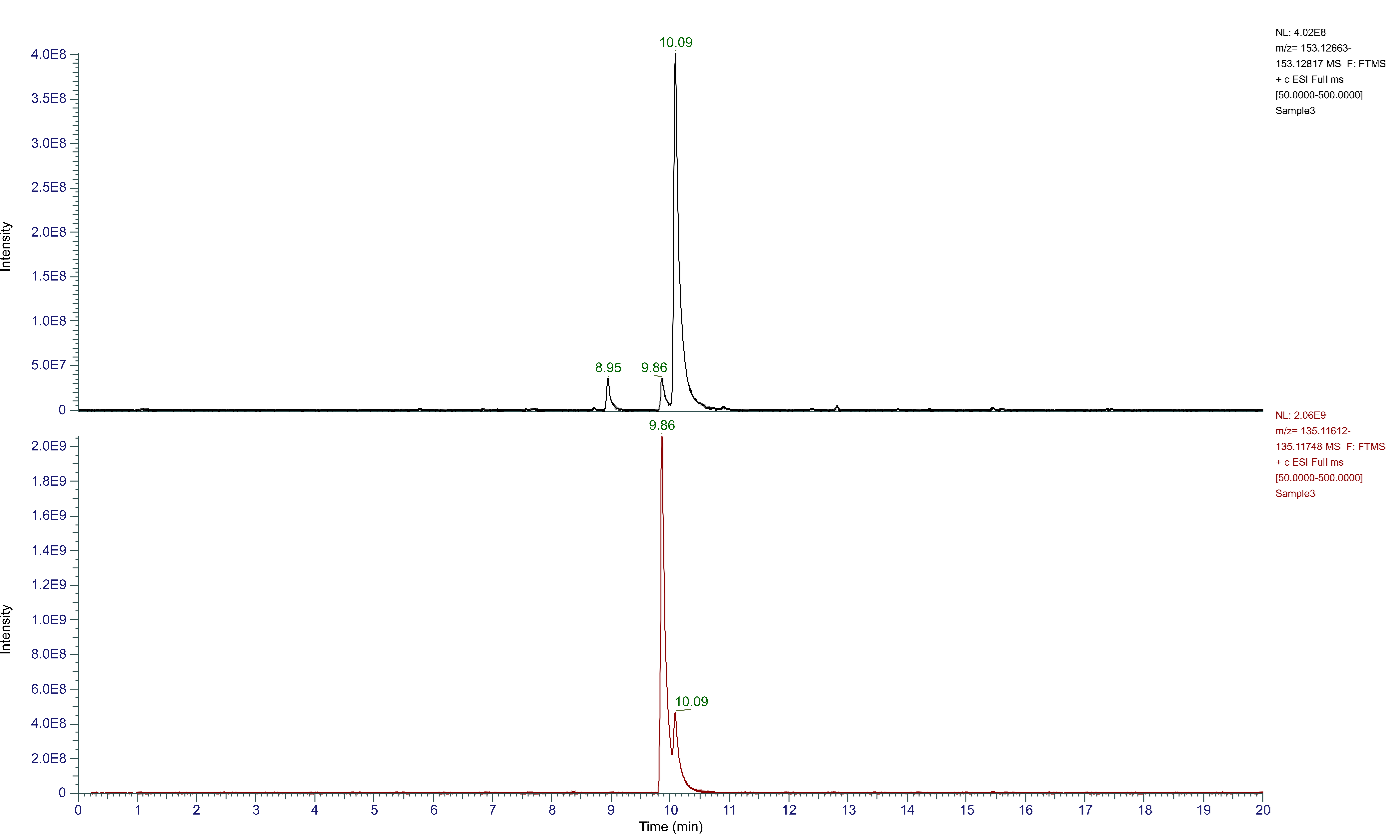

Supplement: Supplementary file 1 [file pharmaceutics-18-00508-s001.zip › Supplementary figures/Figure S14.tif]

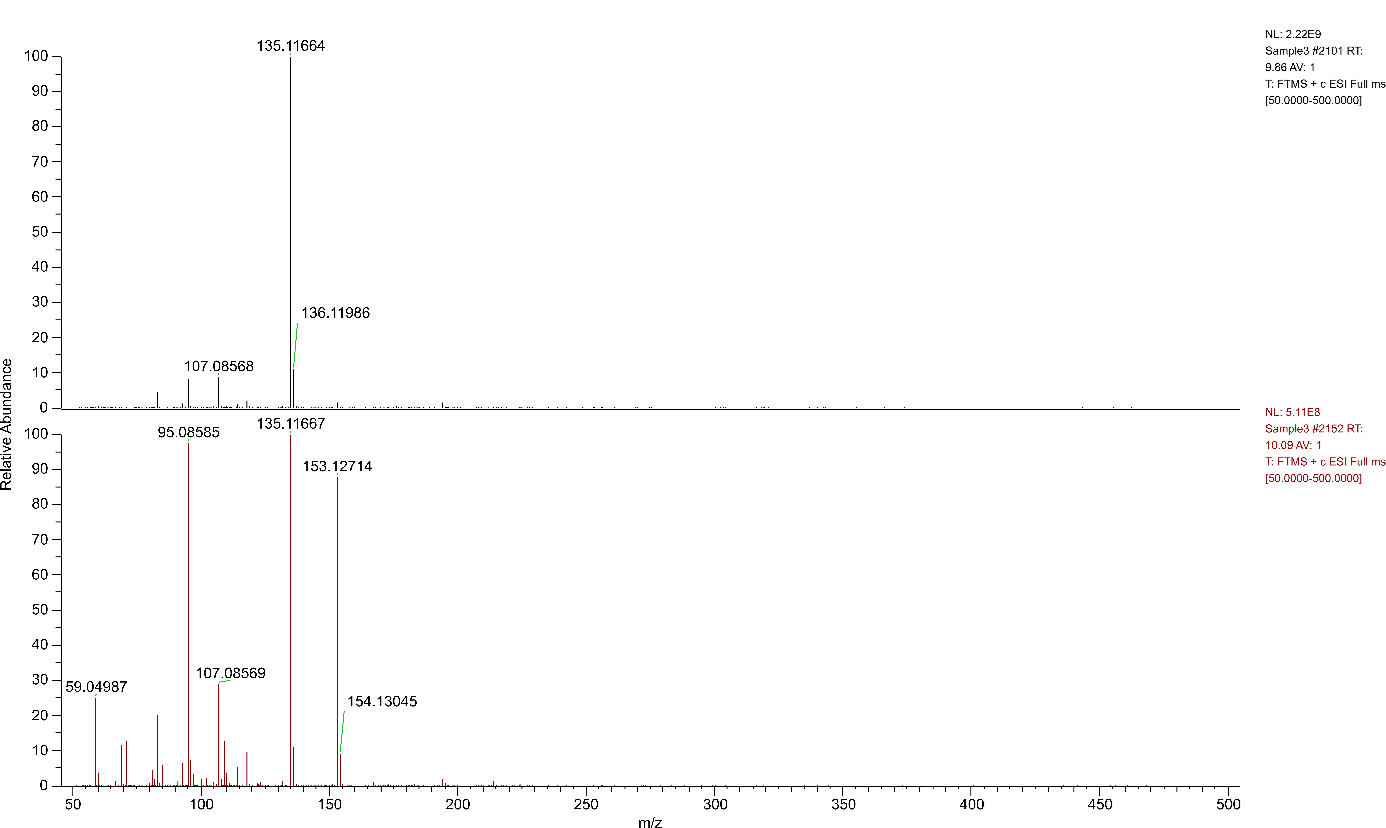

Supplement: Supplementary file 1 [file pharmaceutics-18-00508-s001.zip › Supplementary figures/Figure S15.tif]

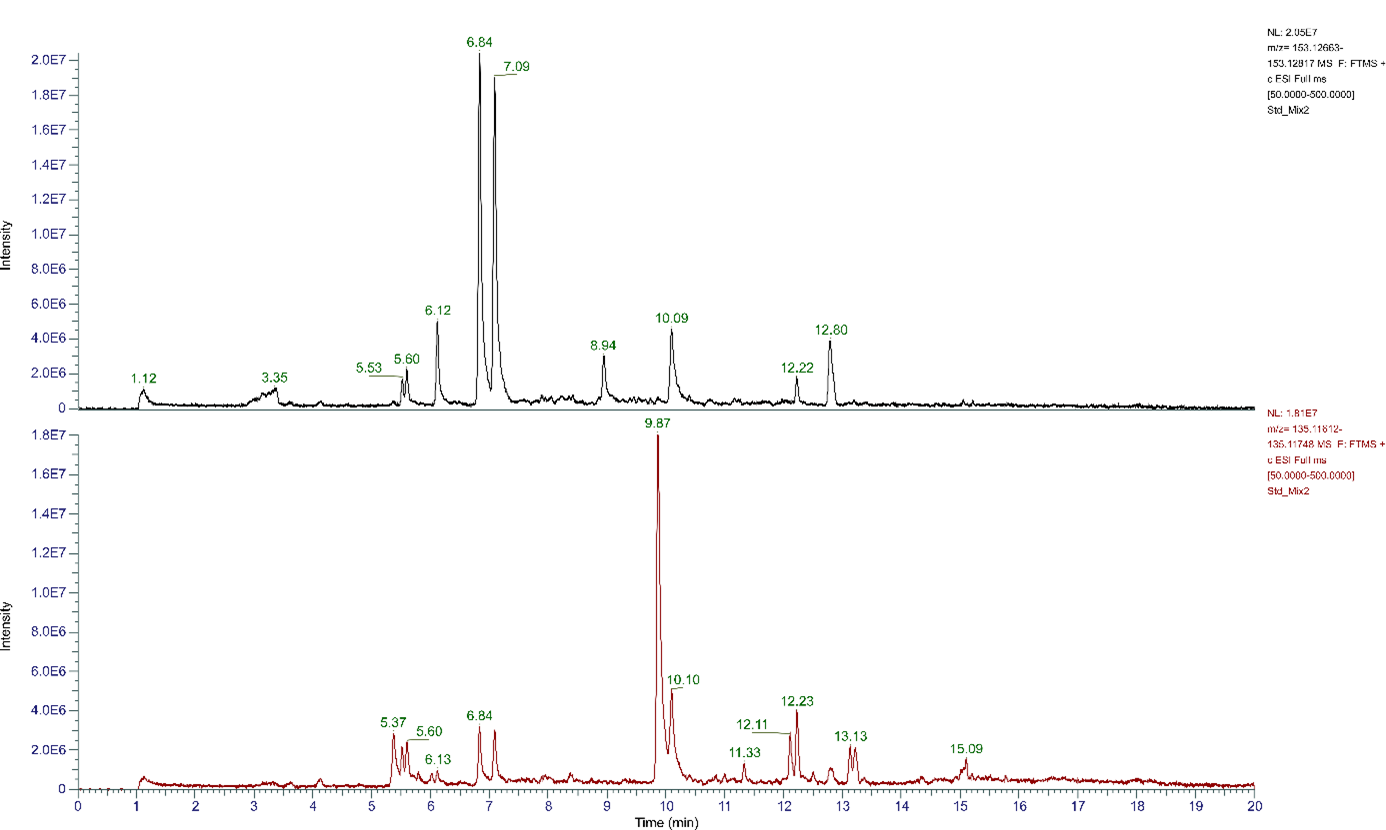

Supplement: Supplementary file 1 [file pharmaceutics-18-00508-s001.zip › Supplementary figures/Figure S16.tif]

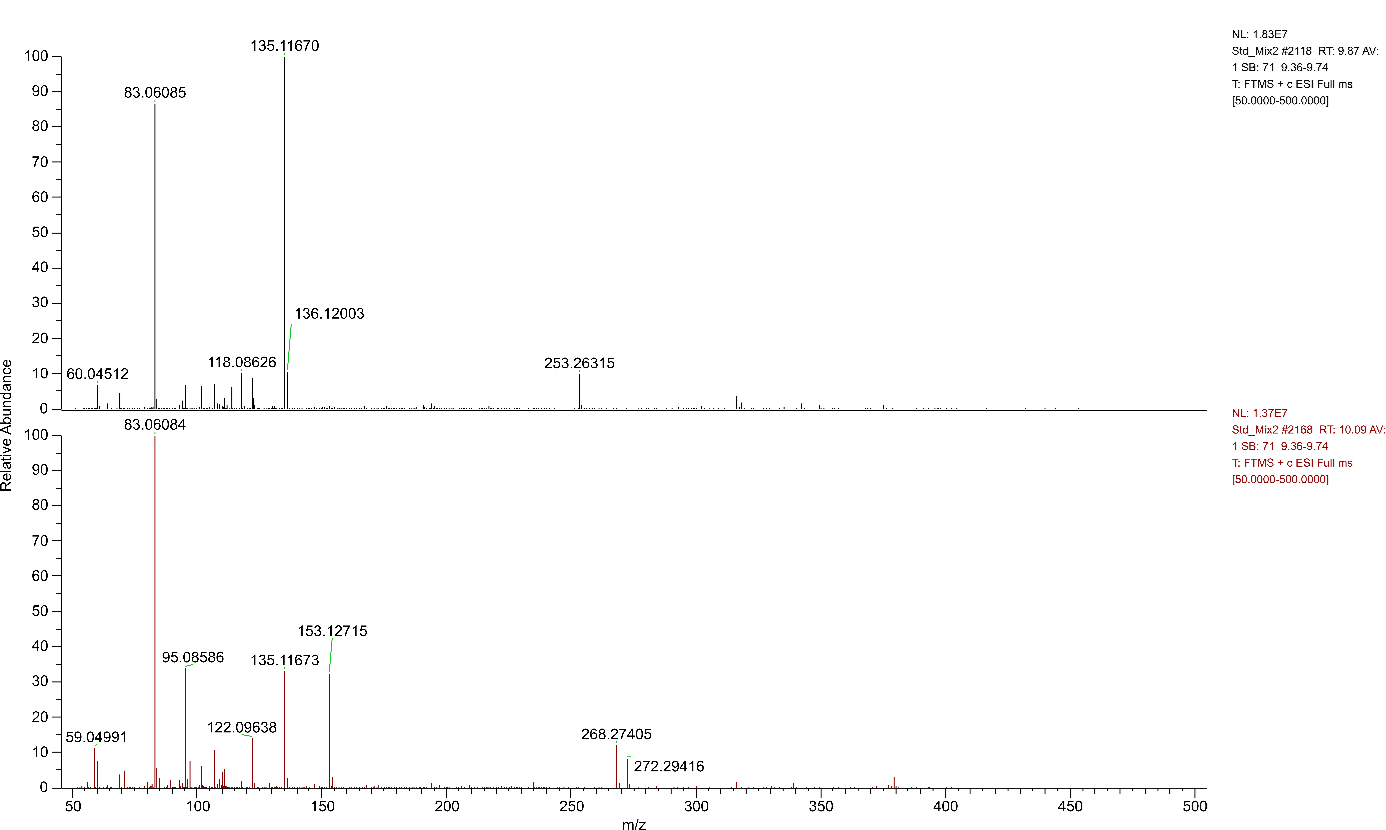

Supplement: Supplementary file 1 [file pharmaceutics-18-00508-s001.zip › Supplementary figures/Figure S17.tif]

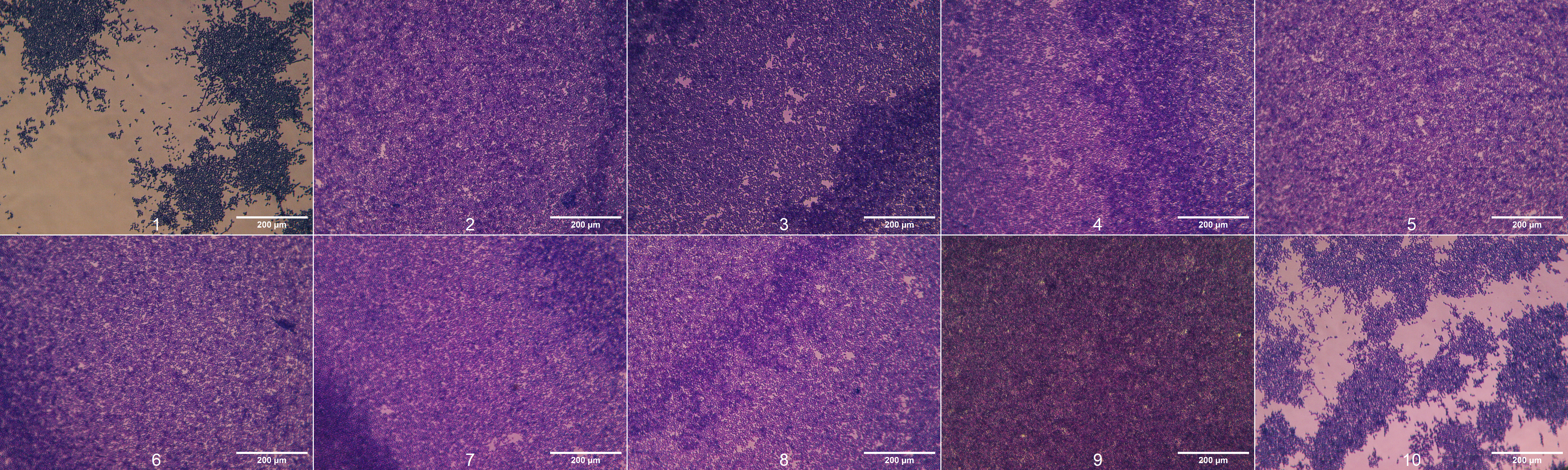

Supplement: Supplementary file 1 [file pharmaceutics-18-00508-s001.zip › Supplementary figures/Figure S4.tif]

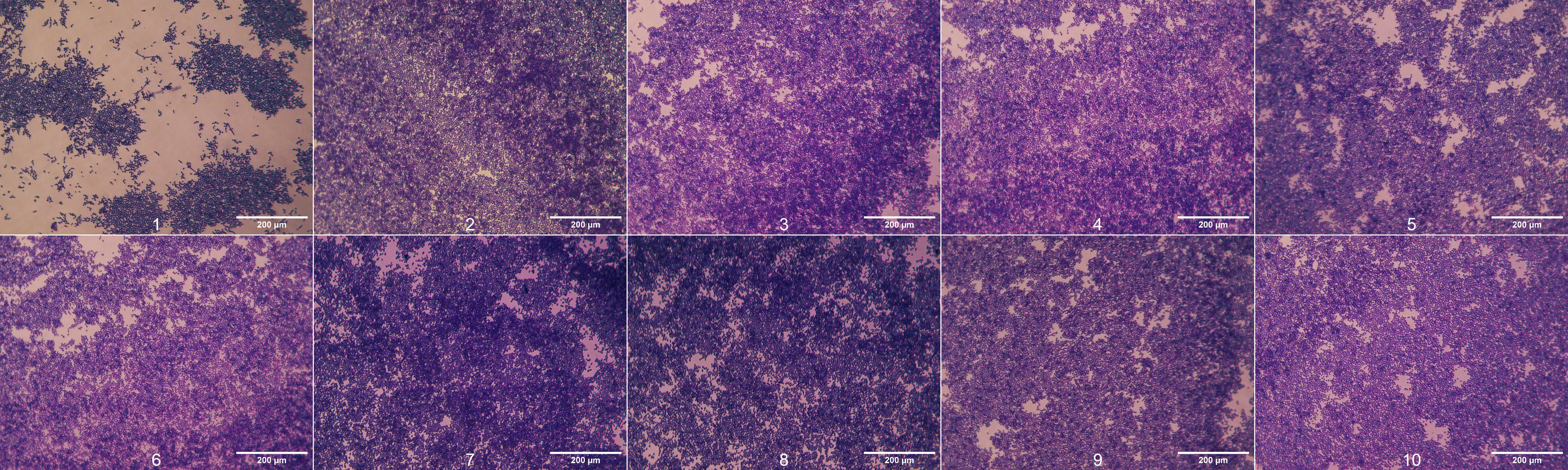

Supplement: Supplementary file 1 [file pharmaceutics-18-00508-s001.zip › Supplementary figures/Figure S5.tif]

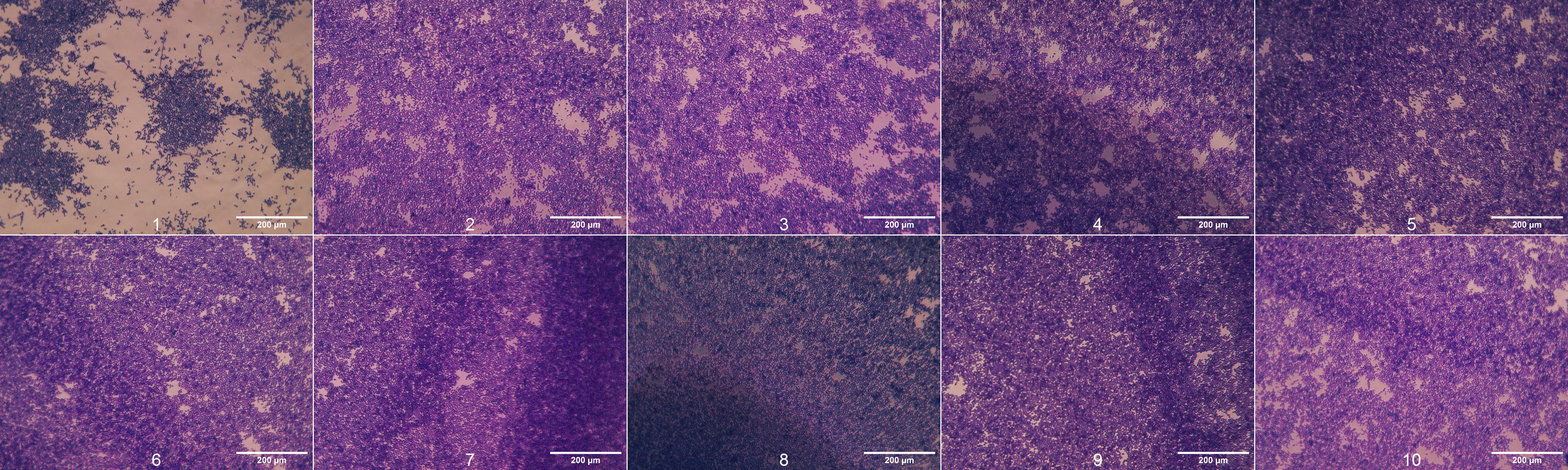

Supplement: Supplementary file 1 [file pharmaceutics-18-00508-s001.zip › Supplementary figures/Figure S6.tif]

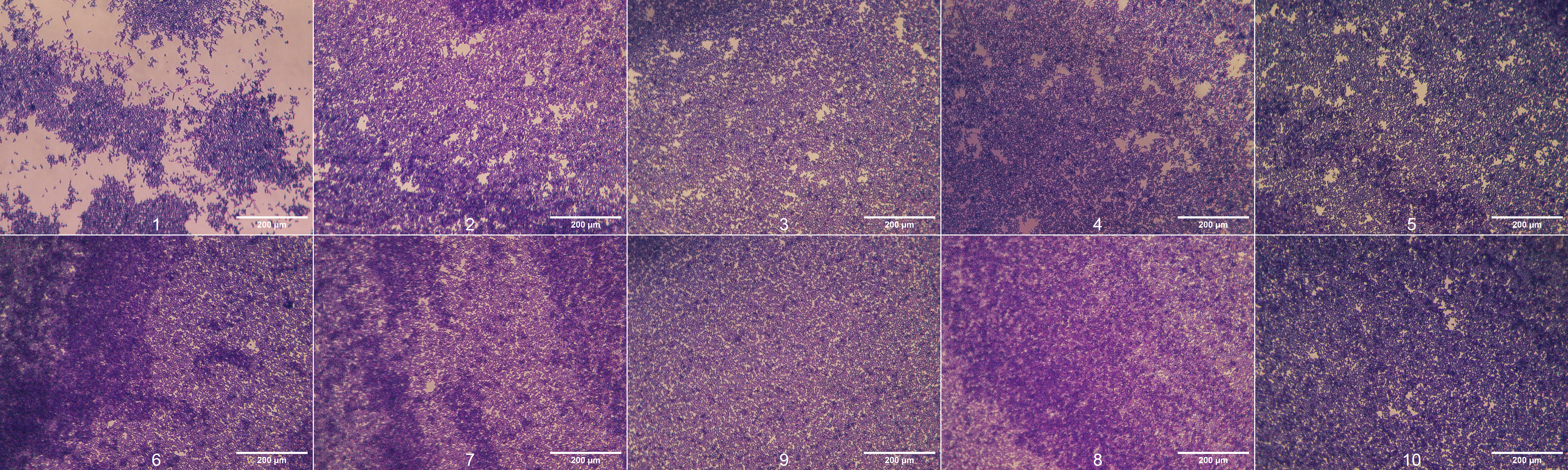

Supplement: Supplementary file 1 [file pharmaceutics-18-00508-s001.zip › Supplementary figures/Figure S7.tif]

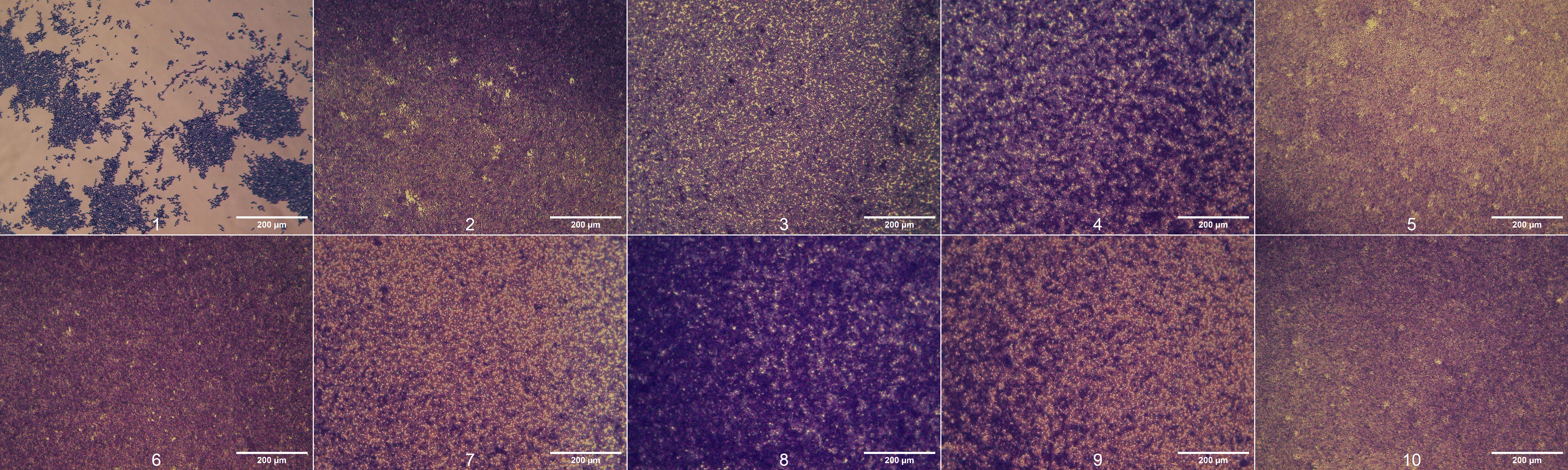

Supplement: Supplementary file 1 [file pharmaceutics-18-00508-s001.zip › Supplementary figures/Figure S8.tif]

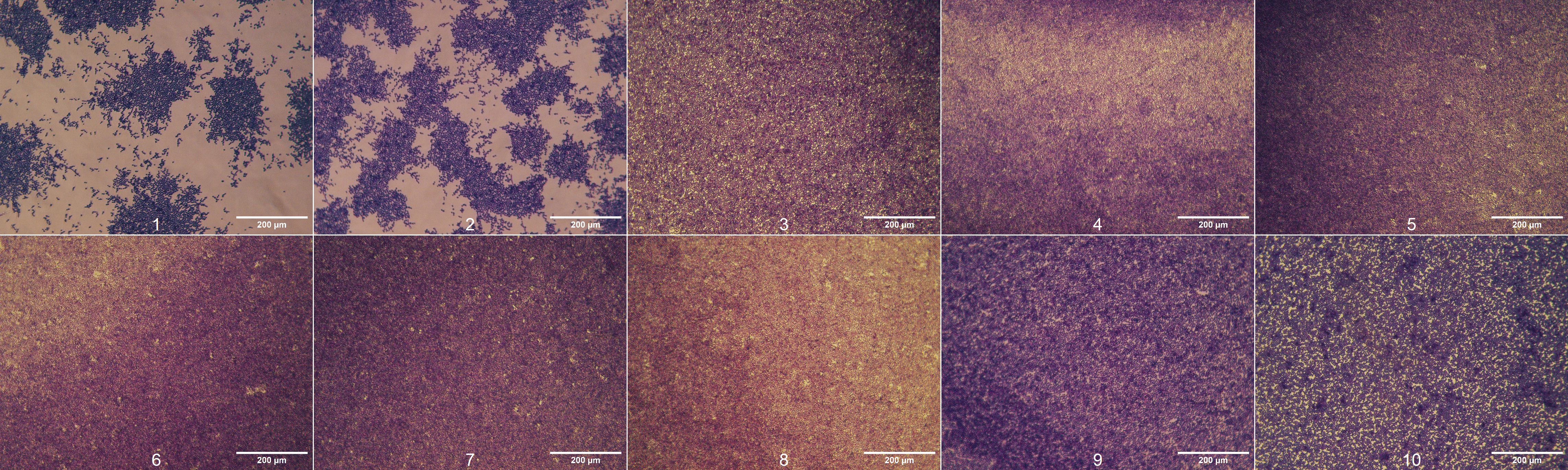

Supplement: Supplementary file 1 [file pharmaceutics-18-00508-s001.zip › Supplementary figures/Figure S9.tif]
